# Proteomics of *Porphyromonas gingivalis *within a model oral microbial community

**DOI:** 10.1186/1471-2180-9-98

**Published:** 2009-05-19

**Authors:** Masae Kuboniwa, Erik L Hendrickson, Qiangwei Xia, Tiansong Wang, Hua Xie, Murray Hackett, Richard J Lamont

**Affiliations:** 1 Department of Preventive Dentistry, Osaka University Graduate School of Dentistry, Osaka, Japan; 2 Department of Chemical Engineering, University of Washington, Seattle, WA, USA; 3 Department of Microbiology, University of Washington, Seattle, WA, USA; 4 School of Dentistry, Meharry Medical College, Nashville, TN, USA; 5 Department of Oral Biology, University of Florida, Gainesville, FL, USA; 6 University of Wisconsin-Madison, Department of Chemistry, Madison, WI, USA

## Abstract

**Background:**

*Porphyromonas gingivalis *is a periodontal pathogen that resides in a complex multispecies microbial biofilm community known as dental plaque. Confocal laser scanning microscopy showed that *P. gingivalis *can assemble into communities in vitro with *Streptococcus gordonii *and *Fusobacterium nucleatum*, common constituents of dental plaque. Whole cell quantitative proteomics, along with mutant construction and analysis, were conducted to investigate how *P. gingivalis *adapts to this three species community.

**Results:**

1156 *P. gingivalis *proteins were detected qualitatively during comparison of the three species model community with *P. gingivalis *incubated alone under the same conditions. Integration of spectral counting and summed signal intensity analyses of the dataset showed that 403 proteins were down-regulated and 89 proteins up-regulated. The proteomics results were inspected manually and an ontology analysis conducted using DAVID. Significant decreases were seen in proteins involved in cell shape and the formation of the cell envelope, as well as thiamine, cobalamin, and pyrimidine synthesis and DNA repair. An overall increase was seen in proteins involved in protein synthesis. HmuR, a TonB dependent outer membrane receptor, was up-regulated in the community and an *hmuR *deficient mutant was deficient in three species community formation, but was unimpaired in its ability to form mono- or dual-species biofilms.

**Conclusion:**

Collectively, these results indicate that *P. gingivalis *can assemble into a heterotypic community with *F. nucleatum *and *S. gordonii*, and that a community lifestyle provides physiologic support for *P. gingivalis*. Proteins such as HmuR, that are up-regulated, can be necessary for community structure.

## Background

The microbial communities that exist on oral surfaces are complex and dynamic biofilms that develop through temporally distinct patterns of microbial colonization [1,2]. For example, initial colonizers of the salivary pellicle on the coronal tooth surface are principally commensal oral streptococci such as *S. gordonii *and related species. Establishment of these organisms facilitates the subsequent colonization of additional gram-positives along with gram-negatives such as *Fusobacterium nucleatum*. As the biofilm extends below the gum line and becomes subgingival plaque, further maturation is characterized by the colonization of more pathogenic gram-negative anaerobes including *Porphyromonas gingivalis *[2-4]. While organisms such as *P. gingivalis *are considered responsible for destruction of periodontal tissues, pathogenicity is only expressed in the context of mixed microbial communities. Periodontal diseases, therefore, are essentially microbial community diseases, and the interactions among the constituents of these communities and between the communities and host cells and tissues, are of fundamental importance for determining the health or disease status of the periodontium.

Oral biofilm developmental pathways are driven by coadhesive, signaling and metabolic interactions among the participating organisms. Pioneer bacteria provide a substratum and appropriate metabolic support for succeeding organisms. Complex consortia then accumulate through recognition and communication systems. These interbacterial signaling processes can be based on cell-cell contact, short range soluble mediators, AI-2, or nutritional stimuli [2,5-8]. In general, bacterial adaptation to the community lifestyle is accompanied by distinct patterns of gene and protein expression [9,10]. In *S. gordonii *for example, arginine biosynthesis genes are regulated in communities with *Actinomyces naeslundii *which enables aerobic growth when exogenous arginine is limited [11]. Over 30 genes are differentially regulated in *P. gingivalis *following community formation with *S. gordonii *but not with *S. mutans *[12], whereas in monospecies *P. gingivalis *biofilm communities there are changes in abundance of over 80 envelope proteins [13].

While over 700 species or phylotypes of bacteria can be recovered from the oral cavity, in any one individual there are closer to 200 species [14] and the diversity of bacteria assembled in dense consortia will be further limited by nutritional and other compatibility constraints. *P. gingivalis *can accumulate into single species biofilms and mixed species consortia with *S. gordonii *and related oral streptococci [15-17]. Moreover, introduction of *P. gingivalis *into the mouths of human volunteers results in almost exclusive localization in areas of streptococcal-rich plaque [18]. Development of more complex multi-species communities in aerated environments such as supragingival tooth surfaces may require oxygen scavenging by *F. nucleatum *[19]. *F. nucleatum *is also able to coaggregate with *P. gingivalis *and with oral streptococci [19-21]. Hence communities of *S. gordonii*, *F. nucleatum *and *P. gingivalis *are likely to be favored *in vivo*; however, community formation by these three organisms has not been investigated. The aim of this study was to examine the ability of *S. gordonii*, *F. nucleatum *and *P. gingivalis *to form multispecies communities *in vitro*, and to utilize a global proteomic approach to investigate differential protein expression in *P. gingivalis *in response to presence of these organisms.

## Results and discussion

### Assembly of *P. gingivalis-F. nucleatum-S. gordonii *communities in vitro

**Figure 1:**
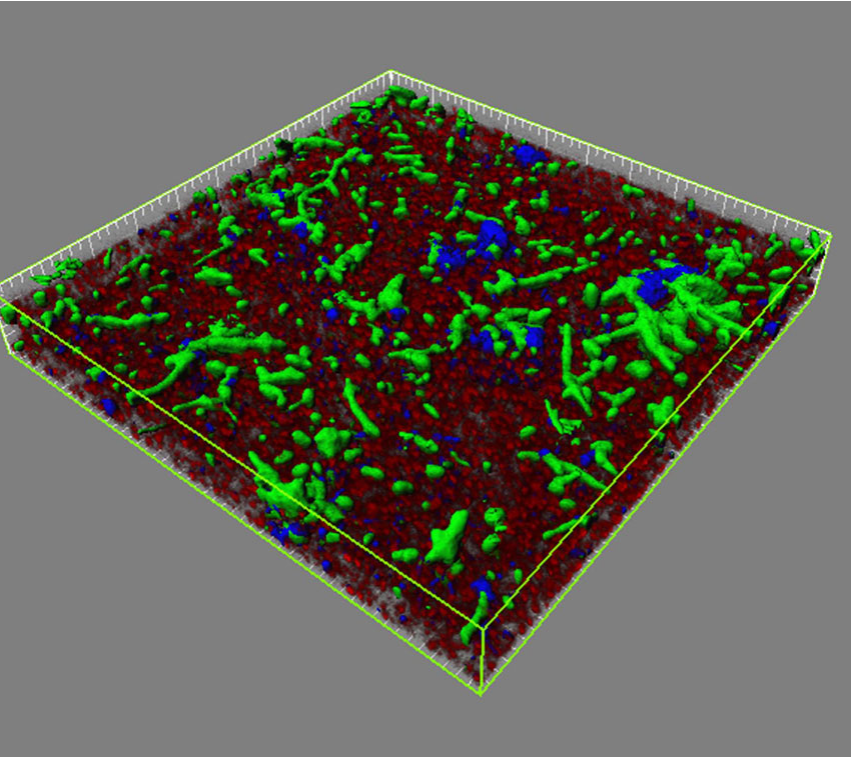
**Confocal laser scanning microscopy of *P. gingivalis*-*F. nucleatum*-*S. gordonii *community**. *S. gordonii *cells (red, stained with hexidium iodide) were cultured on a glass plate. FITC-labeled *F. nucleatum *cells (green), followed by DAPI labeled *P. gingivalis *cells (blue), were reacted sequentially with the *S. gordonii *substratum. Bacterial accumulations were examined on a Bio-Rad Radiance 2100 confocal laser scanning microscope. A series of fluorescent optical x-y sections in the z-plane to the maximum vertical extent of the accumulation were collected with Laser Sharp software. Images were digitally reconstructed with Imaris software. Image is representative of three independent experiments.

Confocal laser scanning microscopy (CLSM) was used to investigate the ability of *P. gingivalis *to assemble into communities with *S. gordonii *and *F. nucleatum*. In order to mimic the temporal progression of events in vivo, *S. gordonii *cells were first cultured on a glass surface and this streptococcal substratum was then reacted in succession with *F. nucleatum *and *P. gingivalis*. The *F. nucleatum *and *P. gingivalis *cells were maintained in the absence of growth media in order to be able to detect any metabolic support being provided by the other organisms in the community. A 3D reconstruction of the heterotypic community is shown in Fig. 1. Both *P. gingivalis *and *F. nucleatum *formed discrete accumulations and could be either separate from each other or interdigitated, consistent with the concept that the later gram-negative colonizers such as *P. gingivalis *and *F. nucleatum *initially establish themselves on the streptococcal rich supragingival plaque [4,18]. The results demonstrate the mutual compatibility of these three organisms for heterotypic community development, an early step in the overall process of plaque biofilm accumulation. Participation in multispecies communities may provide a basis for synergistic interactions in virulence. For example, mixed infections of *P. gingivalis *and *F. nucleatum *are more pathogenic in animal models than either species alone [22], and *F. nucleatum *can enhance the ability of *P. gingivalis *to invade host cells [23].

### Proteome of *P. gingivalis *in a three species community

**Figure 2:**
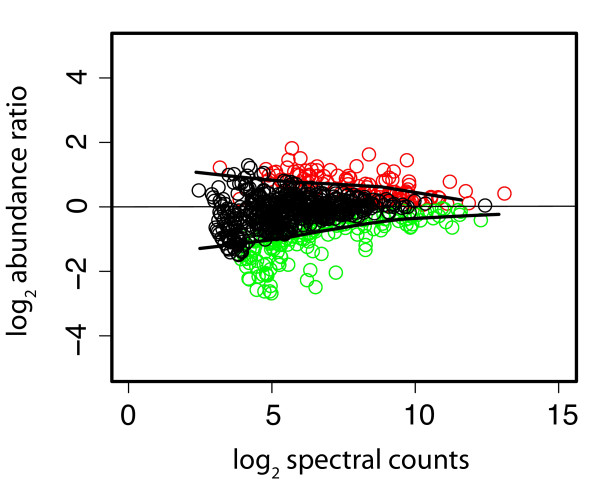
**Pseudo M versus A plot **[28,29]** of the average protein abundance ratios over all replicates for the *P. gingivalis*-*F. nucleatum-S. gordonii*/*P. gingivalis *comparison versus total abundance as estimated by spectral counting**. Color codes: red, *P. gingivalis *protein is over-expressed in the *P. gingivalis*-*F. nucleatum*-*S. gordonii *community relative to *P. gingivalis *alone; green, *P. gingivalis *protein is under-expressed in the community relative to *P. gingivalis *alone; black, no significant abundance change. Solid black lines represent a LOWESS curve fit [30] to the biological replicates of *P. gingivalis *alone, and represent the upper and lower boundaries of the experimentally observed error regions or null distributions associated with the relative abundance ratio calculations. Proteins coded as either red or green were determined to be significantly changed at the *q*-value [24] cut-off value of 0.01. Thus, the *G*-test predictions [56] were in good agreement with the curve fitting procedure. Details regarding hypothesis testing procedures can be found in Methods and in the explanatory notes to the data tables [see Additional File 1].

**Figure 3:**
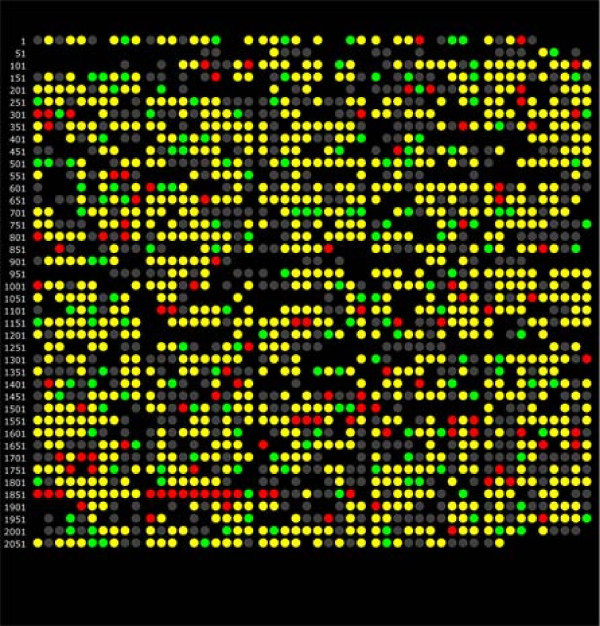
**Genomic representation of the *P. gingivalis *proteome, showing changes in relative abundance for the *P. gingivalis*-*F. nucleatum*-*S. gordonii*/*P. gingivalis *comparison by spectral counting**. Each dot represents a PGN ORF number in the order followed by the ATCC 33277 strain annotation. Color codes: red, over-expression in the *P. gingivalis*-*F. nucleatum*-*S. gordonii *community relative to *P. gingivalis *alone; green, under-expression in the community relative to *P. gingivalis *alone; yellow, protein was detected qualitatively, but did not change in abundance; gray, proteins that were qualitative non-detects; gaps indicate ORFs that were not common to both the ATCC 33277 and W83 annotations according to a master cross-reference compiled by LANL (G. Xie, personal communication).

To begin to investigate the mechanisms of adaptation of *P. gingivalis *to a community environment, the proteome of non-growing *P. gingivalis *cells incorporated into a community with *F. nucleatum *and *S. gordonii *was compared to the proteome of non-growing *P. gingivalis *cells alone. The expressed proteome of *P. gingivalis *in a community consisted of 1156 annotated gene products detected qualitatively. Based on spectral counting, 271 gene products showed evidence of relative abundance change at a *q*-value of 0.01: 109 proteins at higher relative abundance and 162 at lower relative abundance, using *P. gingivalis *alone as a reference state. Spectral counting is a conservative measure of protein abundance change that tends to generate low FDRs [24-26] but that often suffers from high FNRs in studies of the kind described here [27]. Less conservative calculations based on intensity measurements [27] found 458 gene products with evidence of relative abundance change at a *q*-value of 0.01: 72 proteins at higher relative abundance, and 386 proteins at lower relative abundance. Spectral counting and protein intensity measurements were examined for common trends. Trends tended to be consistent across both biological replicates, but the magnitudes of the abundance ratios showed significant scatter, similar to most published expression data at either the mRNA or protein level [27]. In most cases the abundance ratio trends were the same, using both quantitation methods, although not necessarily significantly so. In only eight cases were the spectral counting trend and summed intensity trend significantly in opposite directions for the same protein (PGN 0329, 0501, 1094, 1341, 1637, 1733, 2065). The integrated relative abundance trends found 403 gene products with evidence of lower relative abundance change and 89 at higher relative abundance. For purposes of examining the totals for combined trends, if an abundance change was called as significant (red or green in Additional file 1: Table ST1) in one measurement, it was considered significant for the above combined totals only if the ratio of the other measurement showed the same direction of abundance change, with a log_2 _ratio of ± 0.1 or greater regardless of the *q*-value in the second measurement. The experimental data for differential protein abundance are shown in Fig. 2 as a pseudo M/A plot [28,29] with a LOWESS curve fit [30]. The same data are plotted in Fig. 3 as open reading frames according to PGN numbers from the ATCC 33277 genome annotation [31]. A complete listing of all proteins, their abundance ratios relative to *P. gingivalis *controls incubated alone under the same conditions as determined by spectral counting and summed signal intensity [27,32,33], and *q*-values, are given in Additional file 1: Table ST1. Qualitative identifications for proteins secreted by *P. gingivalis *in the 3-species community but not by *P. gingivalis *alone are given Additional file 1: Table ST2. Additional file 1: Figs. SF1, SF2, SF3, SF4, SF5 and SF6 and explanatory notes provide more detailed technical information regarding reproducibility of the biological replicates and the adequacy of sampling depth. To assess global sampling depth, average spectral counts were calculated by summing all spectral count numbers for all *P. gingivalis *proteins in the FileMaker script output described under Methods and dividing by the total number of *P. gingivalis *proteins in that file. The average redundant spectral count number for peptides unique to a given ORF for *P. gingivalis *alone was 80, for *P. gingivalis *in the community it was 64. The lower number of counts observed for *P. gingivalis *proteins in the community is consistent with the added sampling demands placed on the analytical system by sequence overlaps in the proteomes of all three microbes and thus the smaller number of unique proteolytic fragments predicted. More discussion of this topic is given in the explanatory notes [see Additional File 1]. Spectral count values for individual proteins are given in data Additional file 1: Table ST1. Details regarding access to mass spectrometry data for individual peptides and their SEQUEST database searching scores [34], *p*-values and *q*-values are given in the notes to the data tables [see Additional File 1].

### Proteins and functions differentially regulated by *P. gingivalis *in a community

#### Cell envelope and cell structure

In bacterial communities significant surface-surface contact occurs both within and among accumulations of the constituent species, as was also observed in the *P. gingivalis*-*F. nucleatum*-*S. gordonii *consortia. Regulation of outer membrane constituents of *P. gingivalis *would thus be predicted in the context of a community and this was borne out by the proteomic results. Overall, 84 proteins annotated as involved in the cell envelope were detected, and 40 of these showed reduced abundance in the three species community, indicating an extensive change to the cell envelope. Only four proteins showed increased abundance, two OmpH proteins (PGN0300, PGN0301) and two lipoproteins (PGN1037, PGN1998). MreB (PGN0234), a bacterial actin homologue that plays a role in determining cell shape, showed almost a 2-fold decrease in community derived *P. gingivalis*. Expression of MreB has been found to decrease under stress or during stationary phase in *Vibrio paraheamolyticus *[35]. However, stress-related proteins were generally reduced in *P. gingivalis *cells in the community (see below) so stress is an unlikely explanation for the change in MreB. Rather, the decrease in MreB abundance may be due to the *P. gingivalis *cells entering a state resembling stationary phase or responding in a previously unseen way to the formation of the three species community.

#### Protein synthesis

Extensive changes were observed in ribosomal proteins and in translation elongation and initiation proteins. While overall more proteins showed reduced abundance in the three species community, the changes to the translational machinery were almost exclusively increases in abundance. Of 49 ribosomal proteins detected, 27 showed increased abundance, while only one showed decreased abundance. Of nine translation elongation and initiation proteins detected, none showed significant abundance decreases but five showed increased abundance (EfG (PGN1870), putative EfG (PGN1014), EfTs (PGN1587), EfTu (PGN1578), and If2 (PGN0255)). This represents not only a substantial portion of the translational machinery but also a large portion, 36%, of the proteins showing increased abundance. It is well known that ribosomal content is generally proportional to growth rate [36]; however, given that the cells were not in culture medium during the assay, rapid growth is an unlikely explanation for these results. The increased ribosomal content presumably indicates increased translation, consistent with the community providing physiologic support to *P. gingivalis *and allowing higher levels of protein synthesis.

#### Vitamin synthesis

**Figure 4:**
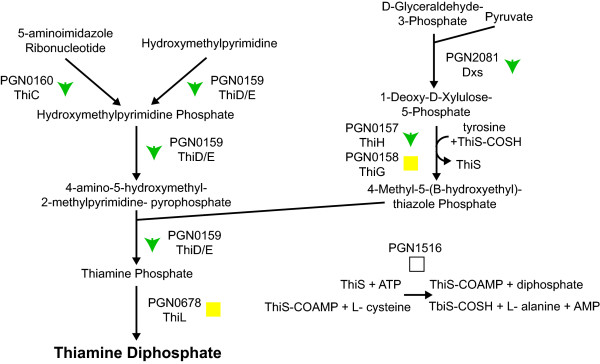
**Thiamine biosynthetic pathway, showing protein abundance changes for the *P. gingivalis*-*F. nucleatum*-*S. gordonii*/*P. gingivalis *comparison**. Proteins catalyzing each step in the pathway are shown by their *P. gingivalis *ATCC 33277 gene designation (PGN number) and protein name, where applicable. Green downward arrows indicate decreased abundance in the three species community. Yellow squares indicate no statistically significant abundance change. Empty squares indicate that the protein was not detected in the proteomic analysis. Thiamine diphosphate is shown in bold.

Pathways for synthesizing several vitamins showed reduced protein abundance in the three species community. Most of the proteins involved in thiamine diphosphate (vitamin B1) biosynthesis were downregulated (Fig. 4). Thiamine is a cofactor for the 2-oxoglutarate dehydrogenase complex that converts 2-oxoglutarate to succinyl-CoA and for the transketolase reactions of the anaerobic pentose phosphate pathway [37]. However, transketolase (PGN1689, Tkt) showed no abundance change while of the three components of the 2-oxoglutarate dehydrogenase complex (PGN1755, KorB) only the beta subunit showed an abundance increase.

Only incomplete pathways have been identified for many of the other vitamin biosynthesis activities in *P. gingivalis*. However, cobalamin (vitamin B12) synthesis [38] can be predicted to be decreased in the community, with five (PGN0010, CobC; PGN0316, CbiG; PGN0317, CobL; PGN0318, CobH/CbiC; PGN0735, CobU) of the seven identified proteins having statistically significant reductions. Less complete population of pathways was observed for pyridoxal phosphate (vitamin B6) and biotin synthesis. Only two of the four detected proteins for vitamin B6 synthesis showed reduced abundance (PGN1359, PdxB and PGN2055, PdxA). For biotin synthesis, three of the six detected proteins showed reduced abundance (PGN0133, BioA; PGN1721, BioF; PGN1997, BioD). None of the vitamin/cofactor synthesis pathways showed any indication of increased protein levels in the three species community.

The decrease in several vitamin/cofactor pathways could be due to a decreased utilization of those cofactors. However, in the case of thiamine, the proteins that utilize this cofactor showed no decrease, and a possible increase in abundance, implying that demand for vitamin B1 was unchanged. A more likely explanation for the reduced cofactor pathways is therefore nutrient transfer. Either one or both of the other organisms in the three species community could be providing *P. gingivalis *with cofactors, allowing reduced cofactor synthesis without reducing expression of the cofactor dependent pathways. Nutritional cross-feeding among members of oral biofilms is well established [5], and indeed *P. gingivalis *has been found to utilize succinate produced by *T. denticola *[39].

#### Nucleotide synthesis

**Figure 5:**
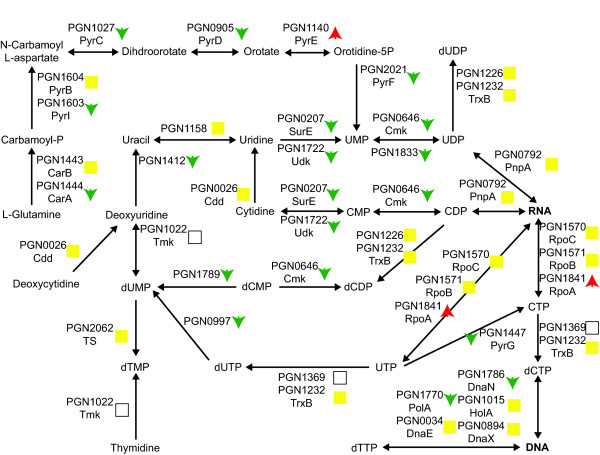
**Pyrimidine biosynthetic pathway, showing protein abundance changes for the *P. gingivalis*-*F. nucleatum*-*S. gordonii*/*P. gingivalis *comparison**. The protein names follow the same conventions as in Fig. 4. Green downward arrows indicate decreased abundance in the three species community. Red upward arrows indicate increased abundance. Yellow squares indicate no statistically significant abundance change. Empty squares indicate that the protein was not detected in the proteomic analysis. RNA and DNA are shown in bold.

Pyrimidine biosynthesis appeared to be reduced in the three species community (Fig. 5) as many of the proteins leading to the production of finished pyrimidine nucleotides have decreased abundance. However, the proteins responsible for incorporating finished ribonucleotides into RNA show unchanged or increased abundance. As with vitamin biosynthesis this may be the result of nutrient transfer from the other organisms in the community. *P. gingivalis *can acquire nucleosides and nucleobases and it has even been suggested that they may represent an important nutrient source for *P. gingivalis *[40]. Consistent with uptake of nucleosides and their precursors, uracil permease (PGN1223) shows increased expression in the three species community.

**Figure 6:**
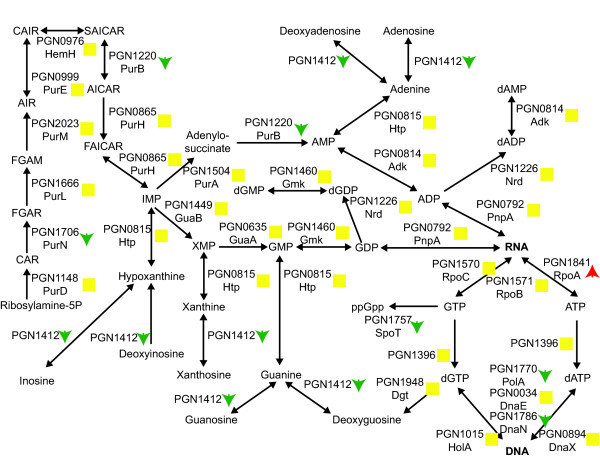
**Purine biosynthetic pathway, showing protein abundance changes for the *P. gingivalis*-*F. nucleatum*-*S. gordonii*/*P. gingivalis *comparison**. The protein names and arrows/squares follow the same conventions as in Fig. 5. RNA and DNA are shown in bold. GAR: 5-Phosphoribosyl glycinamide; FGAM: 5-phosphoribosyl-N-formylglycineamidine; FGAR: 1-(5'-Phosphoribosyl)-N-formylglycinamide; AICAR: 5'-phosphoribosyl-4-(N-succinocarboxamide)-5-aminoimidazole; AIR: 1-(5'-Phophoribosyl)-5-aminoimidazole; CAIR: 5'P-Ribosyl-4-carboxy-5-aminoimidazole; SAICAR: 5'P-Ribosyl-4-(N-succinocarboximide)-5-aminoimidazole; FAICAR: 1 (5'-Phosphoribosyl)-5-formamido-4-imidazole carboxamide.

Purine biosynthesis does not appear to be significantly effected in the three species community (Fig. 6). A few proteins showed reduced abundance, but the central biosynthesis pathway was primarily unchanged.

#### Stress proteins

The ability of the community to provide physiologic support to constituent species might result in *P. gingivalis *experiencing lower levels of environmental stress than occurs in monoculture. Consistent with this concept, community derived *P. gingivalis *showed a significant reduction in abundance of DNA repair proteins (PGN0333, RadA; PGN0342, Ung; PGN0367, Xth; PGN1168, MutS; PGN1316, UvrA; PGN1388, LigA; PGN1567, RecF; PGN1585, UvrB; PGN1712, Nth; PGN1714, Mfd; PGN1771, Pol1). DNA repair genes are generally induced in the presence of damaged DNA [41], and lower abundance of DNA repair proteins is consistent with the monoculture experiencing more DNA damage than *P. gingivalis *in the three species community where the presence of the partner organisms provides protection against DNA damage.

Only two stress proteins showed increased abundance, and then only 30% increases, the molecular chaperone DnaK (PGN1208) and a PhoH family protein possibly involved in oxidation protection (PGN0090).

### Role of the differentially regulated *P. gingivalis *protein HmuR

**Figure 7:**
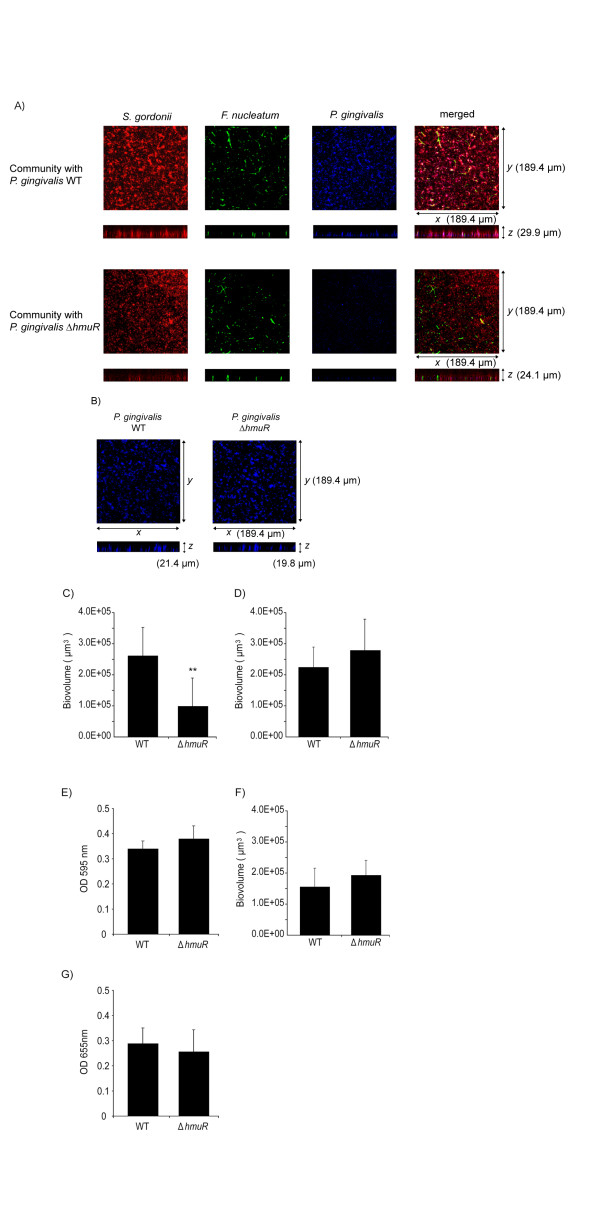
**HmuR mutant of *P. gingivalis *is deficient in community accumulation**. A) Confocal microscopy showing x-y and x-z projections of communities of *S. gordonii *(red), *F. nucleatum *(green) and *P. gingivalis *(blue) wild type (WT) or Δ*hmuR *mutant strains. Representative image from three independent experiments. B) Confocal microscopy showing x-y and x-z projections of single species *P. gingivalis *WT or Δ*hmuR *mutant accumulations. Representative image from three independent experiments. C) Biovolume analysis of *P. gingivalis *WT or Δ*hmuR *mutant accumulation in the *P. gingivalis-F. nucleatum-S. gordonii *communities shown in A. D) Biovolume analysis of *P. gingivalis *WT or Δ*hmuR *single species accumulations shown in B. E) Biomass of *P. gingivalis *WT or Δ*hmuR *single species accumulations measured by crystal violet staining and release. F) Biovolume analysis of *P. gingivalis *WT or Δ*hmuR *accumulation in two species *P. gingivalis-S. gordonii *communities. G) Biomass of *P. gingivalis *WT or Δ*hmuR *two species accumulation with *F. nucleatum *measured with *P. gingivalis *antibodies. ** denotes p < 0.01 (n = 3) compared to WT.

To begin to test the functional relevance of proteins identified as differentially regulated in the three species community, we undertook a mutational analysis. For this purpose it was important to target a protein that directly effectuates a biological function and lacks homologs in the genome. HmuR, a major hemin uptake protein, and potential adhesin [42], was selected. As shown in Fig. 7A, while wild type *P. gingivalis *cells are abundant within a *S. gordonii*-*F. nucleatum*-*P. gingivalis *community, *P. gingivalis *cells lacking HmuR are deficient in community formation. Biovolume analysis showed a 70% reduction in community formation by the HmuR mutant (Fig. 7C). Furthermore, this effect was specific for the three species community as a decrease in accumulation by the HmuR deficient mutant was not observed in monospecies biofilms, or in two species communities of *P. gingivalis *with either *S. gordonii *or *F. nucleatum *(Fig. 7B, D–G). Hence loss of HmuR, that is up-regulated by *P. gingivalis *when the organism is associated with *S. gordonii *and *F. nucleatum*, results in a phenotype that is restricted to three species community formation. *P. gingivalis *cells were first cultured in hemin excess, under which conditions the *hmu *operon is expressed at a basal level [42]. As the three species model system involves metabolically quiescent *P. gingivalis *cells in buffer, it is unlikely that the role of HmuR is related to its hemin uptake capacity. However, TonB dependent receptors can exhibit functions distinct from transport across the outer membrane. For example, in *E. coli *the TonB dependent catecholate siderophore receptor Iha confers an adhesin function and contributes to colonization and virulence in the mouse urinary tract [43]. Hence, HmuR may have a cohesive function in community formation by *P. gingivalis *although further studies are necessary to resolve this issue.

## Conclusion

Complex multi-species biofilms such as pathogenic dental plaque accumulate through a series of developmental steps involving attachment, recruitment, maturation and detachment. Choreographed patterns of gene and protein expression characterize each of these steps. In this study we developed a model of the early stages of plaque development whereby three compatible species accreted into simple communities. *P. gingivalis *increased in biomass due to attachment and recruitment, and this allowed us to catalog differential protein expression in *P. gingivalis *consequent to contact dependent interbacterial signaling and communication through short range soluble mediators. The proteomic analysis indicated that around 40% of *P. gingivalis *proteins exhibit changes in abundance in a community with *F. nucleatum *and *S. gordonii*, implying extensive interactions among the organisms. The proteomic results were consistent with the formation of a favorable environment in a *P. gingivalis*-*F. nucleatum*-*S. gordonii *community, wherein *P. gingivalis *showed evidence of increased protein synthesis and decreased stress. Moreover, nutrient transfer may occur among the constituents of the community. As evidenced by HmuR, these proteins may have a functional role in the development of multispecies communities and ultimately shape the pathogenic potential of plaque.

## Methods

### Bacteria and culture conditions

*Fusobacterium nucleatum *subsp. *nucleatum *ATCC 25586 and *Porphyromonas gingivalis *ATCC 33277 were grown anaerobically (85% N_2_, 10% H_2_, 5% CO_2_) at 37°C in trypticase soy broth supplemented with 1 mg/ml yeast extract, 1 μg/ml menadione and 5 μg/ml hemin (TSB). *S. gordonii *DL1 was grown anaerobically at 37°C in Todd-Hewitt broth (THB).

### Chemicals

HPLC grade acetonitrile was from Burdick & Jackson (Muskegon, MI, USA); high purity acetic acid (99.99%) and ammonium acetate (99.99%), from Aldrich (Milwaukee, WI, USA). High purity water was generated with a NANOpure UV system (Barnstead, Dubuque, IA, USA).

### Proteomics of model bacterial communities

High density bacterial communities were generated by the method of Merritt et al. [44]. Bacteria were cultured to mid-log phase, harvested by centrifugation and resuspended in pre-reduced PBS (rPBS). 1 × 10^9 ^cells of *P. gingivalis *were mixed with an equal number of *S. gordonii *and *F. nucleatum *as a combination of the three species. *P. gingivalis *cells alone were also used as a control. Two independent biological replicates from separate experiments comprised of at least two technical replicates were analyzed. Bacteria were centrifuged at 3000 g for 5 min, and pellets were held in 1 ml pre-reduced PBS in an anaerobic chamber at 37°C for 18 h. The bacterial cells remain viable under these conditions, as determined by both colony counts and live/dead fluorescent staining. Supernatant and bacterial cells were separated and processed separately. Bacterial cells were lysed with ice cold sterile distilled water and proteins were digested with trypsin as previously described for *P. gingivalis *[33], then fractionated on a 2.0 mm × 150 mm YMC polymer C18 column. There were five pre-fractions collected for each cellular sample, with a final volume of 50 μl for each fraction. The 2D capillary HPLC/MS/MS analyses [32,45,46] were conducted using an in-house fabricated semi-automated system, consisting of a Thermo LTQ mass spectrometer (Thermo Fisher Corp. San Jose, CA, USA), a Magic 2002 HPLC (Michrom BioResouces, Inc., Auburn, CA, USA), a Pump 11 Plus syringe pump (Harvard Apparatus, Inc., Holliston, MA, USA), an Alcott 718 autosampler (Alcott Chromatography, Inc., Norcross, GA, USA) and a micro-electrospray interface built in-house. About 2 μl of sample solution was loaded into a 75 μm i.d. × 360 μm o.d. capillary column packed with 11 cm of AQUA C18 (5 μm, Phenomenex, Torrance, CA, USA) and 4 cm of polysulfoethyl aspartamide SCX (strong cation exchange) resin (PSEA, 5 μm, Michrom BioResouces, Inc.). The peptides were eluted with a seven step salt gradient (0, 10, 25, 50, 100, 250 and 500 mM ammonium acetate) followed by an acetonitrile gradient elution (Solvent A: 99.5% water, 0.5% acetic acid. Solvent B: 99.5% acetonitrile, 0.5% acetic acid), 5% B hold 13 min, 5–16% B in 1 min, hold 6 min, 16–45% B in 45 min, 40–80% B in 1 min, hold 9 min, 80–5% B in 5 min, then hold 10 min. For the secreted proteins in the supernatant no pre-fractionation or SCX was performed, and 4 μl of digested sample was loaded into a 75 μm i.d. × 360 μm o.d. column packed with 11 cm AQUA C18 for a single dimension of capillary HPLC/tandem MS analysis. After 20 min of flushing with 5% acetonitrile, peptides were eluted by an acetonitrile gradient (5–12% B in 1 min, hold 9 min, 12–40% B in 50 min, 40–80% B in 1 min, hold 10 min, 80–5% B in 5 min, hold 14 min). The MS^1 ^scan range for all samples was 400–2000 *m/z*. Each MS^1 ^scan was followed by 10 MS^2 ^scans in a data dependent manner for the 10 most intense ions in the MS^1 ^scan. Default parameters under Xcalibur 1.4 data acquisition software (Thermo Fisher) were used, with the exception of an isolation width of 3.0 *m/z *units and a normalized collision energy of 40%.

### Data processing and protein identification

Raw data were searched by SEQUEST [34] against a FASTA protein ORF database consisting of the Ver. 3.1 curation of *P. gingivalis *W83 (2006, TIGR-CMR [47]), *S. gordonii *Challis NCTC7868 (2007, TIGR-CMR [48], *F. nucleatum *ATCC 25586 (2002, TIGR-CMR [49]), bovine (2005, UC Santa Cruz), nrdb human subset (NCBI, as provided with Thermo Bioworks ver. 3.3) and the MGC (Mammalian Gene collection, 2004 curation, NIH-NCI [50]) concatenated with the reversed sequences. After data processing, the genome sequence for strain 33277 became available [31] and the data were subsequently cross-referenced to PGN numbers from the 33277 specific FASTA database provided by LANL (personal communication with G. Xie). Although Naito et al. [31] reported extensive genome re-arrangements between W83 and ATCC 33277, the actual protein amino acid sequences are sufficiently similar across the proteome that the use of a database based on W83 was not expected to greatly impact the analysis. Our proteomic methods are not sensitive to genome re-arrangements, only to changes in amino acid sequence for a given protein. The reversed sequences were used for purposes of calculating a peptide level qualitative FDR using the published method [51,52]. The SEQUEST peptide level search results were filtered and grouped by protein using DTASelect [53], then input into a FileMaker script developed in-house [32,33] for further processing. The DTASelect Ver. 1.9 filter parameters were: peptides were fully tryptic; ΔCn/Xcorr values for different peptide charge states were 0.08/1.9 for +1, 0.08/2.0 for + 2, and 0.08/3.3 for +3; all spectra detected for each sequence were retained (t = 0). Only peptides that were unique to a given ORF were used in the calculations, ignoring tryptic fragments that were common to more than one ORF or more than one organism, or both. In practice this had the consequence of reducing our sampling depth from what we have achieved with single organism studies [27,32,33], because the gene sequence overlap among the three organisms is significant. A bioinformatic analysis (data not shown) of inferred protein sequence overlaps between *P. gingivalis *and *S. gordonii *or *F. nucleatum *suggested the reduction in the number of predicted tryptic fragments unique to *P. gingivalis *would not be sufficient to impact the analysis of more than a small number of proteins. The qualitative peptide level FDR was controlled to approximately 5% for all conditions by selecting a minimum non-redundant spectral count cut-off number appropriate to the complexity of each condition, *P. gingivalis *alone or the *P. gingivalis*-*F. nucleatum-S. gordonii *community.

### Protein abundance ratio calculations

Protein relative abundances were estimated on the basis of summed intensity or spectral count values [27,32,33] for proteins meeting the requirements for qualitative identification described above. Summed intensity refers to the summation of all processed parent ion (peptide) intensity measurements (MS^1^) for which a confirming CID spectrum (MS^2^) was acquired according to the DTASelect filter files. For spectral counts, the redundant numbers of peptides uniquely associated with each ORF were taken from the DTAselect filter table (t = 0). Spectral counting is a frequency measurement that has been demonstrated in the literature to correlate with protein abundance [54]. These two ways of estimating protein relative abundance, that avoid the need for stable isotope labeling, have been discussed in a recent review [27] with specific reference to microbial systems. To calculate protein abundance ratios, a normalization scheme was applied such that the total spectral counts or total intensities for all *P. gingivalis *proteins in each condition were set equal for each comparison. This normalization also had the effect of zero centering the log_2 _transformed relative abundance ratios, see Fig. 2 (and also the frequency histograms in Additional file 1: Figs. SF5 and SF6). The normalized data for each abundance ratio comparison was tested for significance using either a global *G*-test or a global paired *t*-test for each condition, the details of which have been published for this type of proteomics data in which all biological replicates are compared against each other [55,56], and are also described in the explanatory notes [see Additional File 1]. Both of these testing procedures weigh deviation from the null hypothesis of zero abundance change and random scatter in the data to derive a probability or *p*-value that the observed change is a random event, i.e. that the null hypothesis of no abundance change is true. Each hypothesis test generated a *p*-value that in turn was used to generate a *q*-value as described [24,32], using the R package QVALUE [26]. The *q*-value in this context is a measure of quantitative FDR [25] that contains a correction for multiple hypothesis testing. A *q *cut-off value of 0.01 was used for all ratios reported in Additional file 1: Table ST1. All statistical calculations were done in R (Ver. 2.5.0), using source code that has been published [32,33,55]. Only proteins with data consisting of confirmed high scoring MS^2 ^mass spectra (high scoring qualitative database matches as described above) present in both the numerator and denominator of the abundance ratio comparison were listed as significantly changed in Additional file 1. Certain proteins listed in the tables with *q*-values = 0.01 are still coded yellow for no significant abundance change due to missing data in either the numerator or the denominator.

### Ontology analysis

An overall list of detected proteins as well as lists of proteins that showed increased or decreased levels in the three species community were prepared using Entrez gene identifiers. Ontology analyses were then conducted using the DAVID [57] functional annotation clustering feature with the default databases. Both increased and decreased protein level lists were analyzed using the overall list of detected proteins as the background. Potentially interesting clusters identified by DAVID were then examined manually.

### Construction of *P. gingivalis *HmuR mutant

A mutation in the *hmuR *gene was generated using ligation-independent cloning of PCR mediated mutagenesis (LIC-PCR) [58]. A 2.1-kb *ermF*-*ermAM *cassette was introduced into the *hmuR *gene by three steps of PCR to yield a *hmuR-erm-hmuR *DNA fragment as described previously [59]. The fragment was then introduced into *P. gingivalis *33277 by electroporation. The *hmuR *deficient mutant (Δ*hmuR*) was generated via a double crossover event that replaces *hmuR *with the *hmuR-erm-hmuR *DNA fragment in the 33277 chromosome. The mutants were selected on TSB plates containing erythromycin (5 μg/ml), and the mutation was confirmed by PCR analysis. Growth rates of mutant and parent strains were equivalent.

### Quantitative community development assays

i) Crystal violet assay. Homotypic community formation by *P. gingivalis *was quantified by a microtiter plate assay [60], as adapted for *P. gingivalis *[61]. Parental and mutant strains in early log phase (2 × 10^8 ^cells) were incubated at 37°C anaerobically for 24 h. Wells were washed, stained with 1% crystal violet and destained with 95% ethanol. Absorbance at 595 nm was determined in a Benchmark microplate reader. ii) ELISA. *F. nucleatum *was incubated at 37°C anaerobically for 36 h in microtiter plate wells. After washing, parental and mutant *P. gingivalis *strains (2 × 10^6 ^cells) were incubated with the fusobacterial biofilm at 37°C anaerobically for 24 h. *P. gingivalis *accumulation was detected with antibodies to whole cells (1:10,000) followed by peroxidase-conjugated secondary antibody (1:3,000), each for 1 h at 37°C. Antigen-antibody binding was determined by a colorimetric reaction using the 3,3',5,5'-tetramethylbenzidine (TMB) liquid substrate, and absorbance at 655 nm. *P. gingivalis *antibody binding to the fusobacterial biofilm alone was subtracted as background. iii) Confocal microscopy assay. A. Single species. *P. gingivalis *was stained with 4',6-diamidino-2-phenylindole (50 μg ml^-1^) and 2 × 10^6 ^cells in rPBS incubated anaerobically at 37°C for 16 h with rocking in individual chambers of the CultureWell coverglass system (Grace Bio Labs). Chambers were washed three times in rPBS. B. Dual species. Heterotypic *P. gingivalis*-*S. gordonii *communities were generated as described previously [15]. *S. gordonii *cells were labeled with hexidium iodide (15 μg ml^-1^), then cultured anaerobically at 37°C for 16 h with rocking in CultureWell chambers. *P. gingivalis *was stained with 5-(and-6)-carboxyfluorescein, succinimidyl ester (10 μg ml^-1^), and 2 × 10^6 ^cells in rPBS were reacted with the surface attached *S. gordonii *for 24 h anaerobically at 37°C with rocking. C) Three species. Surface attached hexidium iodide-stained *S. gordonii *were generated as above. Fluorescein stained *F. nucleatum *(2 × 10^6 ^cells in rPBS) reacted with *S. gordonii *for 24 h anaerobically at 37°C with rocking. The coverglass was then washed with rPBS to remove non-attached bacteria. *P. gingivalis *was stained with 4',6-diamidino-2-phenylindole (50 μg ml^-1^) and 2 × 10^6 ^cells in rPBS were added and further incubated for 24 h anaerobically at 37°C with rocking. Communities were observed on a Bio-Rad Radiance 2100 confocal laser scanning microscope (Blue Diode/Ar/HeNe) system with an Nicon ECLIPSE TE300 inverted light microscope and 40 × objective using reflected laser light of combined 405, 488 and 543 nm wavelengths where appropriate. A series of fluorescent optical x-y sections were collected to create digitally reconstructed images (*z*-projection of *x-y *sections) of the communities with Image J V1.34s (National Institutes of Health) or Laser Sharp software (Bio-Rad). Z stacks of the x-y sections of CLSM were converted to composite images with "Iso Surface" functions of the "Surpass" option on Imaris 5.0.1 (Bitplane AG; Zurich, Switzerland) software. Iso Surface images of *P. gingivalis *were created at threshold of 20 and smoothed with Gaussian Filter function at 0.5 width, and *P. gingivalis *biovolume was calculated.

Biofilm assays were repeated independently three times with each strain in triplicate. Crystal violet results were compared by *t*-tests. Biovolume calculations were compared with a *t*-test using the SPSS statistics software.

## Abbreviations

ATCC: American Type Culture Collection; DAVID: Database for Annotation, Visualization and Integrated Discovery; FDR: false discovery rate; FNR: false negative rate; LANL: Los Alamos National Laboratory; LOWESS: Locally weighted scatterplot smoothing; MS: Mass spectrometry; MS^1^: First stage of tandem mass spectrometry; MS^2^: Second stage of tandem mass spectrometry; SCX: strong cation exchange; TIGR-CMR: The Institute for Genomic Research Comprehensive Microbial Resource, now part of the J. Craig Venter Institute.

## Authors' contributions

MK carried out the community construction and analysis by confocal microscopy; ELH did the pathway analysis; QX and TW performed the protein biochemistry, separations and mass spectrometry; HX constructed the *hmuR *mutant; MH and RJL conceived the experiments. MH, ELH, MK and RJL wrote the manuscript. MK and ELH contributed equally.

## Supplementary Material

Additional file 1 **DataTables**. Data tables, explanatory notes and supporting figures. This file contains the proteomic data tables ST1 and ST2, explanatory notes for each heading in the tables, a note regarding the handling of missing data and additional figures informative of proteome coverage for the model community described in data tables ST1 and ST2. Click here for file
