# Supplementary material for: Proteomics of Porphyromonas gingivalis within a model oral microbial community
Source: BMC Microbiol. 2009 May 19;9:98. doi: 10.1186/1471-2180-9-98 (PMC2689231; doi:10.1186/1471-2180-9-98)
Supplement: Additional file 1 — DataTables. Data tables, explanatory notes and supporting figures. This file contains the proteomic data tables ST1 and ST2, explanatory notes for each heading in the tables, a note regarding the handling of missing data and additional figures informative of proteome coverage for the model community described in data tables ST1 and ST2. [file 1471-2180-9-98-S1.pdf]

Additional Material for M. Kuboniwa *et al.*

Contents:

Instructions for accessing *DTASelect* filter files S-2

Notes and explanatory material for the data tables S-2

A note regarding sampling depth and missing data S-5

Notes and explanatory material for the additional figures S-6

Table ST1 .... PgSgFn/Pg, cellular proteins S-7

Table ST2 .... secreted proteins S-56

Reconstructed array plots in ATCC 33277 ORF order,
Additional figures SF1-SF4 .... S-62

Frequency Histograms, additional figures SF5, SF6 .... S-66

Instructions for accessing *DTASelect* filter files

The summary data tables that support this paper can be found in the present document. In addition to what is contained here, for those interested in SEQUEST scores and other analytical detail for individual peptides grouped by *P. gingivalis* ORF number, the *DTASelect* Ver. 1.9 filter files that contain this information in plain text format are posted at the URL below. This information will be primarily of interest to specialists in proteomics with the background to interpret the scores and filter parameters. In contrast, the summary data tables in this file are intended primarily for the oral biology and wider microbiology communities and thus only list information of interest to those groups. Microbiologists with specific questions regarding the interpretation of any particular portion of the proteomics data or data quality issues should contact Murray Hackett at *mhackett@u.washington.edu*.

http://depts.washington.edu/mhlab/

login name: guest

password: PGSGFNDATA

Please note that the password is case sensitive. No established public proteomic data archive at the time of submission was able to easily accommodate the scale of the raw mass spectrometry data acquired for this study, and the University of Washington does not at this time allow use of the TRANCHE system with university owned equipment. We hope this situation will be corrected in the future. Thus, the raw mass spectral data will be maintained on the corresponding author's server until such time as the mechanics of long-term archival storage can be arranged.

Notes for all tables

The W83 ORF numbers (PG numbers) and functional descriptors are primarily those derived from the work of K. E. Nelson *et al.* (2003) *J. Bacteriol.*, **185**:5591-5601 with some changes and corrections. The specific release of the annotation that served as the basis of our *P. gingivalis* ORF database is Ver. 3.1. For the latest information on the *P. gingivalis* genome sequence for W83, see *http://cmr.jcvi.org/cgi-bin/CMR/GenomePage.cgi?org=gpg*. For the PGN numbers derived from the annotation for strain ATCC 33277, see http://www.cb.k.u-tokyo.ac.jp/hattorilab/en/projects and M. Naito *et al.* (2008) *DNA Res.* **15**: 215-225. Table ST1 contains summary proteome information for the comparison of *P. gingivalis* incubated in the presence of *S. gordonii* and *F. nucleatum* with a control population of *P. gingivalis* incubated by itself under the same culture conditions as described in the main text. Table ST2 consists of qualitative protein detection information for secreted proteins observed under the same two conditions, with information about raw spectral counts and raw summed intensities, but no attempt has been made to draw quantitative conclusions for the secreted proteins, as

we did for the cellular proteins in Table ST1. The secreted protein preparations were reproducible in a qualitative sense, but not quantitatively. The isolation procedure for the secreted proteins described in the main text also extracted some surface bound proteins and cellular proteins as well.

Notes for Supplemental Table ST1

Protein Spectral Count fields

***q*-value:** this is a measure of false discovery rate that is discussed in the main text. In this study any q-value less than 0.01 was judged to be significant. Similarly to a p-value, the q-value is influenced by both the magnitude of the abundance change and the variance, but it is a measure of significance in terms of false discovery rate rather than false positive rate. It implicitly contains a correction for multiple hypothesis testing. See Storey and Tibshirani (2003) *PNAS* 100, 9440-9445 for a complete description of the q-value. As described in the main text, the spectral count q-value was derived from the uncorrected p-value associated with a G-test as described for proteomics data in Xia *et al.* (2007) *Inter. Jour. Mass Spectrom. 259*, 105-116. The actual value of G used for this work (see Tables ST1-ST3) was calculated globally as G_{Total} according to the method described in Sokal and Rohlf, *Biometry*, 3rd Ed. (1995), which takes advantage of the additivity of G for each individual comparison, e.g. $G_{Total} = G\,(PgSgFn\ I, Pg\ I) + G\,(PgSgFn\ I, Pg\ II) + G\,(PgSgFn\ II, Pg\ I) + G\,(PgSgFn\ II, Pg\ II)$ with 2 to 4 degrees of freedom, depending on missing values for certain ORFs.

Each individual value of G was calculated as given below, using the *PgSgFn / Pg* comparison as an example:

$$G = 2\left[f_{PgSgFn} \cdot \ln\left(\frac{f_{PgSgFn}}{\frac{f_{PgSgFn} + f_{Pg}}{2}} \right) + f_{Pg} \cdot \ln\left(\frac{f_{Pg}}{\frac{f_{PgSgFn} + f_{Pg}}{2}} \right) \right]$$

Where the expectation value, shown above as $\frac{f_{PgSgFn} + f_{Pg}}{2}$, of equal spectral counts for *PgSgFn* and *Pg* assumes a null hypothesis of zero abundance change between them using normalized data in which the total spectral counts have been made equal for each variable. The value of G_{Total} was then compared using R to the value of Chi-square at the appropriate degrees of freedom to derive the *p*-value, which, in turn, served as the input into the QVALUE R package. The correction for multiple hypothesis testing contained in the *q*-value is significantly less conservative in practice than other, more common, corrections to the *p*-value.

Pg: the average linear sum over both biological replicates of normalized spectral counts associated with a particular ORF for *P. gingivalis* incubated by itself.

PgSgFn: the average linear sum over both biological replicates of normalized spectral counts associated with a particular ORF for *P. gingivalis* incubated with *S. Gordonii* and *F. nucleatum.*

$\log_2$ Ratio: this is the global $\log_2$ ratio of the average of the normalized total spectral count values, e.g. $\log_2 [(PgSgFn\ I + PgSgFn\ II) \div (Pg\ I + Pg\ II)]$.

$\log_2$ Sum: the sum of the normalized global spectral counts for both conditions being compared. This number is primarily useful for estimating the degree to which a particular ORF is represented in the dataset. The reader is cautioned against making any assumptions that these numbers can be easily related to an absolute quantity of protein.

Regulation (abundance change fields for spectral counts and intensity)

The proteomic relative abundance color codes are **green**, an abundance ratio significantly less than zero on a $\log_2$ scale at a *q*-value cut-off of 0.01; **red**, an abundance ratio significantly greater than zero on a $\log_2$ scale at a *q*-value cut-off of 0.01; **yellow**, detected qualitatively but lacking statistical support for abundance change; **black**, no data for this ORF, a qualitative non-detect. The left hand dot codes the trend as determined using spectral counting. The right hand dot codes the trend as determined by summed signal intensity.

Summed Protein Intensity fields

***q*-value:** See the note for *q*-value given above. As described in the main text, the summed intensity *q*-value was derived from the uncorrected *p*-value associated with a paired *t*-test for the global score over n = 4 summed signal intensity comparisons. This was calculated according to the paired comparison method described in Sokal and Rohlf (1995), pages 352-356. The equation for global *t* is given below (n = 4), where *sd* is standard deviation, using the PgSgFn/Pg comparison as an example:

$$t = \frac{\{(PgSgFnI - PgI) + (PgSgFnI - PgII) + (PgSgFnII - PgI) + (PgSgFnII - PgII)\} / n}{sd / \sqrt{n}}$$

The value of t was calculated in R to derive the p-value, which in turn served as the input into the QVALUE R package as described in the experimental section of the main text.

$\log_2$ Ratio: this is the global $\log_2$ ratio of the average of the normalized total summed intensity values for *P. gingivalis* proteins under the two incubation conditions being compared.

Pg: the average linear sum over both biological replicates of normalized summed signal intensity for all unique peptides associated with a particular ORF for *P. gingivalis* incubated by itself.

PgSgFn: the average linear sum over both biological replicates of normalized summed signal intensity for all unique peptides associated with a particular ORF for *P. gingivalis* incubated with *S. Gordonii* and *F. nucleatum.*

A note regarding sampling depth and missing data

By sampling depth we refer to the degree that all possible peptides for all possible proteins are measured as mass spectral data that yields an interpretable result in terms of protein identification at frequencies which are representative of relative abundance and also reproducible. This is not an achievable goal with present technology in any absolute sense, but the better the sampling, the more useful and quantitative the dataset. Using the methods of this paper, the higher the number of redundant identifications (spectral counts for unique peptides that only map to one ORF), the more reliable the abundance ratios are likely to be. The work reported here was problematic for us in that the depth of sampling was such that we could not always report abundance changes as significant in the presence of q-values ≤ 0.01, due to missing data in either the numerator or denominator. In order avoid a divide by zero condition, our software substitutes a reasonable baseline value of spectral counts or intensity, based on data where we had ample objective evidence to support a depth of sampling that was good enough to assume that the absence of data in one channel was a reflection of biological reality rather than sampling error. Such assumptions were not valid for this particular study.

Assumptions regarding sampling depth can be evaluated by several methods, but two that we find especially useful are a frequency histogram of the entire set of $\log_2$ transformed abundance ratios collected for a given comparison and pseudo M versus A plots of the same ratios versus total spectral counts. Such histograms will often show satellite distributions that can indicate an inappropriate use of artificial baselines (see Figure SF5), normalization problems, or both. Inadequate sampling will lead to an irregular appearance of the histogram. In general such data appears as log-normally distributed with few irregularities when things have been done properly, even in cases where many proteins are changing (see Figure SF6 for a typical example). The choice of bin size can

also impact interpretation dramatically, and smaller bin sizes tend to be more informative. The pseudo M versus A plot can communicate much of the same information, but also shows the sampling depth relationship more explicitly on the x-axis (Fig. 2 in the main text). The combined effects of sequence overlaps among the different organisms and data acquisition speed limitations with the LTQ mass spectrometer were such that not as many unique peptides were observed for *P. gingivalis* in the presence of the other microbes as for *P. gingivalis* by itself or in the presence of human or bovine proteins. This all makes perfect sense given the large number of slightly overlapping inferred protein sequences shared among *P. gingivalis*, *S. gordonii* and *F. nucleatum*, and the minimal sequence overlap of *P. gingivalis* proteins with those of bovine or human origin. Because the quantitation is strongly influenced by the number of unique peptides recovered, the relative abundance calculations contained somewhat more scatter than what is normally observed with our single microbe studies. For the work reported here, we only retained abundance ratios for proteins with parent ion MS^1 data confirmed by high scoring linked MS^2 product ion data in both channels, regardless of *q*-value. In other words, a number of proteins initially called significant, based on assumptions encoded in software for *P. gingivalis* alone, were edited out by hand based on sampling assumptions more appropriate for *P. gingivalis* incubated with these other organisms.

Notes for additional figures SF1-SF6

Figures SF1-SF4 have been referred to in our past work as a reconstructed array images and represent a computer generated global overview of the *P. gingivalis* proteome. Four such overviews are given. Each image follows the same PGN ORF order and color coding for detected proteins as the Summary Data Table ST1, ascending PGN numbers (Ver. 1.0 of the ATTC 33277 annotation) from left to right, top to bottom. Figures SF1-SF2 contain quantitative summaries for the cellular data. Figures SF3-SF4 contain qualitative information regarding protein identification for secreted proteins, which are coded as orange for qualitative detection only. In all genome view plots gray indicates a non-detect. Black indicates an ORF number that is not used according to Ver. 3.1 of the W83 annotation, the basis for the original database used to search the raw data. If the gene was not assigned for W83, no PGN number would show up in this analysis. Thus a number of ORF assignaments that are unique to either the Japanese or LANL annotation of the ATCC 33277 genome sequence do not show up on these plots. Figure SF5 is a frequency histogram that illustrates the effect of improper use of artificial baselines with data of inadequate sampling depth. Figure SF6 illustrates the appearance of the same data after abundance ratios with artificial baselines in one channel have been removed. Figure SF6 is representative of the quantitative data reported in Table ST1, in that all inappropriately baselined data have been coded yellow for “no abundance change”.

TIGR / *PGN*	PgSgFn / Pg Spectral Count q-value	PgSgFn	Pg	log_2Ratio	log_2Sum	Regulation		Protein Intensity Regulation	PgSgFn / Pg Protein Intensity q-value	log_2Ratio	PgSgFn	Pg
PG0002	0.604563	2.13	4	-0.91	2.62	Y		Y	0.052108	-2.10	66413	1523645.5
PGN0002	*hexapeptide transferase family protein*											
PG0004	0.779998	17.06	17.5	-0.04	5.11	Y		Y	0.163917	-0.03	9614415	10074922.7
PGN0004	*transcriptional regulator Sir2 family*											
PG0005	0.507925	6.4	10	-0.64	4.04	Y		Y	0.085610	-2.64	1755618.5	3030023.63
PGN0005	*conserved hypothetical protein*											
PG0010	0.786190	21.32	22	-0.05	5.44	Y		G	0.007211	-0.85	4816840.5	8756436.3
PGN0008	*clpC ATP-dependent Clp protease ATP-binding subunit ClpC*											
PG0011	0.000488	36.25	68.5	-0.92	6.71	G		Y	0.025232	-0.57	21893500.5	31429682.3
PGN0009	*glycosyl hydrolase family 3*											
PG0012	0.061349	127.94	165	-0.37	8.19	Y		G	0.002890	-0.50	96560252.5	136059483
PGN0010	*L-threonine-O-3-phosphate decarboxylase putative*											
PG0016	0.543202	2.13	9	-2.08	3.48	Y		Y	0.011171	-5.67	66413	3656980.23
PGN0012	*sigma-54 dependent DNA-binding response regulator*											
PG0017	0.751373	2.13	3	-0.49	2.36	Y		Y	0.052108	-2.11	66413	2026410.5
PGN0013	*sensor histidine kinase*											
PG0020	0.751373	2.13	7	-1.72	3.19	Y		Y	0.017556	-4.10	66413	1292664.75
PGN0015	*transcriptional regulator MarR family*											
PG0021	0.779998	2.13	6	-1.49	3.02	Y		G	0.001540	-4.65	66413	1659629.85
PGN0016	*TIM-barrel protein putative NifR3 family*											
PG0022	0.000000	17.06	59.5	-1.8	6.26	G		Y	0.155528	0.19	13050763	11905689.8
PGN0017	*sulfate permease family protein*											
PG0024	0.773021	2.13	6.5	-1.61	3.11	Y		G	0.009001	-5.81	66413	3935942.93
PGN0020	*DNA-binding protein putative*											
PG0025	0.608518	8.53	10	-0.23	4.21	Y		Y	0.115558	-1.57	3975359.5	2361695.45
PGN0021	*fumarylacetoacetate hydrolase family protein*											
PG0026	0.715494	43.71	38	0.2	6.35	Y		G	0.000803	-0.26	17740142.5	21214334.2
PGN0022	*hypothetical protein PG_0026*											
PG0027	0.004096	242.02	298.5	-0.3	9.08	G		Y	0.020525	-0.61	150443576	221609242
PGN0023	*hypothetical protein PG_0027*											
PG0028	0.010359	2.13	16.5	-2.95	4.22	Y		G	0.004191	-6.14	66413	4746575.48
PGN0024	*ispF 2C-methyl-D-erythritol 24-cyclodiphosphate synthase*											
PG0030	0.024940	9.6	20.5	-1.09	4.91	Y		Y	0.014416	-4.25	3884213.5	12709376
PGN0026	*cytidine deaminase*											
PG0031	0.004405	20.26	42.5	-1.07	5.97	G		G	0.005972	-1.83	8773194	28743644.4
PGN0029	*hypothetical protein PG_0031*											
PG0032	0.004084	2.13	17.5	-3.04	4.29	Y		G	0.000595	-6.50	66413	5955795.8
PGN0030	*beta-mannosidase putative*											
PG0033	0.779998	2.13	6	-1.49	3.02	Y		G	0.001527	-5.82	66413	3756167.5
PGN0031	*RmuC domain protein*											
PG0034	0.720026	192.98	177.5	0.12	8.53	Y		Y	0.052108	-0.27	207076070	249862071
PGN0033	*trx thioredoxin*											
PG0035	0.683204	20.26	26	-0.36	5.53	Y		Y	0.032546	-0.33	7195544	8905958.5
PGN0034	*dnaE DNA polymerase III alpha subunit*											
PG0037	0.000000	115.15	51.5	1.16	7.38	R		R	0.005870	0.60	82127806.5	54875124.3
PGN0035	*rplS ribosomal protein L19*											
PG0042	0.000000	651.44	810.5	-0.32	10.51	G		G	0.003852	-0.36	857144404	1.1001e+09
PGN0038	*glyA serine hydroxymethyltransferase*											

TIGR	PGN	Description	Spectral Count q-value	Spectral Count PgSgFn	Spectral Count Pg	Spectral Count log_2Ratio	Spectral Count log_2Sum	Regulation (Spectral Count)	Regulation (Protein Intensity)	Protein Intensity q-value	Protein Intensity log_2Ratio	Protein Intensity PgSgFn	Protein Intensity Pg
PG0043	PGN0039	nahA beta-hexosaminidase	0.001408	77.83	116.5	-0.58	7.6	G	Y	0.015889	-0.50	44401008.5	62338800.6
PG0045	PGN0041	htpG heat shock protein HtpG	0.786857	42.65	45	-0.08	6.45	Y	G	0.004652	-0.33	21975084.5	27633555.7
PG0047	PGN0043	cell division protein FtsH putative	0.784897	67.17	63	0.09	7.02	Y	Y	0.053020	-0.30	28225229.5	33874089.8
PG0048	PGN0044	conserved hypothetical protein TIGR00092	0.000510	47.98	22.5	1.09	6.14	R	Y	0.021356	1.60	20958090.5	6535085.75
PG0831	PGN0072	hypothetical protein PG_0831	0.000077	10.66	1	3.41	3.54	Y	Y	0.051674	4.61	2783681.5	23108.5
PG2189	PGN0096	lysC aspartate kinase	0.083593	10.66	10	0.09	4.37	Y	Y	0.166018	-2.29	11998645.5	9981249.88
PG2190	PGN0097	ftsE cell-division ATP-binding protein	0.000252	18.13	34	-0.91	5.7	G	Y	0.080059	-3.25	5098917	8471093.95
PG2182	PGN0114	nqrA NADH:ubiquinone oxidoreductase Na translocating A subunit	0.630045	176.99	161	0.14	8.4	Y	Y	0.050907	-0.34	168807234	217211175
PG2181	PGN0115	nqrB NADH:ubiquinone oxidoreductase Na translocating B subunit	0.171691	7.46	15.5	-1.06	4.52	Y	G	0.002177	-4.82	1530725	11698131
PG2180	PGN0116	nqrC NADH:ubiquinone oxidoreductase Na translocating C subunit	0.008147	51.18	29.5	0.79	6.33	R	Y	0.124354	0.09	30757578	28166012.2
PG2177	PGN0119	nqrF NADH:ubiquinone oxidoreductase Na translocating F subunit	0.706763	102.35	98	0.06	7.65	Y	Y	0.027753	-0.29	75576879	92917037.2
PG2175	PGN0120	conserved hypothetical protein	0.009911	69.3	41.5	0.74	6.79	R	Y	0.019902	1.42	41869166	14691791.6
PG2174	PGN0121	hypothetical protein PG_2174	0.270049	141.8	162	-0.19	8.25	Y	Y	0.022021	-0.16	106292485	118610528
PG2173	PGN0122	omp28 outer membrane lipoprotein Omp28	0.669653	29.85	38	-0.35	6.08	Y	Y	0.011504	-0.64	29512392.5	46396646.4
PG2172	PGN0123	hypothetical protein PG_2172	0.400806	2.13	5	-1.23	2.83	Y	Y	0.052147	-1.65	66413	828252.2
PG2171	PGN0125	D-isomer specific 2-hydroxyacid dehydrogenase family protein	0.293852	156.73	180	-0.2	8.4	Y	G	0.003146	-0.58	55894128.5	83529258.6
PG2170	PGN0126	sugar transporter	0.604563	2.13	4	-0.91	2.62	Y	Y	0.053384	-1.37	66413	564566
PG2167	PGN0128	immunoreactive 53 kDa antigen PG123	0.000000	704.75	925	-0.39	10.67	G	Y	0.055854	-0.15	1.0017e+09	1.1184e+09
PG2168	PGN0129	hypothetical protein PG_2168	0.119599	149.27	158	-0.08	8.26	Y	Y	0.121255	0.12	106451395	95318163.5
PG2080	PGN0133	bioA adenosylmethionine--8-amino-7-oxononanoate aminotransferase	0.001904	2.13	18.5	-3.12	4.37	Y	G	0.009010	-6.38	66413	5875582.6
PG2082	PGN0135	POT family protein	0.010673	66.1	96	-0.54	7.34	Y	Y	0.025368	-0.55	80178741	117876921
PG2083	PGN0136	hypothetical protein PG_2083	0.594340	50.11	62.5	-0.32	6.82	Y	Y	0.017665	-0.68	27847694	45467340.4
PG2085	PGN0137	trpS tryptophanyl-tRNA synthetase	0.468913	69.3	87	-0.33	7.29	Y	Y	0.107706	0.09	58946932	55199385.4
PG2086	PGN0138	hypothetical protein PG_2086	0.707112	94.89	94.5	0.01	7.57	Y	Y	0.052108	0.63	106365874	73540363.3

TIGR / PGN	PgSgFn / Pg Spectral Count					Regulation		PgSgFn / Pg Protein Intensity			
	q-value	PgSgFn	Pg	log_2Ratio	log_2Sum			*q*-value	log_2Ratio	PgSgFn	Pg
PG2088	0.000000	10.66	46.5	-2.13	5.84	G	G	0.005100	-4.76	1068218	10963121.1
PGN0140	*msrA peptide methionine sulfoxide reductase*										
PG2089	0.201724	2.13	11.5	-2.43	3.77	Y	Y	0.012198	-6.77	66413	7936332.53
PGN0141	*hypothetical protein PG_2089*										
PG2090	0.604563	2.13	4	-0.91	2.62	Y	Y	0.052108	-1.84	66413	1071324.25
PGN0142	*cation efflux family protein*										
PG2091	0.549619	2.13	9	-2.08	3.48	Y	G	0.000019	-5.32	66413	2620105.1
PGN0143	*folB dihydroneopterin aldolase*										
PG2092	0.751373	35.18	37.5	-0.09	6.18	Y	Y	0.170519	0.02	14822955	14651116.2
PGN0144	*hypothetical protein PG_2092*										
PG2094	0.244618	2.13	11	-2.37	3.71	Y	Y	0.012550	-5.52	66413	3333112.5
PGN0145	*conserved domain protein*										
PG2095	0.543202	2.13	9	-2.08	3.48	Y	G	0.000962	-6.01	66413	4261250.1
PGN0147	*lipoprotein putative*										
PG2096	0.384046	10.66	9	0.24	4.3	Y	Y	0.020083	-4.10	530557	5422906.7
PGN0148	*conserved domain protein*										
PG2097	0.000003	381.7	275	0.47	9.36	R	Y	0.103945	-0.07	155644184	163521457
PGN0149	*prsA ribose-phosphate pyrophosphokinase*										
PG2099	0.015701	2.13	15	-2.82	4.1	Y	G	0.007002	-5.84	66413	3958634.43
PGN0150	*ATP-dependent RNA helicase DEAD/DEAH box family*										
PG2102	0.766366	60.77	56.5	0.11	6.87	Y	Y	0.035573	0.39	22014089	16604386.4
PGN0152	*immunoreactive 61 kDa antigen PG91*										
PG2105	0.000074	2.13	22.5	-3.4	4.62	Y	G	0.004947	-7.21	66413	10095562.5
PGN0154	*lipoprotein putative*										
PG2106	0.000000	188.72	428.5	-1.18	9.27	G	G	0.009741	-0.63	81577162	123821836
PGN0156	*hypothetical protein PG_2106*										
PG2107	0.000000	50.11	105	-1.07	7.28	G	G	0.003146	-0.61	25205097	38302278.6
PGN0157	*thiH thiH protein*										
PG2108	0.703738	57.57	68.5	-0.25	6.98	Y	Y	0.016480	-0.49	57017455.5	80519945
PGN0158	*thiG thiG protein*										
PG2109	0.000000	8.53	38	-2.16	5.54	G	G	0.003160	-5.29	2219073.5	19740976.6
PGN0159	*hydroxymethylpyrimidine kinase/thiamin-phosphate pyrophosphorylase*										
PG2110	0.283063	147.13	170	-0.21	8.31	Y	G	0.002116	-0.41	104270922	138720721
PGN0160	*thiC thiamine biosynthesis protein ThiC*										
PG2117	0.001293	95.96	56.5	0.76	7.25	R	R	0.003861	0.99	117098170	58794274.8
PGN0167	*rpsP ribosomal protein S16*										
PG2120	0.720026	2.13	7.5	-1.82	3.27	Y	G	0.002832	-5.47	66413	2963131.35
PGN0169	*metallo-beta-lactamase superfamily protein*										
PG2121	0.751373	41.58	34.5	0.27	6.25	Y	Y	0.010514	-0.18	22274929.5	25282149.2
PGN0170	*ansA L-asparaginase*										
PG2124	0.000000	2744.37	3259.5	-0.25	12.55	G	Y	0.052108	-0.24	1.5778e+09	1.8331e+09
PGN0173	*gapA glyceraldehyde 3-phosphate dehydrogenase type I*										
PG2125	0.001057	8.53	1	3.09	3.25	Y	Y	0.046188	3.20	418644.5	23108.5
PGN0174	*transcriptional regulator AraC family*										
PG2126	0.034356	28.79	44.5	-0.63	6.2	Y	Y	0.028575	-1.13	3455277	7464610.05
PGN0175	*conserved hypothetical protein TIGR00044*										
PG2127	0.023824	35.18	59.5	-0.76	6.56	Y	Y	0.032387	-0.32	19956343.5	25193696.4
PGN0176	*hypothetical protein PG_2127*										

TIGR	PGN	Description	PgSgFn / Pg Spectral Count q-value	PgSgFn	Pg	log_2Ratio	log_2Sum	Regulation (Spectral Count)	Regulation (Protein Intensity)	PgSgFn / Pg Protein Intensity q-value	log_2Ratio	PgSgFn	Pg
PG2130	PGN0178	hypothetical protein PG_2130	0.751373	23.46	20.5	0.19	5.46	Y	Y	0.014264	-0.84	8826782.5	16018503.7
PG2131	PGN0179	60 kDa protein	0.029238	219.63	268.5	-0.29	8.93	Y	G	0.000161	-0.27	164682512	198569303
P13793IFMA_PORGI	PGN0180	major fimbrillin A, Porphyromonas gingivalis 33277	0.000000	1954.32	3042	-0.64	12.29	G	Y	0.073682	-0.22	954032009	1.0887e+09
PG2134	PGN0183	lipoprotein putative	0.004290	10.66	26.5	-1.31	5.22	G	Y	0.037727	-3.89	6048720.5	14645875.9
PG2139	PGN0187	hypothetical protein PG_2139	0.468582	2.13	9	-2.08	3.48	Y	Y	0.035857	-3.96	66413	1554353.7
PG2140	PGN0188	rpmF ribosomal protein L32	0.594734	20.26	13	0.64	5.06	Y	Y	0.063017	0.14	3439388	3140245.35
PG2141	PGN0189	fabH 3-oxoacyl-(acyl-carrier-protein) synthase III	0.000252	122.61	183	-0.58	8.26	G	G	0.006530	-0.93	71402955	137293027
PG2142	PGN0190	era GTP-binding protein Era	0.250677	10.66	15.5	-0.54	4.71	Y	Y	0.091569	-3.28	5901502.5	9190348.93
PG2143	PGN0191	GTP-binding protein Era/ThdF family	0.056869	25.59	41	-0.68	6.06	Y	G	0.005259	-0.53	11105769	15942996.1
PG2144	PGN0192	hypothetical protein PG_2144	0.621265	61.84	73.5	-0.25	7.08	Y	G	0.006605	-0.24	35258539	41675895.6
PG2145	PGN0193	polysaccharide deacetylase	0.751373	2.13	7	-1.72	3.19	Y	Y	0.014264	-4.82	66413	2074907.5
PG2147	PGN0195	xpt xanthine phosphoribosyltransferase	0.667335	2.13	8	-1.91	3.34	Y	G	0.000766	-5.52	66413	3019948.1
PG2149	PGN0197	hypothetical protein PG_2149	0.423387	2.13	9.5	-2.16	3.54	Y	Y	0.018791	-6.15	66413	5537261.98
PG2150	PGN0198	LysM domain protein	0.000046	6.4	30	-2.23	5.19	G	G	0.007211	-4.39	2675015.5	11497716.6
PG2155	PGN0200	lipoprotein putative	0.751373	59.71	51	0.23	6.79	Y	Y	0.146675	0.08	37096520	35447450.7
PG2156	PGN0201	conserved hypothetical protein TIGR00046	0.667335	2.13	8	-1.91	3.34	Y	Y	0.043161	-6.63	66413	13317526
PG2157	PGN0202	glutamine cyclotransferase-related protein	0.777944	54.38	60	-0.14	6.84	Y	G	0.001987	-0.49	32933674.5	46184709
PG2158	PGN0203	conserved hypothetical protein	0.091012	8.53	19	-1.16	4.78	Y	Y	0.015947	-3.80	3258761.5	9745822.43
PG2159	PGN0204	protoporphyrinogen oxidase	0.005139	2.13	17.5	-3.04	4.29	Y	G	0.000986	-6.29	66413	5169838.98
PG2162	PGN0206	lpxB lipid A disaccharide synthase	0.516095	7.46	12	-0.69	4.28	Y	Y	0.111797	-2.17	1512760.5	2150215.15
PG2163	PGN0207	surE stationary-phase survival protein SurE	0.008983	10.66	23.5	-1.14	5.09	G	Y	0.177260	-3.39	10638742	11510265
PG2164	PGN0208	peptidyl-prolyl cis-trans isomerase FKBP-type	0.037701	60.77	38	0.68	6.63	Y	Y	0.052108	0.16	36209961.5	32563934.2
PG2165	PGN0209	glyS glycyl-tRNA synthetase	0.648731	104.49	115.5	-0.14	7.78	Y	R	0.003126	0.37	107562538	83219095.8
PG2078	PGN0211	conserved hypothetical protein	0.000903	13.86	28.5	-1.04	5.4	G	Y	0.145853	-3.52	9637101	11996317.9

TIGR	PgSgFn / Pg Spectral Count							PgSgFn / Pg Protein Intensity			
PGN	*q*-value	PgSgFn	Pg	log_2Ratio	log_2Sum	Regulation		*q*-value	log_2Ratio	PgSgFn	Pg
PG0104	0.039368	2.13	14.5	-2.77	4.06	Y	G	0.001091	-6.21	66413	4878730.4
PGN0221	*topB-1 DNA topoisomerase III*										
PG0108	0.335331	70.37	87	-0.31	7.3	Y	Y	0.099350	-0.13	32022370.5	34745253.8
PGN0224	*epsD UDP-N-acetyl-D-mannosaminuronic acid dehydrogenase*										
PG0119	0.006508	39.45	20	0.98	5.89	R	Y	0.056585	-0.73	6494815.5	10444360.9
PGN0233	*glycosyl transferase WecB/TagA/CpsF family*										
PG0120	0.001477	71.43	112	-0.65	7.52	G	G	0.000584	-0.12	36447759	39739665.3
PGN0234	*epsC UDP-N-acetylglucosamine 2-epimerase*										
PG0121	0.000000	943.58	701	0.43	10.68	R	Y	0.147498	0.03	1.0492e+09	1.0177e+09
PGN0235	*hup-1 DNA-binding protein HU*										
PG0125	0.543202	2.13	9	-2.08	3.48	Y	Y	0.015386	-5.39	66413	3124207.1
PGN0238	*hypothetical protein PG_0125*										
PG0126	0.720026	26.65	20.5	0.38	5.56	Y	Y	0.103945	0.10	13191995.5	12401596.7
PGN0239	*type I phosphodiesterase/nucleotide pyrophosphatase family protein*										
PG0128	0.278503	2.13	10.5	-2.3	3.66	Y	G	0.000381	-6.26	66413	5032655.23
PGN0241	*conserved domain protein*										
PG0129	0.004789	2.13	17.5	-3.04	4.29	Y	G	0.003852	-7.16	66413	9641296.35
PGN0242	*mannosyltransferase*										
PG0130	0.000000	413.68	256.5	0.69	9.39	R	R	0.006502	0.31	341591951	276323574
PGN0243	*gpm phosphoglycerate mutase*										
PG0134	0.327141	17.06	27.5	-0.69	5.48	Y	G	0.003547	-0.66	5909573.5	9326663.45
PGN0247	*mgtE magnesium transporter*										
PG0135	0.671572	20.26	14	0.53	5.1	Y	Y	0.053939	-0.32	5951048.5	7292105.03
PGN0248	*ksgA dimethyladenosine transferase*										
PG0136	0.336169	2.13	10.5	-2.3	3.66	Y	G	0.006011	-5.67	66413	3486804
PGN0249	*hypothetical protein PG_0136*										
PG0137	0.006441	204.71	249	-0.28	8.83	G	G	0.001602	-0.53	132006149	190668333
PGN0250	*pepD-1 aminoacyl-histidine dipeptidase*										
PG0138	0.166597	317.72	276.5	0.2	9.21	Y	Y	0.064220	0.10	415543450	387398784
PGN0251	*fabD malonyl CoA-acyl carrier protein transacylase*										
PG0139	0.273687	2.13	11	-2.37	3.71	Y	G	0.000605	-5.75	66413	3535477.88
PGN0252	*membrane-bound lytic murein transglycosylase D putative*										
PG0141	0.031975	21.32	36.5	-0.78	5.85	Y	G	0.009529	-0.74	11360222.5	18824805
PGN0254	*spo0J spoOJ protein*										
PG0142	0.630045	2.13	8	-1.91	3.34	Y	G	0.003939	-6.63	66413	6666694.5
PGN0255	*soj SpoOJ regulator protein*										
PG0143	0.751373	20.26	24.5	-0.27	5.48	Y	Y	0.011148	-0.78	13754427.5	22810262
PGN0256	*hydrolase carbon-nitrogen family*										
PG0144	0.015701	34.12	58	-0.77	6.53	Y	G	0.001455	-1.31	10160389.5	25131094.3
PGN0257	*conserved hypothetical protein*										
PG0147	0.784897	2.13	2	0.09	2.05	Y	Y	0.052485	-1.57	66413	734684.75
PGN0260	*hypothetical protein PG_0147*										
PG0148	0.017364	8.53	21.5	-1.33	4.91	Y	Y	0.193910	-3.25	10634858	10626809.6
PGN0261	*sigma-54-dependent transcriptional regulator*										
PG0149	0.543202	2.13	9	-2.08	3.48	Y	G	0.009241	-5.80	66413	3937145.9
PGN0262	*conserved domain protein*										
PG0150	0.083593	10.66	9	0.24	4.3	Y	Y	0.177260	-2.11	2517021	2318493.83
PGN0263	*conserved hypothetical protein TIGR01125*										

TIGR	PgSgFn / Pg Spectral Count						PgSgFn / Pg Protein Intensity				
PGN	*q*-value	PgSgFn	Pg	log_2Ratio	log_2Sum	Regulation		*q*-value	log_2Ratio	PgSgFn	Pg
PG0151	0.000813	39.45	68.5	-0.8	6.75	G	Y	0.027198	-0.66	11724111	18491458.9
PGN0264	*ftsY signal recognition particle-docking protein FtsY*										
PG0152	0.004364	2.13	17.5	-3.04	4.29	Y	G	0.000238	-6.59	66413	6332466.53
PGN0265	*nspC carboxynorspermidine decarboxylase*										
PG0153	0.784897	206.84	196.5	0.07	8.66	Y	Y	0.166018	0.01	160413108	159528651
PGN0266	*aspS aspartyl-tRNA synthetase*										
PG0159	0.000000	993.69	1334	-0.42	11.18	G	Y	0.055138	-0.10	752395255	807987810
PGN0271	*pepO endopeptidase PepO*										
PG0160	0.560362	126.88	126.5	0	7.99	Y	Y	0.071070	0.39	85075030.5	63745050.2
PGN0272	*conserved domain protein*										
PG0163	0.786859	27.72	29	-0.07	5.83	Y	Y	0.022021	0.29	13621052.5	11199275.3
PGN0275	*pfk phosphofructokinase*										
PG0166	0.779998	2.13	2.5	-0.23	2.21	Y	Y	0.052108	-2.04	66413	1832715
PGN0278	*pth peptidyl-tRNA hydrolase*										
PG0167	0.337844	224.97	201.5	0.16	8.74	Y	Y	0.183324	-0.00	290682261	287752958
PGN0279	*rplY ribosomal protein L25*										
PG0170	0.035356	277.21	228	0.28	8.98	Y	G	0.003547	-0.08	157839514	166932099
PGN0281	*metG methionyl-tRNA synthetase*										
PG0171	0.648731	24.52	27	-0.14	5.69	Y	Y	0.179184	0.11	9248078.5	9024560.3
PGN0282	*5'-nucleotidase family protein*										
PG0174	0.267463	9.6	15.5	-0.69	4.65	Y	Y	0.017881	-3.27	1726161	4899686.63
PGN0286	*pyridine nucleotide-disulphide oxidoreductase family protein*										
PG0179	0.098759	200.44	240	-0.26	8.78	Y	Y	0.147498	0.07	150218239	144809297
PGN0288	*hypothetical protein PG_0179*										
PG0180	0.003180	74.63	111.5	-0.58	7.54	G	G	0.006461	-0.80	47581413.5	82171950.4
PGN0289	*lipoprotein putative*										
PG0181	0.648731	138.6	121.5	0.19	8.02	Y	Y	0.121255	-0.04	108729237	111945438
PGN0290	*immunoreactive 32 kDa antigen PG49*										
PG0182	0.728841	186.58	198	-0.09	8.59	Y	G	0.000238	-0.41	99981015.5	132616804
PGN0291	*von Willebrand factor type A domain protein*										
PG0185	0.000000	1643	1329.5	0.31	11.54	R	Y	0.011171	-0.18	1.1843e+09	1.3433e+09
PGN0293	*ragA ragA protein*										
PG0186	0.262411	10.66	15	-0.49	4.68	Y	Y	0.010409	-1.46	108537.5	291160.05
PGN0294	*ragB lipoprotein RagB*										
PG0188	0.012680	50.11	79.5	-0.67	7.02	Y	Y	0.020985	-1.42	25298628	68515843.8
PGN0296	*lipoprotein putative*										
PG0190	0.028913	12.79	18	-0.49	4.94	Y	Y	0.028659	-4.09	3409252	11492669.7
PGN0298	*uppS undecaprenyl diphosphate synthase*										
PG0191	0.659176	117.28	108.5	0.11	7.82	Y	Y	0.052147	-0.26	77872724	95190563.8
PGN0299	*outer membrane protein putative*										
PG0192	0.000000	379.56	232.5	0.71	9.26	R	R	0.003925	0.24	191541166	162438751
PGN0300	*ompH-1 cationic outer membrane protein OmpH*										
PG0193	0.009141	190.85	145	0.4	8.39	R	R	0.004348	0.49	241036566	171034153
PGN0301	*ompH-2 cationic outer membrane protein OmpH*										
PG0195	0.000000	33.05	129	-1.96	7.34	G	G	0.000137	-1.61	17436723	53034778.5
PGN0302	*rubrerythrin*										
PG0196	0.001057	933.98	845	0.14	10.8	R	Y	0.035292	0.19	719917664	633712229
PGN0303	*peptidase M16 family*										

TIGR	PgSgFn / Pg Spectral Count						PgSgFn / Pg Protein Intensity				
PGN	*q*-value	PgSgFn	Pg	log_2Ratio	log_2Sum	Regulation		*q*-value	log_2Ratio	PgSgFn	Pg
PG0198	0.660293	2.13	8	-1.91	3.34	Y	G	0.001694	-5.97	66413	4161931
PGN0306	*conserved hypothetical protein*										
PG0202	0.000000	8.53	52.5	-2.62	5.93	G	G	0.002140	-5.48	3153845	26644374.8
PGN0310	*uroporphyrinogen-III synthase HemD putative*										
PG0205	0.043660	26.65	46.5	-0.8	6.19	Y	Y	0.048991	-0.59	9906366	13900846.7
PGN0313	*prfC peptide chain release factor 3*										
PG0210	0.779998	2.13	6	-1.49	3.02	Y	Y	0.010656	-4.30	66413	1395692.5
PGN0315	*precorrin-6x reductase/cobalamin biosynthetic protein CbiD*										
PG0211	0.475857	2.13	9.5	-2.16	3.54	Y	G	0.009241	-6.18	66413	5114360.15
PGN0316	*cbiGF cobalamin biosynthesis protein CbiG/precorrin-4 C11-methyltransferase*										
PG0212	0.660293	2.13	8	-1.91	3.34	Y	G	0.001602	-5.43	66413	2867950.38
PGN0317	*cobL precorrin-6Y C515-methyltransferase decarboxylating*										
PG0213	0.000838	7.46	27	-1.86	5.11	G	Y	0.013141	-3.88	2605088	9324048.85
PGN0318	*precorrin-3 methylase/precorrin-8X methylmutase*										
PG0217	0.549877	21.32	30	-0.49	5.68	Y	Y	0.019165	-0.96	12452859.5	24877507.1
PGN0322	*hypothetical protein PG_0217*										
PG0218	0.720026	2.13	7.5	-1.82	3.27	Y	G	0.001242	-6.25	66413	5030806.5
PGN0323	*hypothetical protein PG_0218*										
PG0226	0.001268	476.59	387.5	0.3	9.76	R	G	0.007211	-0.25	220140867	261015848
PGN0329	*transglutaminase-related protein*										
PG0227	0.001293	2.13	18	-3.08	4.33	Y	Y	0.022019	-5.33	66413	3234392.78
PGN0330	*radA DNA repair protein RadA*										
PG0228	0.707112	41.58	38.5	0.11	6.32	Y	Y	0.040771	0.21	26610621	22851811.2
PGN0331	*DdaH family protein*										
PG0230	0.013991	318.79	326.5	-0.03	9.33	Y	Y	0.179184	0.01	255084871	253439430
PGN0333	*transaldolase TalC putative*										
PG0231	0.216301	2.13	11.5	-2.43	3.77	Y	G	0.003885	-7.67	66413	13678978.1
PGN0334	*conserved hypothetical protein*										
PG0232	0.012680	632.25	593.5	0.09	10.26	Y	Y	0.167293	0.08	554581265	538007845
PGN0335	*zinc carboxypeptidase putative*										
PG0235	0.751373	2.13	3	-0.49	2.36	Y	Y	0.084882	-0.26	66413	130699
PGN0340	*carboxyl-terminal protease*										
PG0236	0.158167	8.53	14	-0.71	4.49	Y	Y	0.116286	-3.45	8861345	13840866.6
PGN0341	*hypothetical protein PG_0236*										
PG0237	0.560362	2.13	8.5	-2	3.41	Y	G	0.001850	-5.32	66413	2644811.33
PGN0342	*ung uracil-DNA glycosylase*										
PG0240	0.758093	2.13	6.5	-1.61	3.11	Y	Y	0.023101	-5.17	66413	2950794
PGN0344	*hydrolase haloacid dehalogenase-like family*										
PG0241	0.067161	122.61	93.5	0.39	7.76	Y	Y	0.014837	-0.29	171546139	210917196
PGN0345	*lipoprotein putative*										
PG0242	0.009824	2.13	16.5	-2.95	4.22	Y	G	0.003547	-6.55	66413	6272651.53
PGN0346	*conserved hypothetical protein TIGR00096*										
PG0243	0.784897	22.39	20	0.16	5.41	Y	Y	0.165042	0.04	12019403.5	11542324.8
PGN0347	*hypothetical protein PG_0243*										
PG0245	0.000001	518.17	389.5	0.41	9.83	R	Y	0.046400	-0.04	273102809	281601165
PGN0348	*universal stress protein family*										
PG0246	0.000000	47.98	124	-1.37	7.43	G	G	0.009208	-0.41	87558320	115662344
PGN0349	*hypothetical protein PG_0246*										

TIGR	PGN	Description	PgSgFn / Pg Spectral Count q-value	PgSgFn	Pg	log_2Ratio	log_2Sum	Regulation (Spectral Count)	Regulation (Protein Intensity)	PgSgFn / Pg Protein Intensity q-value	log_2Ratio	PgSgFn	Pg
PG0248	PGN0350	translation initation factor SUI1 putative	0.751373	2.13	3	-0.49	2.36	Y	Y	0.052108	-1.73	66413	1196860
PG0249	PGN0351	oxaloacetate decarboxylase putative	0.000393	431.81	346	0.32	9.6	R	Y	0.156645	0.01	365397275	361066398
PG0253	PGN0353	conserved hypothetical protein	0.659176	2.13	7.5	-1.82	3.27	Y	G	0.007211	-4.60	66413	1668510.35
PG0254	PGN0354	N utilization substance protein A putative	0.202549	275.08	311.5	-0.18	9.2	Y	G	0.000999	-0.27	125841294	152033740
PG0255	PGN0355	infB translation initiation factor IF-2	0.031300	280.41	224	0.32	8.98	Y	R	0.001020	0.45	171819430	125959024
PG0257	PGN0357	conserved hypothetical protein	0.372858	21.32	31	-0.54	5.71	Y	Y	0.016480	-1.03	5048640.5	9414955.73
PG0258	PGN0358	ABC transporter ATP-binding protein	0.520799	59.71	46	0.38	6.72	Y	R	0.007705	0.52	36477010	25554325
PG0259	PGN0359	conserved hypothetical protein	0.510938	20.26	29	-0.52	5.62	Y	Y	0.173433	0.09	19563312.5	19052965.9
PG0263	PGN0360	tyrS tyrosyl-tRNA synthetase	0.732133	169.52	159.5	0.09	8.36	Y	Y	0.031171	-0.35	56240026.5	70758985.9
PG0264	PGN0361	glycosyl transferase group 2 family protein	0.014598	2.13	16	-2.91	4.18	Y	G	0.001595	-5.69	66413	3424303.73
PG0267	PGN0365	argS arginyl-tRNA synthetase	0.595083	94.89	101.5	-0.1	7.62	Y	Y	0.179184	0.01	41036160	40803439.1
PG0268	PGN0366	trmU tRNA (5-methylaminomethyl-2-thiouridylate)-methyltransferase	0.475857	2.13	9.5	-2.16	3.54	Y	G	0.000770	-6.25	66413	5013729.9
PG0269	PGN0367	xth exodeoxyribonuclease III	0.000001	2.13	27.5	-3.69	4.89	Y	G	0.005239	-8.04	66413	17958640.9
PG0270	PGN0368	oxyR redox-sensitive transcriptional activator OxyR	0.773021	28.79	31.5	-0.13	5.91	Y	Y	0.017656	-0.32	13941564	17390804.5
PG0271	PGN0369	ssb single-stranded binding protein	0.007630	2.13	17	-3	4.26	Y	G	0.000107	-7.57	66413	12531717.7
PG0272	PGN0370	CBS domain protein	0.751373	2.13	3	-0.49	2.36	Y	Y	0.052108	-1.81	66413	1340150
PG0275	PGN0373	thioredoxin family protein	0.339193	76.77	60	0.36	7.1	Y	Y	0.147498	-0.07	64157505.5	67072061.1
PG0276	PGN0374	conserved hypothetical protein	0.134654	72.5	50	0.54	6.94	Y	Y	0.169545	0.01	30110824	29392377.5
PG1745	PGN0375	phosphoribulokinase family protein	0.714916	56.51	58.5	-0.05	6.85	Y	Y	0.032184	-0.37	24199733	31228486.9
PG1743	PGN0376	kdsA 2-dehydro-3-deoxyphosphooctonate aldolase	0.782623	26.65	27.5	-0.05	5.76	Y	Y	0.033100	-0.16	13701612	15293716.5
PG1741	PGN0377	aspA aspartate ammonia-lyase	0.355429	770.85	736.5	0.07	10.56	Y	Y	0.010105	0.32	487679373	393148538
PG1739	PGN0378	conserved domain protein	0.751373	2.13	3	-0.49	2.36	Y	Y	0.053629	-1.23	66413	607109
PG1731	PGN0386	aroQ 3-dehydroquinate dehydratase type II	0.307024	2.13	10.5	-2.3	3.66	Y	G	0.001865	-6.25	66413	5052757.43
PG1729	PGN0388	thiol peroxidase	0.000001	505.37	432.5	0.22	9.87	R	Y	0.052108	-0.17	439342561	498508443

TIGR	PGN	Description	PgSgFn / Pg Spectral Count						PgSgFn / Pg Protein Intensity				
			q-value	PgSgFn	Pg	log_2Ratio	log_2Sum	Regulation	Regulation	*q*-value	log_2Ratio	PgSgFn	Pg
PG1728	PGN0389	*cytidine/deoxycytidylate deaminase family protein*	0.400806	2.13	5	-1.23	2.83	Y	Y	0.052108	-2.22	66413	1790631.4
PG1727	PGN0390	*yitL yitL protein*	0.751373	2.13	7	-1.72	3.19	Y	Y	0.022497	-4.40	66413	1699312.8
PG1726	PGN0391	*PDZ domain protein*	0.000330	57.57	100	-0.8	7.3	G	Y	0.022350	-0.75	33518448	57551224.7
PG1724	PGN0393	*gcp O-sialoglycoprotein endopeptidase*	0.285375	8.53	9.5	-0.16	4.17	Y	Y	0.046400	-2.98	1634421.5	3383904.6
PG1723	PGN0394	*rpsT ribosomal protein S20*	0.000000	70.37	25.5	1.46	6.58	R	R	0.003776	1.87	61475367	17022833.6
PG1721	PGN0396	*vacB ribonuclease R*	0.773021	65.04	57.5	0.18	6.94	Y	Y	0.052108	-0.13	35407828	38842936.7
PG1720	PGN0397	*conserved domain protein*	0.212556	12.79	15	-0.23	4.8	Y	Y	0.168269	-2.80	5951534.5	6708171.55
PG1719	PGN0398	*ABC transporter ATP-binding protein MsbA family*	0.033441	14.93	22	-0.56	5.21	Y	Y	0.150663	-2.88	5607170	6836670.45
PG1715	PGN0400	*hypothetical protein PG_1715*	0.543202	33.05	31	0.09	6	Y	Y	0.018304	-0.49	8765318	12080743.5
PG1714	PGN0403	*pdxH pyridoxamine-phosphate oxidase*	0.712239	65.04	54	0.27	6.9	Y	Y	0.062627	0.05	39514633.5	38107400.7
PG1712	PGN0405	*alpha-12-mannosidase family protein*	0.045519	14.93	20.5	-0.46	5.15	Y	Y	0.071676	-2.75	2259090.5	4735819.1
PG1711	PGN0406	*alpha-12-mannosidase family protein*	0.000681	9.6	28	-1.54	5.23	G	Y	0.041452	-3.91	4886292	11532217
PG1704	PGN0411	*thiol:disulfide interchange protein dsbD putative*	0.166597	159.93	128.5	0.32	8.17	Y	Y	0.104719	-0.07	120806535	126382261
PG1703	PGN0412	*MazG family protein*	0.175055	30.92	22	0.49	5.73	Y	Y	0.017556	-0.51	5925039	8383796.68
PG1702	PGN0413	*gyrB DNA gyrase B subunit*	0.000127	184.45	229.5	-0.32	8.69	G	Y	0.014936	-0.43	73716839	98285752.8
PG1701	PGN0414	*glutamine amidotransferase class II/dipeptidase*	0.370460	60.77	53.5	0.18	6.84	Y	Y	0.023219	-0.24	13387001	15699725.8
PG1697	PGN0415	*type II restriction endonuclease putative*	0.000037	24.52	55.5	-1.18	6.32	G	G	0.002913	-1.64	9109170	26521350.9
PG1696	PGN0416	*type II DNA modification methyltransferase putative*	0.608229	2.13	8.5	-2	3.41	Y	G	0.002196	-6.20	66413	4892160.9
PG1694	PGN0418	*conserved hypothetical protein*	0.507925	2.13	4.5	-1.08	2.73	Y	Y	0.052108	-1.88	66413	1121982.05
PG1693	PGN0419	*HesA/MoeB/ThiF family protein*	0.600049	2.13	4	-0.91	2.62	Y	Y	0.052108	-2.27	66413	2508020
PG1692	PGN0420	*ABC transporter ATP-binding protein*	0.000000	2.13	35.5	-4.06	5.23	Y	G	0.001742	-6.58	66413	6345381.9
PG1690	PGN0422	*Sua5/YciO/YrdC/YwlC family protein*	0.779998	2.13	6	-1.49	3.02	Y	G	0.001987	-5.76	66413	3607578.98
PG1688	PGN0423	*greA transcription elongation factor GreA*	0.648995	51.18	43	0.25	6.56	Y	Y	0.159862	-0.01	37705804	37936692.6
PG1687	PGN0424	*HIT family protein*	0.389823	6.4	11.5	-0.85	4.16	Y	Y	0.073058	-4.31	15318345.5	26371929.5

TIGR	PgSgFn / Pg Spectral Count						PgSgFn / Pg Protein Intensity				
PGN	*q*-value	PgSgFn	Pg	log_2Ratio	log_2Sum	Regulation		*q*-value	log_2Ratio	PgSgFn	Pg
PG1683	0.389823	34.12	25.5	0.42	5.9	Y	Y	0.015803	-0.62	7872712	11832455.9
PGN0427	*conserved hypothetical protein*										
PG1682	0.201724	2.13	11.5	-2.43	3.77	Y	G	0.002914	-6.77	66413	7286548.38
PGN0428	*glycosyl transferase group 1 family protein*										
PG1681	0.000000	10.66	44.5	-2.06	5.79	G	G	0.000770	-5.13	859710	12375252.9
PGN0429	*glycogen debranching enzyme archaeal type putative*										
PG1677	0.751373	282.54	284	-0.01	9.15	Y	G	0.003885	-0.23	194743987	228993217
PGN0433	*pgk phosphoglycerate kinase*										
PG1676	0.000000	2906.43	3222.5	-0.15	12.58	G	Y	0.077954	0.03	1.5752e+09	1.5422e+09
PGN0434	*pckA phosphoenolpyruvate carboxykinase (ATP)*										
PG1667	0.527466	51.18	39	0.39	6.49	Y	R	0.007211	0.28	24155810.5	19825684.6
PGN0444	*outer membrane efflux protein*										
PG1666	0.311506	15.99	25	-0.64	5.36	Y	Y	0.025723	0.21	8605191	7475370.78
PGN0445	*efflux transporter MFP component RND family*										
PG1665	0.751373	2.13	3	-0.49	2.36	Y	Y	0.052108	-1.93	66413	1210753.5
PGN0446	*ABC transporter permease protein putative*										
PG1663	0.751373	2.13	3	-0.49	2.36	Y	Y	0.052192	-1.50	66413	873910.5
PGN0448	*ABC transporter ATP-binding protein*										
PG1662	0.600049	2.13	8.5	-2	3.41	Y	G	0.000005	-6.30	66413	5160553
PGN0449	*hypothetical protein PG_1662*										
PG1657	0.055728	108.75	141.5	-0.38	7.97	Y	Y	0.012090	-0.37	76707025	99041373.6
PGN0456	*mutB methylmalonyl-CoA mutase large subunit*										
PG1656	0.012371	138.6	184.5	-0.41	8.34	Y	Y	0.057584	-0.31	93395525.5	113847141
PGN0457	*mutA methylmalonyl-CoA mutase small subunit*										
PG0222	0.784897	2.13	2	0.09	2.05	Y	Y	0.052108	-2.22	66413	2364117
PGN0460	*DNA-binding protein histone-like family*										
PG1653	0.002349	47.98	82	-0.77	7.02	G	Y	0.015803	-0.50	23853297.5	33146311.1
PGN0462	*conserved hypothetical protein*										
PG1652	0.005103	61.84	96	-0.63	7.3	G	G	0.001227	-1.10	33394118.5	70856455.9
PGN0463	*hypothetical protein PG_1652*										
PG1651	0.764248	107.69	104.5	0.04	7.73	Y	R	0.004649	0.12	87018923	80196972.6
PGN0464	*TPR domain protein*										
PG1648	0.000071	2.13	22.5	-3.4	4.62	Y	Y	0.016268	-7.13	66413	10611625.1
PGN0465	*RelA/SpoT family protein*										
PG1647	0.050334	2.13	14	-2.72	4.01	Y	G	0.007285	-4.35	66413	1409048.5
PGN0466	*cls cardiolipin synthetase*										
PG1619	0.751373	2.13	3	-0.49	2.36	Y	Y	0.056585	-1.00	66413	340454.75
PGN0469	*biotin synthesis protein BioC putative*										
PG1620	0.720026	2.13	7	-1.72	3.19	Y	G	0.004947	-5.59	66413	3266146.03
PGN0470	*carboxyl-terminal protease-related protein*										
PG1622	0.272530	23.46	36	-0.62	5.89	Y	Y	0.126610	-0.66	15635105.5	19636174.5
PGN0472	*DNA topoisomerase IV A subunit putative*										
PG1623	0.771913	25.59	28	-0.13	5.74	Y	Y	0.012494	-0.65	18151385.5	28031541.9
PGN0473	*membrane bound regulatory protein putative*										
PG1625	0.000031	2.13	23.5	-3.46	4.68	Y	G	0.000226	-5.91	66413	3945435.65
PGN0476	*hypothetical protein PG_1625*										
PG1626	0.000000	25.59	123	-2.27	7.22	G	Y	0.010571	-2.88	11511000.5	81134874.1
PGN0477	*hypothetical protein PG_1626*										

TIGR	PgSgFn / Pg Spectral Count							PgSgFn / Pg Protein Intensity			
PGN	*q*-value	PgSgFn	Pg	log_2Ratio	log_2Sum	Regulation		*q*-value	log_2Ratio	PgSgFn	Pg
PG0234	0.000163	38.38	21.5	0.84	5.9	R	Y	0.054253	0.47	3457803.5	2346171.73
PGN0482	*immunoreactive 23 kDa antigen PG66*										
PG1632	0.000417	18.13	44.5	-1.3	5.97	G	G	0.000584	-1.10	11102545.5	23752020.3
PGN0483	*galM aldose 1-epimerase*										
PG1633	0.290677	91.69	70	0.39	7.34	Y	Y	0.022021	-0.40	59624448	78492841.7
PGN0484	*galK galactokinase*										
PG1635	0.779998	2.13	2.5	-0.23	2.21	Y	Y	0.052485	-1.58	66413	744479.025
PGN0486	*hypothetical protein PG_1635*										
PG1636	0.002655	35.18	60.5	-0.78	6.58	G	Y	0.091569	-0.82	36438452.5	49654214.7
PGN0487	*FtsK/SpoIIIE family protein*										
PG1638	0.308129	7.46	15	-1.01	4.49	Y	Y	0.085610	-2.90	3319775.5	5590282.4
PGN0488	*thioredoxin family protein*										
PG1641	0.758093	2.13	6.5	-1.61	3.11	Y	G	0.003861	-4.93	66413	2048710.73
PGN0491	*phosphotyrosine protein phosphatase*										
PG1615	0.021850	272.94	224.5	0.28	8.96	Y	Y	0.073058	0.07	238750682	227540541
PGN0497	*frdA fumarate reductase flavoprotein subunit*										
PG1614	0.269016	21.32	13	0.71	5.1	Y	G	0.006371	-1.97	2327145	7439696.6
PGN0498	*frdB fumarate reductase iron-sulfur protein*										
PG1613	0.273687	94.89	119.5	-0.33	7.74	Y	Y	0.015803	-0.33	72823294	92005493.9
PGN0499	*glyoxalase family protein*										
PG1612	0.000044	597.07	667	-0.16	10.3	G	Y	0.128525	0.09	729823893	685208281
PGN0500	*mmdA methylmalonyl-CoA decarboxylase alpha subunit*										
PG1611	0.006149	88.49	94	-0.09	7.51	G	R	0.004851	0.63	71767009.5	46955364.3
PGN0501	*hypothetical protein PG_1611*										
PG1609	0.000000	242.02	363.5	-0.59	9.24	G	Y	0.087922	-0.21	80854557.5	91352569.9
PGN0503	*mmdC methylmalonyl-CoA decarboxylase gamma subunit*										
PG1608	0.176852	8.53	7.5	0.19	4	Y	Y	0.052108	-3.50	2640709.5	7280641.88
PGN0504	*mmdB methylmalonyl-CoA decarboxylase beta subunit*										
PG1605	0.363945	165.26	137.5	0.27	8.24	Y	Y	0.156645	-0.02	202259930	204994489
PGN0508	*pepC aminopeptidase C*										
PG1604	0.347604	28.79	40	-0.47	6.1	Y	G	0.007923	-0.68	14754286.5	23455749.4
PGN0509	*immunoreactive 84 kDa antigen PG93*										
PG1603	0.751373	45.85	41.5	0.14	6.45	Y	Y	0.140843	-0.01	25840992.5	26047181.9
PGN0510	*HAM1 protein*										
PG1602	0.482754	19.19	11.5	0.74	4.94	Y	Y	0.027355	-1.55	1426523	4681957.95
PGN0511	*conserved hypothetical protein*										
PG1597	0.188231	11.73	16	-0.45	4.79	Y	Y	0.100194	-3.59	9843864.5	14886068.8
PGN0516	*DnaK suppressor protein putative*										
PG1596	0.001627	232.43	305	-0.39	9.07	G	G	0.006115	-0.33	105529295	132698183
PGN0517	*isoleucyl-tRNA synthetase putative*										
PG1595	0.000000	2.13	54.5	-4.68	5.82	Y	G	0.000007	-7.23	66413	9863722.58
PGN0518	*rpe ribulose-phosphate 3-epimerase*										
PG1592	0.069053	40.52	62.5	-0.63	6.69	Y	G	0.009001	-0.63	10596692.5	16117967.9
PGN0521	*HDIG domain protein*										
PG1589	0.000575	9.6	29	-1.59	5.27	G	Y	0.037877	-3.77	3150847	10839301.4
PGN0522	*folP dihydropteroate synthase*										
PG1588	0.751373	2.13	3	-0.49	2.36	Y	Y	0.052108	-2.13	66413	1581429.98
PGN0523	*conserved hypothetical protein*										

TIGR	PgSgFn / Pg Spectral Count						PgSgFn / Pg Protein Intensity				
PGN	*q*-value	PgSgFn	Pg	log_2Ratio	log_2Sum	Regulation	Regulation	*q*-value	log_2Ratio	PgSgFn	Pg
PG1587	0.725943	2.13	7	-1.72	3.19	Y	G	0.003160	-4.63	66413	1657821.78
PGN0524	*PAP2 superfamily protein*										
PG1586	0.516095	9.6	9	0.09	4.22	Y	Y	0.161524	-2.80	5476541	6367358.58
PGN0525	*batE batE protein*										
PG1585	0.786857	29.85	29	0.04	5.88	Y	Y	0.087085	-0.11	14633014	15713343.7
PGN0526	*batD batD protein*										
PG1584	0.115710	21.32	11.5	0.89	5.04	Y	Y	0.053020	0.82	7081698.5	3284526.6
PGN0527	*batC batC protein*										
PG1583	0.634798	2.13	8	-1.91	3.34	Y	G	0.004244	-5.71	66413	3540492.28
PGN0528	*batB batB protein*										
PG1582	0.240620	2.13	11	-2.37	3.71	Y	G	0.001091	-5.44	66413	2876662.53
PGN0529	*batA batA protein*										
PG1581	0.056380	15.99	19	-0.25	5.13	Y	Y	0.053020	-3.63	6272231	12060820.1
PGN0530	*hypothetical protein PG_1581*										
PG1580	0.000233	19.19	29	-0.6	5.59	G	Y	0.128525	-3.10	4708185	6258223.2
PGN0531	*conserved hypothetical protein*										
PG1579	0.687551	74.63	68.5	0.12	7.16	Y	Y	0.066072	-0.31	46777684.5	59949389.7
PGN0532	*ATPase MoxR family*										
PG1578	0.000000	10.66	45.5	-2.09	5.81	G	G	0.005281	-4.35	2256621.5	11958740.7
PGN0533	*nadA quinolinate synthetase complex subunit A*										
PG1577	0.024693	2.13	15	-2.82	4.1	Y	Y	0.020209	-8.50	66413	28797934.7
PGN0534	*nadC nicotinate-nucleotide pyrophosphorylase*										
PG1576	0.125058	2.13	12.5	-2.55	3.87	Y	G	0.004413	-6.47	66413	5985868.28
PGN0535	*nadB L-aspartate oxidase*										
PG1573	0.126258	6.4	4.5	0.51	3.45	Y	Y	0.089345	1.22	1808844.5	719518.85
PGN0537	*transcriptional regulator Crp family*										
PG1571	0.751373	2.13	3	-0.49	2.36	Y	Y	0.053020	-1.30	66413	662766
PGN0539	*metallo-beta-lactamase superfamily protein*										
PG1566	0.751373	92.76	100.5	-0.12	7.59	Y	Y	0.025545	-0.09	52939061.5	56159760.3
PGN0543	*gltX glutamyl-tRNA synthetase*										
PG1565	0.004789	2.13	17	-3	4.26	Y	Y	0.010571	-5.68	66413	3658479
PGN0544	*3-deoxy-D-manno-octulosonic-acid transferase putative*										
PG1564	0.658227	2.13	7.5	-1.82	3.27	Y	Y	0.030979	-3.95	66413	1397470
PGN0545	*membrane protein putative*										
PG1563	0.629057	67.17	58	0.21	6.97	Y	Y	0.013957	0.25	33800921.5	28588421.9
PGN0546	*rfbA glucose-1-phosphate thymidylyltransferase*										
PG1562	0.751373	20.26	24	-0.24	5.47	Y	G	0.009495	-0.49	7114888	9942694
PGN0547	*rfbC dTDP-4-dehydrorhamnose 35-epimerase*										
PG1561	0.000436	8.53	28.5	-1.74	5.21	G	Y	0.029520	-3.58	2753764	6703881.65
PGN0548	*rfbD dTDP-4-dehydrorhamnose reductase*										
PG1560	0.420012	37.32	39	-0.06	6.25	Y	Y	0.068558	0.43	23472072.5	16353768.7
PGN0549	*rfbB dTDP-glucose 46-dehydratase*										
PG1559	0.074667	303.86	312.5	-0.04	9.27	Y	Y	0.052485	0.09	267532608	252362979
PGN0550	*gcvT glycine cleavage system T protein*										
PG1555	0.600536	37.32	48	-0.36	6.41	Y	Y	0.119527	0.08	16867974	15834185.9
PGN0554	*conserved domain protein*										
PG1553	0.102044	1357.26	1265	0.1	11.36	Y	Y	0.027084	-0.03	980922612	1.001e+09
PGN0556	*CobN/magnesium chelatase family protein*										

TIGR	PgSgFn / Pg Spectral Count							PgSgFn / Pg Protein Intensity			
PGN	*q*-value	PgSgFn	Pg	log_2Ratio	log_2Sum	Regulation		*q*-value	log_2Ratio	PgSgFn	Pg
PG1552	0.001746	1265.57	1207	0.07	11.27	R	Y	0.013354	0.20	424713377	368717694
PGN0557	*hmuR TonB-dependent receptor HmuR*										
PG1551	0.000000	967.03	732	0.4	10.73	R	R	0.003103	0.46	1.245e+09	903273515
PGN0558	*hmuY hmuY protein*										
PG1545	0.017292	445.67	388	0.2	9.7	Y	Y	0.062174	-0.24	347181838	417426758
PGN0564	*sodB superoxide dismutase Fe-Mn*										
PG1543	0.700541	20.26	24.5	-0.27	5.48	Y	G	0.003026	-1.30	8223660	19587604
PGN0566	*thioesterase family protein*										
PG1542	0.403113	2.13	10	-2.23	3.6	Y	Y	0.011148	-6.08	66413	4836386
PGN0567	*prtC collagenase*										
PG1541	0.751373	2.13	3	-0.49	2.36	Y	Y	0.052147	-1.74	66413	926555.925
PGN0568	*folK 2-amino-4-hydroxy-6-hydroxymethyldihydropteridine pyrophosphokinase*										
PG1540	0.000397	66.1	110	-0.73	7.46	G	G	0.002914	-0.47	48861317.5	67693048.6
PGN0569	*queA S-adenosylmethionine:tRNA ribosyltransferase-isomerase*										
PG1536	0.345160	2.13	10	-2.23	3.6	Y	Y	0.014040	-6.01	66413	4732513.58
PGN0573	*cell division protein FtsX putative*										
PG1490	0.001057	8.53	1	3.09	3.25	Y	Y	0.051674	4.85	3875045	23108.5
PGN0579	*TraG family protein*										
PG1495	0.692989	2.13	3.5	-0.72	2.49	Y	Y	0.052108	-1.99	66413	1318925.08
PGN0582	*topB-2 DNA topoisomerase III*										
PG1479	0.751373	2.13	3	-0.49	2.36	Y	Y	0.056249	-1.02	66413	352780.05
PGN0598	*conjugative transposon protein TraJ*										
PG1286	0.000000	776.19	1130	-0.54	10.9	G	G	0.005850	-0.66	786745187	1.2428e+09
PGN0604	*ftn ferritin*										
PG1285	0.000000	2.13	36.5	-4.1	5.27	Y	G	0.000107	-7.50	66413	11871032.9
PGN0606	*glucosamine-6-phosphate isomerase putative*										
PG1283	0.002971	347.58	420.5	-0.27	9.59	G	Y	0.014040	-0.13	239731394	261770555
PGN0607	*conserved hypothetical protein*										
PG1281	0.000443	2.13	19.5	-3.19	4.43	Y	G	0.007727	-7.25	66413	10598825.2
PGN0609	*hypothetical protein PG_1281*										
PG1280	0.000381	358.24	311.5	0.2	9.39	R	Y	0.053939	0.08	335988443	318399293
PGN0610	*conserved hypothetical protein*										
PG1279	0.000000	670.63	985	-0.55	10.69	G	Y	0.052108	0.28	432354674	351186442
PGN0611	*D-isomer specific 2-hydroxyacid dehydrogenase family protein*										
PG1278	0.004530	2014.03	2113	-0.07	12.01	G	Y	0.022021	-0.08	1.8822e+09	1.9891e+09
PGN0612	*serC phosphoserine aminotransferase*										
PG1277	0.751373	28.79	34	-0.24	5.97	Y	Y	0.013080	-0.60	16515024.5	25291205.8
PGN0613	*UDP-glucose-6 dehydrogenase putative*										
PG0571	0.659176	90.63	96	-0.08	7.54	Y	Y	0.032546	-0.84	30578894.5	58679955.7
PGN0618	*asd aspartate-semialdehyde dehydrogenase*										
PG0573	0.475857	2.13	9	-2.08	3.48	Y	G	0.005000	-5.43	66413	2937574.18
PGN0620	*mraW S-adenosyl-methyltransferase MraW*										
PG0575	0.358444	26.65	17	0.65	5.45	Y	Y	0.012690	1.25	7338382	3019542
PGN0622	*penicillin-binding protein 2 putative*										
PG0576	0.410849	27.72	39	-0.49	6.06	Y	G	0.001876	-1.31	8683356.5	21487545.4
PGN0623	*murE UDP-N-acetylmuramoylalanyl-D-glutamyl-2 6-diaminopimelate ligase*										
PG0578	0.000000	2.13	38.5	-4.18	5.34	Y	G	0.003294	-7.60	66413	13041684.5
PGN0625	*murD UDP-N-acetylmuramoylalanine--D-glutamate ligase*										

TIGR	PgSgFn / Pg Spectral Count						PgSgFn / Pg Protein Intensity				
PGN	*q*-value	PgSgFn	Pg	log_2Ratio	log_2Sum	Regulation		*q*-value	log_2Ratio	PgSgFn	Pg
PG0580	0.035356	13.86	22	-0.67	5.16	Y	Y	0.094935	-3.02	4265870.5	6526242.05
PGN0627	*murG UDP-N-acetylglucosamine--N-acetylmuramyl-(pentapeptide) pyrophosphoryl-undecaprenol N-acetylglucosamine transferase*										
PG0581	0.003644	2.13	17.5	-3.04	4.29	Y	Y	0.017881	-6.55	66413	7217321.95
PGN0628	*murC UDP-N-acetylmuramate--alanine ligase*										
PG0583	0.292751	102.35	85.5	0.26	7.55	Y	Y	0.042501	-0.11	37054351.5	40087110.8
PGN0630	*ftsA cell division protein FtsA*										
PG0584	0.025621	38.38	63	-0.71	6.66	Y	Y	0.015691	-0.87	17416508	32654111.4
PGN0631	*ftsz cell division protein FtsZ*										
PG0585	0.013991	73.57	107	-0.54	7.5	Y	Y	0.040104	-0.26	42628927	50449284.2
PGN0632	*YqeY family protein*										
PG0588	0.771913	26.65	21.5	0.31	5.59	Y	R	0.006488	0.92	15516357	8254904.85
PGN0634	*panB 3-methyl-2-oxobutanoate hydroxymethyltransferase*										
PG0589	0.243063	237.76	237	0	8.89	Y	G	0.004627	-0.48	68840319	95976917.1
PGN0635	*guaA GMP synthase*										
PG0592	0.029864	54.38	31	0.81	6.42	Y	R	0.003852	0.73	96046289.5	57907274.2
PGN0636	*rpmE ribosomal protein L31*										
PG0593	0.397614	66.1	78.5	-0.25	7.18	Y	Y	0.054253	-0.26	52547106.5	64193890.8
PGN0637	*htrA htrA protein*										
PG0594	0.779998	30.92	33.5	-0.12	6.01	Y	Y	0.192818	-0.09	13712523.5	13671002.5
PGN0638	*rpoD RNA polymerase sigma-70 factor*										
PG0595	0.548692	127.94	139	-0.12	8.06	Y	Y	0.017665	0.27	86262499.5	71388445.7
PGN0639	*rpsF ribosomal protein S6*										
PG0596	0.012939	30.92	55	-0.83	6.42	Y	G	0.007304	-0.70	39007766.5	62980873.3
PGN0640	*rpsR ribosomal protein S18*										
PG0597	0.007997	105.55	73.5	0.52	7.48	R	Y	0.017839	0.22	91919371.5	78995957.6
PGN0641	*rplI ribosomal protein L9*										
PG0599	0.089835	218.57	220	-0.01	8.78	Y	Y	0.030979	-0.29	124759660	153182621
PGN0643	*ribBA 34-dihydroxy-2-butanone 4-phosphate synthase/GTP cyclohydrolase II*										
PG0602	0.357051	30.92	44	-0.51	6.23	Y	G	0.002260	-0.98	14555603	28601247
PGN0645	*hypothetical protein PG_0602*										
PG0603	0.035356	2.13	14.5	-2.77	4.06	Y	G	0.005259	-6.82	66413	7723477.7
PGN0646	*cmk cytidylate kinase*										
PG0611	0.021557	35.18	17	1.05	5.71	Y	Y	0.090791	-0.15	30136616	33566719.8
PGN0654	*lipoprotein putative*										
PG0613	0.005103	60.77	97	-0.67	7.3	G	Y	0.037877	-0.47	57744705.5	81546522.1
PGN0656	*hypothetical protein PG_0613*										
PG0614	0.355429	58.64	76.5	-0.38	7.08	Y	Y	0.013691	-0.34	70507661.5	88613623.5
PGN0657	*hypothetical protein PG_0614*										
PG0615	0.000000	44.78	105.5	-1.24	7.23	G	G	0.005011	-1.21	11415726.5	26367064.2
PGN0658	*typA GTP-binding protein TypA*										
PG0616	0.000000	804.97	626	0.36	10.48	R	Y	0.037727	0.17	944284780	833567206
PGN0659	*thioredoxin putative*										
PG0618	0.125451	292.14	242.5	0.27	9.06	Y	Y	0.191557	0.00	476288475	476061382
PGN0660	*alkyl hydroperoxide reductase C subunit*										
PG0619	0.625271	18.13	25.5	-0.49	5.45	Y	Y	0.168269	0.01	11717294	11612563.9
PGN0661	*alkyl hydroperoxide reductase F subunit*										
PG0620	0.164350	275.08	268	0.04	9.09	Y	G	0.009306	-0.38	77651462.5	100640402
PGN0662	*lon ATP-dependent protease La*										

TIGR	PgSgFn / Pg Spectral Count							PgSgFn / Pg Protein Intensity			
**PGN**	*q*-value	PgSgFn	Pg	log_2Ratio	log_2Sum	Regulation		*q*-value	log_2Ratio	PgSgFn	Pg
PG0621	0.751373	27.72	34	-0.29	5.95	Y	Y	0.055401	1.10	98928695.5	19008879.3
PGN0663	*conserved hypothetical protein*										
PG0622	0.275068	2.13	11	-2.37	3.71	Y	G	0.003984	-5.93	66413	4110056.78
PGN0664	*hypothetical protein PG_0622*										
PG0623	0.000000	1013.95	698	0.54	10.74	R	R	0.008458	0.35	676686846	534120385
PGN0665	*tpiA triosephosphate isomerase*										
PG0624	0.784897	2.13	5.5	-1.37	2.93	Y	G	0.001427	-5.30	66413	2615973.1
PGN0666	*hypothetical protein PG_0624*										
PG0625	0.600663	33.05	24.5	0.43	5.85	Y	Y	0.046400	0.74	10734122	6619482.35
PGN0667	*folE GTP cyclohydrolase I*										
PG0627	0.779998	39.45	44.5	-0.17	6.39	Y	Y	0.014837	-0.29	27125320	32978640.1
PGN0668	*RNA-binding protein*										
PG0629	0.197758	7.46	15	-1.01	4.49	Y	Y	0.093332	-3.39	5226177	8077927.83
PGN0670	*ppnK ATP-NAD kinase*										
PG0630	0.778744	35.18	35.5	-0.01	6.14	Y	Y	0.014335	-0.68	22624813	35158733.4
PGN0671	*pdxJ pyridoxal phosphate biosynthetic protein PdxJ*										
PG0631	0.690619	49.04	42	0.22	6.51	Y	Y	0.012144	-0.36	24700447.5	31461498
PGN0672	*MotA/TolQ/ExbB proton channel family protein*										
PG0632	0.630045	22.39	25	-0.16	5.57	Y	G	0.004902	-0.73	4513237.5	7397268.13
PGN0673	*biopolymer transport protein ExbD putative*										
PG0633	0.392640	9.6	10.5	-0.13	4.33	Y	Y	0.058540	-2.46	1382424.5	2492461.2
PGN0674	*hypothetical protein PG_0633*										
PG0634	0.012279	116.21	92.5	0.33	7.71	Y	R	0.000238	0.61	54439025	35619535.4
PGN0675	*ThiJ/PfpI family protein*										
PG0637	0.751373	2.13	3	-0.49	2.36	Y	Y	0.052108	-2.23	66413	2378005.5
PGN0678	*thiL thiamine monophosphate kinase*										
PG0638	0.017728	17.06	23	-0.43	5.32	Y	Y	0.014536	-3.84	2827358	9463266.63
PGN0679	*lpxK tetraacyldisaccharide 4'-kinase*										
PG0639	0.448384	170.59	144	0.24	8.3	Y	Y	0.060576	0.10	114494608	106977821
PGN0680	*sppA signal peptide peptidase SppA 67K type*										
PG0645	0.751373	26.65	24	0.15	5.66	Y	Y	0.149560	-0.13	6939358	7365310.53
PGN0684	*conserved domain protein*										
PG0646	0.188231	8.53	6.5	0.39	3.91	Y	Y	0.078622	-1.76	11059040.5	3822612.28
PGN0685	*iron compound ABC transporter ATP-binding protein*										
PG0648	0.634798	2.13	8	-1.91	3.34	Y	G	0.000381	-5.49	66413	2955672.45
PGN0687	*iron compound ABC transporter periplasmic iron compound-binding protein putative*										
PG0651	0.779998	2.13	6	-1.49	3.02	Y	G	0.006115	-4.83	66413	1945207.8
PGN0690	*HDIG domain protein*										
PG0652	0.001893	70.37	50	0.49	6.91	R	Y	0.037176	-0.35	9162947	11533551.4
PGN0691	*conserved hypothetical protein*										
PG0653	0.022994	29.85	53	-0.83	6.37	Y	Y	0.161524	-0.34	18696524.5	20263029.4
PGN0692	*serB phosphoserine phosphatase*										
PG0654	0.553580	40.52	38	0.09	6.29	Y	Y	0.097582	-0.13	59627423	64457787.6
PGN0693	*hypothetical protein PG_0654*										
PG0657	0.560362	2.13	8.5	-2	3.41	Y	G	0.009634	-3.35	66413	713082.85
PGN0695	*maf maf protein*										
PG0658	0.414614	21.32	14.5	0.56	5.16	Y	G	0.003925	-0.80	6544966	11419881.2
PGN0696	*phosphatase YrbI family*										

TIGR	PgSgFn / Pg Spectral Count							PgSgFn / Pg Protein Intensity			
PGN	*q*-value	PgSgFn	Pg	log_2Ratio	log_2Sum	Regulation		*q*-value	log_2Ratio	PgSgFn	Pg
PG0660	0.124597	9.6	18	-0.91	4.79	Y	Y	0.016357	-3.73	2479433	8256007.23
PGN0698	*nitroreductase family protein*										
PG0664	0.034230	23.46	40.5	-0.79	6	Y	Y	0.026006	-0.97	13382059	26868149.3
PGN0700	*oxidoreductase Gfo/Idh/MocA family*										
PG0665	0.015259	2.13	15.5	-2.86	4.14	Y	Y	0.010571	-4.77	66413	1944008.03
PGN0701	*lacZ-1 beta-galactosidase*										
PG0668	0.000000	215.37	404.5	-0.91	9.28	G	G	0.006916	-0.57	115872717	171957908
PGN0704	*TonB-dependent receptor*										
PG0669	0.115710	353.97	408	-0.2	9.57	Y	Y	0.019571	-0.21	384804408	444895852
PGN0705	*fetB heme-binding protein FetB*										
PG0670	0.102520	17.06	31.5	-0.88	5.6	Y	Y	0.086344	-0.46	11865247.5	14835903
PGN0706	*lipoprotein putative*										
PG0672	0.751373	2.13	3	-0.49	2.36	Y	Y	0.052108	-1.70	66413	1143500
PGN0708	*iron compound ABC transporter ATP-binding protein*										
PG0674	0.310078	84.23	64	0.4	7.21	Y	R	0.006700	0.44	90688124	67060438.8
PGN0709	*iorB indolepyruvate ferredoxin oxidoreductase beta subunit*										
PG0675	0.394844	395.56	387.5	0.03	9.61	Y	R	0.000584	0.47	257271663	186116641
PGN0710	*iorA indolepyruvate ferredoxin oxidoreductase alpha subunit*										
PG0676	0.130515	124.74	95	0.39	7.78	Y	Y	0.055401	0.05	109546459	105644334
PGN0711	*oxidoreductase short chain dehydrogenase/reductase family*										
PG0677	0.629057	90.63	99	-0.13	7.57	Y	Y	0.177260	0.01	41342935	42046316.3
PGN0713	*LYS1 saccharopine dehydrogenase*										
PG0678	0.773021	2.13	6.5	-1.61	3.11	Y	G	0.006475	-5.87	66413	4036026.5
PGN0714	*pyrazinamidase/nicotinamidase putative*										
PG0686	0.522042	302.8	269.5	0.17	9.16	Y	Y	0.067266	-0.07	165194851	173749743
PGN0722	*conserved hypothetical protein*										
PG0687	0.000000	4314.87	5735	-0.41	13.29	G	Y	0.020083	0.16	4.3387e+09	3.8906e+09
PGN0723	*sucD succinate-semialdehyde dehydrogenase*										
PG0689	0.000000	1643	1998	-0.28	11.83	G	Y	0.039451	-0.12	1.8209e+09	1.9759e+09
PGN0724	*4hbD NAD-dependent 4-hydroxybutyrate dehydrogenase*										
PG0690	0.000000	711.15	1109	-0.64	10.83	G	G	0.007211	-0.34	507114556	643634260
PGN0725	*abfT-1 4-hydroxybutyrate CoA-transferase*										
PG0691	0.758093	2.13	6.5	-1.61	3.11	Y	G	0.001728	-6.63	66413	6591124.8
PGN0726	*NifU-related protein*										
PG0692	0.216220	5587.89	5420.5	0.04	13.43	Y	Y	0.147498	-0.03	4.1133e+09	4.2002e+09
PGN0727	*abfD 4-hydroxybutyryl-CoA dehydratase*										
PG0694	0.000000	1803.99	2501.5	-0.47	12.07	G	Y	0.119527	0.04	1.8822e+09	1.8272e+09
PGN0728	*immunoreactive 42 kDa antigen PG33*										
PG0695	0.000000	1302.88	1410	-0.11	11.41	G	Y	0.032387	-0.14	1.6837e+09	1.8518e+09
PGN0729	*immunoreactive 43 kDa antigen PG32*										
PG0698	0.779998	2.13	2.5	-0.23	2.21	Y	Y	0.052108	-2.25	66413	1884872.75
PGN0732	*lipoprotein putative*										
PG0699	0.008708	92.76	135	-0.54	7.83	G	G	0.005259	-0.78	35823963.5	61565342.7
PGN0733	*malP maltodextrin phosphorylase*										
PG0701	0.658227	2.13	7.5	-1.82	3.27	Y	G	0.009241	-5.47	66413	3131346.93
PGN0735	*cobU cobinamide kinase/cobinamide phosphate guanylyltransferase*										
PG0702	0.634798	2.13	8	-1.91	3.34	Y	G	0.001865	-5.76	66413	3599967.9
PGN0736	*nicotinate-nucleotide--dimethylbenzimidazole phosphoribosyltransferase putative*										

TIGR / *PGN*	PgSgFn / Pg Spectral Count *q*-value	PgSgFn	Pg	log_2Ratio	log_2Sum	Regulation		PgSgFn / Pg Protein Intensity *q*-value	log_2Ratio	PgSgFn	Pg
PG0704	0.784897	2.13	2	0.09	2.05	Y	Y	0.052108	-1.90	66413	1152105.78
PGN0738	*phosphoglycerate mutase family protein*										
PG0705	0.278503	67.17	85	-0.34	7.25	Y	Y	0.014081	-0.61	24341980	36882212.3
PGN0739	*murI glutamate racemase*										
PG0706	0.600049	2.13	8.5	-2	3.41	Y	G	0.008512	-5.75	66413	3764947.08
PGN0740	*hypothetical protein PG_0706*										
PG0707	0.004964	721.81	787	-0.12	10.56	G	Y	0.104719	-0.07	483641686	508809189
PGN0741	*TonB-dependent receptor putative*										
PG0708	0.000000	334.78	557	-0.73	9.8	G	G	0.004902	-0.12	243576699	264610247
PGN0742	*peptidyl-prolyl cis-trans isomerase FKBP-type*										
PG0709	0.422376	83.16	66.5	0.32	7.23	Y	Y	0.052108	0.15	59017389.5	53161089.8
PGN0743	*fkpA peptidyl-prolyl cis-trans isomerase FkpA FKBP-type*										
PG0710	0.043835	67.17	76.5	-0.19	7.17	Y	Y	0.078622	0.07	27055007	25777251.7
PGN0744	*peptidyl-prolyl cis-trans isomerase FKBP-type*										
PG0712	0.779998	24.52	28	-0.19	5.71	Y	Y	0.030145	-0.49	11798919.5	16955132.6
PGN0746	*hypothetical protein PG_0712*										
PG0713	0.475857	2.13	9	-2.08	3.48	Y	G	0.000743	-5.17	66413	2367320
PGN0747	*trpG anthranilate synthase component II*										
PG0714	0.000003	2.13	26	-3.61	4.81	Y	G	0.000052	-7.81	66413	14746401.6
PGN0750	*cutC copper homeostasis protein CutC*										
PG0715	0.014718	2.13	16	-2.91	4.18	Y	G	0.002733	-6.35	66413	5441875.48
PGN0751	*transporter*										
PG0724	0.779998	213.24	219.5	-0.04	8.76	Y	G	0.007580	-0.58	141043767	210366536
PGN0756	*prolyl oligopeptidase family protein*										
PG0725	0.264342	2.13	11	-2.37	3.71	Y	G	0.004508	-5.57	66413	3204241.43
PGN0757	*hydrolase haloacid dehalogenase-like family*										
PG0726	0.000719	83.16	52.5	0.66	7.08	R	Y	0.026630	-0.38	89450626	115653052
PGN0758	*lipoprotein putative*										
PG0728	0.549619	2.13	9	-2.08	3.48	Y	G	0.001091	-5.93	66413	4021974.58
PGN0759	*conserved hypothetical protein*										
PG0729	0.053706	2.13	14	-2.72	4.01	Y	G	0.000107	-7.34	66413	10633168.4
PGN0760	*ddlA D-alanine--D-alanine ligase*										
PG0731	0.692989	2.13	3.5	-0.72	2.49	Y	Y	0.052108	-2.21	66413	1776709.13
PGN0762	*hypothetical protein PG_0731*										
PG0733	0.786859	92.76	94	-0.02	7.55	Y	Y	0.034691	-0.39	53454561.5	69757810.5
PGN0764	*ribE riboflavin synthase alpha subunit*										
PG0734	0.229573	8.53	12.5	-0.55	4.39	Y	Y	0.163917	-2.79	7819505	6650003.05
PGN0765	*nitroreductase family protein*										
PG0735	0.000000	9.6	52.5	-2.45	5.96	G	G	0.008795	-4.39	2686443	13656867.8
PGN0766	*aminotransferase class V*										
PG0738	0.779998	2.13	2.5	-0.23	2.21	Y	Y	0.057050	-0.97	66413	327749.525
PGN0769	*cytidine/deoxycytidylate deaminase family protein*										
PG0739	0.608229	2.13	8.5	-2	3.41	Y	G	0.003547	-5.85	66413	3871715.88
PGN0770	*metallo-beta-lactamase family protein*										
PG0745	0.779998	2.13	2.5	-0.23	2.21	Y	Y	0.054755	-1.18	66413	437767.925
PGN0773	*lactoylglutathione lyase putative*										
PG0747	0.507925	2.13	4.5	-1.08	2.73	Y	Y	0.052108	-2.06	66413	1450407.38
PGN0775	*sigma-54 dependent DNA-binding response regulator*										

TIGR	PgSgFn / Pg Spectral Count							PgSgFn / Pg Protein Intensity			
PGN	*q*-value	PgSgFn	Pg	log_2Ratio	log_2Sum	Regulation		*q*-value	log_2Ratio	PgSgFn	Pg
PG0750	0.720026	2.13	7.5	-1.82	3.27	Y	G	0.001156	-5.08	66413	2230381.6
PGN0777	*glycosyl transferase group 2 family protein*										
PG0751	0.340826	10.66	14	-0.39	4.62	Y	Y	0.087085	-3.06	4166263	6588936.28
PGN0778	*porT porT protein*										
PG0752	0.000003	2.13	26	-3.61	4.81	Y	G	0.000091	-8.45	66413	22990565.9
PGN0779	*uracil phosphoribosyltransferase putative*										
PG0754	0.001057	28.79	59	-1.04	6.46	G	Y	0.090791	-0.53	25035539	31973379.1
PGN0781	*topA DNA topoisomerase I*										
PG0756	0.217007	2.13	11	-2.37	3.71	Y	G	0.000436	-5.90	66413	3936955.98
PGN0786	*conserved hypothetical protein*										
PG0757	0.009465	10.66	20	-0.91	4.94	G	Y	0.013019	-4.04	1132841	7121154.15
PGN0787	*hypothetical protein PG_0757*										
PG0758	0.000003	639.71	508	0.33	10.16	R	Y	0.014816	0.09	586425120	551452784
PGN0788	*dcp-1 peptidyl-dipeptidase Dcp*										
PG0759	0.000016	55.44	104.5	-0.91	7.32	G	Y	0.014959	-0.43	37204969.5	49960382.3
PGN0789	*TPR domain protein*										
PG0762	0.550186	198.31	218	-0.14	8.7	Y	Y	0.178380	-0.03	225048943	227601445
PGN0791	*trigger factor putative*										
PG0766	0.630045	546.95	542.5	0.01	10.09	Y	Y	0.190624	0.01	621108363	620143793
PGN0792	*pnpA polyribonucleotide nucleotidyltransferase*										
PG0767	0.553580	29.85	35	-0.23	6.02	Y	Y	0.030979	0.51	15429387	10989666.1
PGN0793	*malQ 4-alpha-glucanotransferase*										
PG0768	0.134175	2.13	12.5	-2.55	3.87	Y	G	0.000581	-5.87	66413	3845135.75
PGN0794	*conserved hypothetical protein*										
PG0769	0.408616	88.49	81	0.13	7.41	Y	Y	0.136314	-0.09	63188268	66623403.5
PGN0795	*fibronectin type III domain protein*										
PG0775	0.000000	765.52	982	-0.36	10.77	G	Y	0.019334	0.21	561140531	484665261
PGN0799	*acyl-CoA dehydrogenase family protein*										
PG0776	0.000000	840.16	689.5	0.29	10.58	R	Y	0.149560	0.03	557072609	547060238
PGN0800	*etfA-1 electron transfer flavoprotein alpha subunit*										
PG0777	0.013991	1066.19	940	0.18	10.97	Y	Y	0.044266	0.23	589001999	498493839
PGN0801	*etfB-1 electron transfer flavoprotein beta subunit*										
PG0778	0.751373	2.13	3	-0.49	2.36	Y	Y	0.052108	-2.04	66413	1400693
PGN0802	*conserved hypothetical protein*										
PG0779	0.751373	56.51	64	-0.18	6.91	Y	Y	0.076556	-0.04	77819241	80061237.4
PGN0803	*hypothetical protein PG_0779*										
PG0780	0.549619	123.68	138	-0.16	8.03	Y	Y	0.045407	-0.35	157527463	199569315
PGN0804	*hypothetical protein PG_0780*										
PG0782	0.000000	683.43	497.5	0.46	10.21	R	Y	0.109736	-0.02	1.5235e+09	1.5469e+09
PGN0806	*MotA/TolQ/ExbB proton channel family protein*										
PG0784	0.000130	89.56	49	0.87	7.11	R	Y	0.077211	-0.15	27602312.5	30490724.4
PGN0808	*polyprenyl synthetase*										
PG0785	0.602724	93.82	102	-0.12	7.61	Y	Y	0.058540	-0.27	153126680	187230371
PGN0809	*tonB protein putative*										
PG0788	0.134175	2.13	12.5	-2.55	3.87	Y	G	0.000159	-6.22	66413	4910824.55
PGN0811	*hypothetical protein PG_0788*										
PG0789	0.779998	2.13	2.5	-0.23	2.21	Y	Y	0.060075	-0.80	66413	263545.75
PGN0812	*conserved hypothetical protein*										

TIGR	PgSgFn / Pg Spectral Count							PgSgFn / Pg Protein Intensity			
PGN	*q*-value	PgSgFn	Pg	log_2Ratio	log_2Sum	Regulation		*q*-value	log_2Ratio	PgSgFn	Pg
PG0790	0.020067	25.59	10.5	1.29	5.17	Y	R	0.002035	1.97	14004852.5	3628265
PGN0813	*obg GTP-binding protein Obg*										
PG0791	0.064532	125.81	94	0.42	7.78	Y	Y	0.149560	0.02	43209519	42434562.8
PGN0814	*adk adenylate kinase*										
PG0792	0.251314	6.4	14	-1.13	4.35	Y	Y	0.104719	-3.13	4042571.5	5965923.08
PGN0815	*hpt hypoxanthine phosphoribosyltransferase*										
PG0793	0.516095	197.24	170	0.21	8.52	Y	Y	0.094935	-0.15	163502494	179900032
PGN0816	*fbp fructose-16-bisphosphatase*										
PG0794	0.608229	23.46	30.5	-0.38	5.75	Y	Y	0.080059	-0.19	7522304	8745994.8
PGN0817	*penicillin-binding protein 1A putative*										
PG0796	0.009526	60.77	93.5	-0.62	7.27	G	Y	0.052108	-0.45	33484534.5	43777106.1
PGN0819	*leuS leucyl-tRNA synthetase*										
PG0800	0.560362	2.13	8.5	-2	3.41	Y	Y	0.014025	-4.00	66413	1167674.98
PGN0823	*conserved hypothetical protein*										
PG0801	0.600049	2.13	8.5	-2	3.41	Y	G	0.000044	-5.28	66413	2556548.88
PGN0824	*polyA polymerase family protein*										
PG0802	0.429193	88.49	71.5	0.31	7.32	Y	Y	0.017767	-0.33	70519436	88902501.8
PGN0826	*pdhD alpha keto acid dehydrogenase complex E3 component lipoamide dehydrogenase*										
PG0803	0.074026	10.66	16.5	-0.63	4.76	Y	Y	0.161132	-3.17	13142267.5	10979458.2
PGN0827	*nagB glucosamine-6-phosphate isomerase*										
PG0804	0.123033	150.33	177	-0.24	8.35	Y	Y	0.130581	0.07	180453867	169058428
PGN0828	*flavodoxin*										
PG0805	0.418300	6.4	11.5	-0.85	4.16	Y	Y	0.088585	-3.23	4022770.5	6405081.55
PGN0829	*lgt prolipoprotein diacylglyceryl transferase*										
PG0806	0.000028	6.4	30.5	-2.25	5.21	G	G	0.001168	-5.44	2396082	22498789.8
PGN0830	*oxidoreductase Gfo/Idh/MocA family*										
PG0807	0.024177	71.43	44	0.7	6.85	Y	Y	0.018197	0.54	42721521.5	29015884.6
PGN0831	*NusB family protein*										
PG0809	0.194026	82.1	95.5	-0.22	7.47	Y	G	0.007601	-0.71	23617242.5	38964422.9
PGN0832	*hypothetical protein PG_0809*										
PG0811	0.765921	22.39	17.5	0.36	5.32	Y	G	0.000238	-0.45	9771882.5	13325933
PGN0833	*ruvA Holliday junction DNA helicase RuvA*										
PG1493	0.000393	14.93	39.5	-1.4	5.77	G	G	0.001694	-1.52	5124476.5	14228449.1
PGN0836	*hypothetical protein PG_1493*										
PG1492	0.000148	12.79	2.5	2.36	3.93	Y	Y	0.055401	2.15	16726092	1858834.48
PGN0837	*hypothetical protein PG_1492*										
PG1433	0.097639	28.79	17.5	0.72	5.53	Y	Y	0.019334	0.61	18451228	11842858.4
PGN0840	*hydrolase*										
PG1434	0.173614	2.13	12	-2.49	3.82	Y	G	0.008458	-4.97	66413	2182369.9
PGN0841	*ispD 4-diphosphocytidyl-2C-methyl-D-erythritol synthase*										
PG1374	0.003339	302.8	239.5	0.34	9.08	R	Y	0.059515	0.19	205559437	179667093
PGN0852	*immunoreactive 47 kDa antigen PG97*										
PG1379	0.225676	36.25	41.5	-0.2	6.28	Y	Y	0.049354	-0.39	15485633	19688930.4
PGN0857	*ABC transporter periplasmic substrate-binding protein putative*										
PG1382	0.000108	68.24	115.5	-0.76	7.52	G	Y	0.012832	-0.34	68161283.5	86861735.2
PGN0860	*hypothetical protein PG_1382*										
PG1397	0.587072	94.89	110	-0.21	7.68	Y	Y	0.157620	-0.06	74575189.5	76782195.6
PGN0865	*purH phosphoribosylaminoimidazolecarboxamide formyltransferase/IMP cyclohydrolase*										

TIGR	PgSgFn / Pg Spectral Count						PgSgFn / Pg Protein Intensity				
PGN	*q*-value	PgSgFn	Pg	log_2Ratio	log_2Sum	Regulation	Regulation	*q*-value	log_2Ratio	PgSgFn	Pg
PG1396	0.003529	70.37	109.5	-0.64	7.49	G	G	0.002046	-0.86	55243881	99260738
PGN0866	*mreB cell shape-determining protein MreB*										
PG1395	0.751373	2.13	3	-0.49	2.36	Y	Y	0.052108	-2.29	66413	1991744.5
PGN0867	*cell shape-determining protein MreC putative*										
PG1393	0.779998	2.13	2.5	-0.23	2.21	Y	Y	0.053629	-1.33	66413	532384.575
PGN0869	*penicillin-binding protein 2 putative*										
PG1389	0.031308	57.57	33.5	0.78	6.51	Y	Y	0.121255	0.03	82802254	63813474.6
PGN0872	*DNA-binding protein histone-like family*										
PG1388	0.058464	2.13	13	-2.61	3.92	Y	Y	0.026295	-5.36	66413	3526145.83
PGN0873	*hypothetical protein PG_1388*										
PG1387	0.492890	18.13	11	0.72	4.86	Y	Y	0.124354	-0.22	5246444.5	6634182.68
PGN0874	*hypothetical protein PG_1387*										
PG1386	0.542387	224.97	199	0.18	8.73	Y	Y	0.022204	-0.11	168161805	181174995
PGN0875	*gyrA DNA gyrase A subunit*										
PG1385	0.000000	713.28	379	0.91	10.09	R	R	0.001392	0.37	808318980	626400150
PGN0876	*TPR domain protein*										
PG1401	0.667335	843.36	851	-0.01	10.73	Y	G	0.003146	-0.52	921368767	1.3245e+09
PGN0880	*beta-eliminating lyase*										
PG1402	0.275068	2.13	11	-2.37	3.71	Y	G	0.001392	-6.43	66413	5725500.48
PGN0881	*AP endonuclease domain protein*										
PG1405	0.173614	2.13	12	-2.49	3.82	Y	G	0.000501	-5.88	66413	3868732.23
PGN0884	*hypothetical protein PG_1405*										
PG1407	0.751373	2.13	3	-0.49	2.36	Y	Y	0.053629	-1.30	66413	515156.975
PGN0885	*nitroimidazole resistance protein putative*										
PG1408	0.194509	9.6	10.5	-0.13	4.33	Y	Y	0.116286	-2.54	11582142	6892490
PGN0886	*heavy metal efflux pump CzcD family*										
PG1411	0.201724	7.46	16	-1.1	4.55	Y	Y	0.018743	-3.64	2934729	8272053.88
PGN0889	*potassium uptake protein TrkA putative*										
PG1414	0.000000	580.01	787	-0.44	10.42	G	Y	0.050220	-0.13	384631954	421114043
PGN0890	*hypothetical protein PG_1414*										
PG1416	0.700541	170.59	159	0.1	8.36	Y	Y	0.059515	-0.16	97579942.5	108819375
PGN0891	*fabK enoyl-(acyl-carrier-protein) reductase II*										
PG1417	0.010588	259.08	199	0.38	8.84	Y	Y	0.032546	0.15	117753136	106263044
PGN0893	*fumB fumarate hydratase class I anaerobic*										
PG1418	0.779998	20.26	18.5	0.13	5.28	Y	Y	0.011880	-0.44	5004032	6852551.5
PGN0894	*dnaX DNA polymerase III gamma and tau subunits*										
PG1421	0.001098	38.38	17	1.17	5.79	R	Y	0.151909	-0.03	13734869.5	14139549.1
PGN0895	*ferredoxin 4Fe-4S*										
PG1422	0.102560	2.13	13	-2.61	3.92	Y	G	0.000405	-5.84	66413	3762199.9
PGN0896	*dacB D-alanyl-D-alanine carboxypeptidase*										
PG1424	0.000419	981.96	1116.5	-0.19	11.04	G	Y	0.057050	-0.07	1.2313e+09	1.2954e+09
PGN0898	*peptidylarginine deiminase*										
PG1428	0.203808	2.13	11.5	-2.43	3.77	Y	G	0.000202	-6.71	66413	6872827.3
PGN0901	*ribH riboflavin synthase beta subunit*										
PG1430	0.700541	93.82	106	-0.18	7.64	Y	Y	0.015947	-0.32	129792532	161883241
PGN0902	*TPR domain protein*										
PG1431	0.122734	10.66	12	-0.17	4.5	Y	Y	0.011455	-3.35	683501	2863172.6
PGN0903	*DNA-binding response regulator LuxR family*										

TIGR	PgSgFn / Pg Spectral Count						PgSgFn / Pg Protein Intensity				
PGN	*q*-value	PgSgFn	Pg	log_2Ratio	log_2Sum	Regulation		*q*-value	log_2Ratio	PgSgFn	Pg
PG1432	0.000023	2.13	24	-3.49	4.71	Y	G	0.001555	-7.12	66413	9232851.25
PGN0904	*sensor histidine kinase*										
PG1065	0.000000	11.73	41	-1.81	5.72	G	Y	0.094130	-3.86	10074142.5	15535299
PGN0905	*pyrD dihydroorotate dehydrogenase*										
PG1064	0.000000	8.53	54.5	-2.68	5.98	G	Y	0.014040	-4.50	5506654.5	17953577.4
PGN0906	*dihydroorotate dehydrogenase putative*										
PG1063	0.543202	2.13	9	-2.08	3.48	Y	Y	0.011880	-5.43	66413	3098266.9
PGN0907	*transcriptional regulator putative*										
PG1214	0.630045	2.13	8	-1.91	3.34	Y	Y	0.019902	-4.94	66413	2407200.85
PGN0910	*hypothetical protein PG_1214*										
PG1213	0.400640	2.13	10	-2.23	3.6	Y	Y	0.038496	-5.15	66413	3971301.43
PGN0911	*rnhA ribonuclease H*										
PG1212	0.118209	25.59	42.5	-0.73	6.09	Y	Y	0.027753	-0.40	15294256.5	19930545.2
PGN0912	*TPR domain protein*										
PG1211	0.604563	2.13	4	-0.91	2.62	Y	Y	0.052108	-2.55	66413	2841627.1
PGN0913	*hexapeptide transferase family protein*										
PG1210	0.556958	601.33	599.5	0	10.23	Y	G	0.008485	-0.17	493265458	556661358
PGN0914	*peptidase M24 family*										
PG1209	0.771913	76.77	83	-0.11	7.32	Y	G	0.004244	-0.29	35974515.5	44077339.1
PGN0915	*hypothetical protein PG_1209*										
PG1208	0.000000	633.32	484	0.39	10.13	R	R	0.005157	0.25	452566348	381861529
PGN0916	*dnaK dnaK protein*										
PG1189	0.134175	323.06	271.5	0.25	9.22	Y	Y	0.099350	-0.08	254292213	267832009
PGN0935	*hypothetical protein PG_1189*										
PG1190	0.423387	2.13	9.5	-2.16	3.54	Y	Y	0.017881	-6.61	66413	7517930.65
PGN0936	*hprA glycerate dehydrogenase*										
PG1195	0.061251	7.46	18.5	-1.31	4.7	Y	Y	0.042801	-3.43	2825941.5	6105097.15
PGN0938	*bioF-2 8-amino-7-oxononanoate synthase*										
PG0992	0.584639	271.88	283.5	-0.06	9.12	Y	Y	0.157620	0.04	169480526	166238410
PGN0962	*thrS threonyl-tRNA synthetase*										
PG0991	0.620762	31.99	30.5	0.07	5.97	Y	Y	0.014886	-0.27	27529423	33052353.7
PGN0963	*infC translation initiation factor IF-3*										
PG0989	0.091810	91.69	64	0.52	7.28	Y	R	0.008263	0.70	68153035	42475717.4
PGN0965	*rplT ribosomal protein L20*										
PG0982	0.000032	10.66	34	-1.67	5.48	G	Y	0.041798	-4.27	8393158	19212681.3
PGN0972	*TPR domain protein*										
PG0980	0.543202	2.13	9	-2.08	3.48	Y	Y	0.013850	-4.18	66413	1318995.1
PGN0973	*hypothetical protein PG_0980*										
PG0978	0.021655	10.66	3	1.83	3.77	Y	Y	0.193910	0.94	1027023	1026842
PGN0974	*aroE shikimate 5-dehydrogenase*										
PG0977	0.600049	2.13	8.5	-2	3.41	Y	G	0.007923	-5.60	66413	3388627.18
PGN0975	*ubiE ubiquinone/menaquinone biosynthesis methyltransferase UbiE*										
PG0976	0.084508	118.35	87	0.44	7.68	Y	G	0.009001	-0.14	89572933.5	98466063.1
PGN0976	*phosphoribosylaminoimidazole-succinocarboxamide synthase putative*										
PG0975	0.604563	2.13	4	-0.91	2.62	Y	Y	0.052108	-2.39	66413	2270793.5
PGN0977	*PhoH family protein*										
PG0973	0.604563	2.13	4	-0.91	2.62	Y	Y	0.052108	-2.03	66413	1384770.58
PGN0980	*alpha-12-mannosidase family protein*										

TIGR	PgSgFn / Pg Spectral Count							PgSgFn / Pg Protein Intensity			
PGN	*q*-value	PgSgFn	Pg	log_2Ratio	log_2Sum	Regulation		*q*-value	log_2Ratio	PgSgFn	Pg
PG0969	0.779998	2.13	2.5	-0.23	2.21	Y	Y	0.053939	-1.24	66413	473251.925
PGN0981	*S-adenosylmethionine:tRNA ribosyltransferase-isomerase putative*										
PG0968	0.400640	2.13	10	-2.23	3.6	Y	G	0.008766	-5.44	66413	3036536.2
PGN0982	*mrr Mrr restriction system protein*										
PG0965	0.584639	18.13	26.5	-0.55	5.48	Y	Y	0.055138	-0.32	7595643.5	9551632.15
PGN0984	*phosphatidylserine decarboxylase-related protein*										
PG0962	0.007276	81.03	115	-0.51	7.61	G	Y	0.018791	-0.65	46341427	71133568.5
PGN0987	*proS prolyl-tRNA synthetase*										
PG0960	0.751373	2.13	3	-0.49	2.36	Y	Y	0.052108	-2.35	66413	2155144.4
PGN0989	*conserved hypothetical protein*										
PG0959	0.557702	42.65	32.5	0.39	6.23	Y	Y	0.026150	-0.56	18247372	26636653.7
PGN0990	*mrp ATP-binding protein Mrp/Nbp35 family*										
PG0958	0.692989	2.13	3.5	-0.72	2.49	Y	Y	0.052108	-2.41	66413	2352081.83
PGN0991	*ribonuclease BN putative*										
PG0957	0.667335	2.13	8	-1.91	3.34	Y	G	0.002081	-6.27	66413	5138358.6
PGN0992	*ribF riboflavin biosynthesis protein RibF*										
PG0956	0.779998	2.13	2.5	-0.23	2.21	Y	Y	0.053020	-1.41	66413	598069.675
PGN0993	*peptidase M23/M37 family putative*										
PG0955	0.346258	14.93	25	-0.74	5.32	Y	Y	0.138569	-0.58	7792279.5	5868613.65
PGN0994	*hypothetical protein PG_0955*										
PG0954	0.108176	9.6	18.5	-0.95	4.81	Y	Y	0.019452	-3.83	2453646	9094745.65
PGN0996	*TPR domain protein*										
PG0953	0.134175	2.13	12.5	-2.55	3.87	Y	G	0.000532	-7.14	66413	9302678.5
PGN0997	*dut deoxyuridine 5'-triphosphate nucleotidohydrolase*										
PG0952	0.770505	37.32	34.5	0.11	6.17	Y	G	0.007622	-0.62	11877208	18409027.8
PGN0998	*ispG 1-hydroxy-2-methyl-2-(E)-butenyl 4-diphosphate synthase*										
PG0951	0.089835	39.45	58	-0.56	6.61	Y	Y	0.034467	-0.73	24374430	37065505.6
PGN0999	*purE phosphoribosylaminoimidazole carboxylase PurE protein*										
PG0950	0.000086	108.75	61.5	0.82	7.41	R	R	0.009001	0.84	112255992	62210921.2
PGN1000	*gcvH glycine cleavage system H protein*										
PG0949	0.784897	2.13	5.5	-1.37	2.93	Y	Y	0.013019	-4.27	66413	1398010.73
PGN1001	*conserved hypothetical protein*										
PG0948	0.039315	14.93	7.5	0.99	4.49	Y	Y	0.129674	-1.76	3921185	2641016.23
PGN1002	*AMP nucleosidase putative*										
PG0946	0.779998	28.79	28	0.04	5.83	Y	Y	0.115558	-0.07	26987424.5	28487792
PGN1004	*ABC transporter ATP-binding protein*										
PG0945	0.483206	38.38	44.5	-0.21	6.37	Y	Y	0.170519	-0.09	36520670.5	37685636.8
PGN1005	*ABC transporter permease protein putative*										
PG0938	0.779998	2.13	2.5	-0.23	2.21	Y	Y	0.052842	-1.50	66413	672041.375
PGN1009	*calcium-transporting ATPase*										
PG0937	0.029147	66.1	40	0.72	6.73	Y	R	0.002521	0.80	43572376	24992891.5
PGN1010	*hypothetical protein PG_0937*										
PG0936	0.542387	8.53	7	0.29	3.96	Y	Y	0.046188	-3.77	3806837	12511851.5
PGN1011	*xanthine/uracil permease family protein*										
PG0935	0.751373	2.13	3	-0.49	2.36	Y	Y	0.052108	-1.76	66413	956026.5
PGN1012	*ispE 4-diphosphocytidyl-2C-methyl-D-erythritol kinase*										
PG0933	0.000000	1643	1363	0.27	11.55	R	Y	0.127626	0.02	1.5061e+09	1.4879e+09
PGN1014	*translation elongation factor G putative*										

TIGR	PgSgFn / Pg Spectral Count							PgSgFn / Pg Protein Intensity			
PGN	*q*-value	PgSgFn	Pg	log_2Ratio	log_2Sum	Regulation		*q*-value	log_2Ratio	PgSgFn	Pg
PG0932	0.102059	10.66	18.5	-0.8	4.87	Y	Y	0.062174	-2.87	2207452.5	5116538.38
PGN1015	*DNA polymerase III delta prime subunit putative*										
PG0928	0.639746	110.88	126	-0.18	7.89	Y	Y	0.038811	-0.26	75065764	88694803.1
PGN1019	*response regulator*										
PG0924	0.014718	2.13	16	-2.91	4.18	Y	G	0.001039	-6.37	66413	5480082.08
PGN1023	*5'-nucleotidase lipoprotein e(P4) family*										
PG0923	0.770282	23.46	19	0.3	5.41	Y	Y	0.014081	0.67	20451408	12686598.4
PGN1024	*rbfA ribosome-binding factor A*										
PG0920	0.000000	2.13	42.5	-4.32	5.48	Y	G	0.000986	-7.36	66413	10846646.6
PGN1026	*glycosyl transferase group 2 family protein*										
PG0919	0.000188	23.46	53	-1.18	6.26	G	G	0.001582	-1.13	10737889.5	23193936.3
PGN1027	*pyrC dihydroorotase*										
PG0918	0.543202	2.13	9	-2.08	3.48	Y	Y	0.014536	-5.25	66413	2817060
PGN1028	*hypothetical protein PG_0918*										
PG0914	0.014629	2.13	16	-2.91	4.18	Y	G	0.001540	-7.20	66413	9718261.65
PGN1032	*hypothetical protein PG_0914*										
PG0912	0.600049	2.13	4	-0.91	2.62	Y	Y	0.052108	-1.91	66413	1543373
PGN1033	*polysaccharide transport protein putative*										
PG0910	0.685422	2.13	3.5	-0.72	2.49	Y	Y	0.052147	-1.57	66413	965817
PGN1034	*FHA domain protein*										
PG0909	0.000015	9.6	34.5	-1.85	5.46	G	G	0.009741	-4.40	3519746	15716969.7
PGN1035	*conserved hypothetical protein*										
PG0906	0.000000	81.03	23	1.82	6.7	R	R	0.003547	1.32	178298385	71494042.4
PGN1037	*lipoprotein putative*										
PG0903	0.250677	11.73	14	-0.26	4.69	Y	Y	0.050595	-3.64	5807400.5	11711224.4
PGN1038	*conserved hypothetical protein*										
PG0900	0.685422	2.13	3.5	-0.72	2.49	Y	Y	0.052108	-1.72	66413	1185331.5
PGN1041	*cydA cytochrome d ubiquinol oxidase subunit I*										
PG0899	0.608229	2.13	8.5	-2	3.41	Y	Y	0.018743	-2.37	66413	380729.5
PGN1042	*cydB cytochrome d ubiquinol oxidase subunit II*										
PG0898	0.336653	2.13	10.5	-2.3	3.66	Y	G	0.000107	-6.24	66413	4960338.25
PGN1043	*conserved hypothetical protein*										
PG0897	0.604563	2.13	4	-0.91	2.62	Y	Y	0.063017	-0.69	66413	226326
PGN1044	*alpha-amylase family protein*										
PG0894	0.017364	8.53	3	1.51	3.53	Y	Y	0.056249	1.89	897296	149871.45
PGN1046	*radC DNA repair protein RadC*										
PG0890	0.201724	157.8	188.5	-0.26	8.44	Y	G	0.007304	-0.58	98366496	146850067
PGN1049	*alkaline phosphatase putative*										
PG0889	0.627286	30.92	37	-0.26	6.09	Y	Y	0.013957	-0.71	15293668	24973847.1
PGN1050	*peptidase M24 family*										
PG0886	0.457936	2.13	9.5	-2.16	3.54	Y	G	0.000670	-5.37	66413	2726739.23
PGN1052	*hypothetical protein PG_0886*										
PG0885	0.000000	2.13	34.5	-4.02	5.19	Y	G	0.000770	-6.84	66413	7537068.63
PGN1053	*phospho-2-dehydro-3-deoxyheptonate aldolase/chorismate mutase*										
PG0884	0.423387	2.13	9.5	-2.16	3.54	Y	G	0.001865	-4.85	66413	1917166.35
PGN1054	*hypothetical protein PG_0884*										
PG0883	0.246190	7.46	14	-0.91	4.42	Y	Y	0.018904	-3.34	1192147	3772053.85
PGN1055	*hypothetical protein PG_0883*										

TIGR	PgSgFn / Pg Spectral Count							PgSgFn / Pg Protein Intensity			
PGN	*q*-value	PgSgFn	Pg	log_2Ratio	log_2Sum	Regulation		*q*-value	log_2Ratio	PgSgFn	Pg
PG0881	0.002122	173.79	232.5	-0.42	8.67	G	Y	0.170519	0.04	150443196	147736133
PGN1057	*recA recA protein*										
PG0880	0.786857	26.65	27.5	-0.05	5.76	Y	Y	0.134333	-0.13	13621843.5	14594895.9
PGN1058	*bcp bacterioferritin comigratory protein*										
PG0877	0.751373	2.13	3	-0.49	2.36	Y	Y	0.052108	-3.36	66413	11415687.5
PGN1061	*hypothetical protein PG_0877*										
PG0876	0.003155	2.13	18	-3.08	4.33	Y	G	0.004701	-6.16	66413	4837485.98
PGN1062	*thdF thiophene and furan oxidation protein ThdF*										
PG2119	0.000000	2.13	36	-4.08	5.25	Y	G	0.006764	-8.35	66413	22495039.5
PGN1068	*oxidoreductase Gfo/Idh/MocA family*										
PG1288	0.486470	42.65	56	-0.39	6.62	Y	Y	0.125479	-0.30	43986317	51066800.3
PGN1078	*gmd GDP-mannose 46-dehydratase*										
PG1289	0.000746	11.73	30	-1.35	5.38	G	G	0.000238	-5.37	578959	11865696.6
PGN1079	*fcl GDP-fucose synthetase*										
PG1290	0.000003	987.29	1172.5	-0.25	11.08	G	G	0.001449	-0.24	908683287	1.0702e+09
PGN1080	*ilvE branched-chain amino acid aminotransferase*										
PG1291	0.687127	29.85	36	-0.27	6.04	Y	Y	0.143269	-0.01	17319184	17511025.5
PGN1081	*hypothetical protein PG_1291*										
PG1294	0.659176	36.25	45.5	-0.33	6.35	Y	Y	0.116286	-0.10	15099444.5	16009417.8
PGN1085	*feoB-2 ferrous iron transport protein B*										
PG1296	0.659176	2.13	8	-1.91	3.34	Y	G	0.006461	-5.64	66413	3429830.45
PGN1087	*hypothetical protein PG_1296*										
PG1297	0.001760	548.02	480.5	0.19	10.01	R	Y	0.178380	-0.01	503860624	507873671
PGN1088	*rpsA ribosomal protein S1*										
PG1300	0.403113	2.13	10	-2.23	3.6	Y	Y	0.019334	-2.30	66413	362616.35
PGN1089	*conserved hypothetical protein*										
PG1302	0.064532	218.57	173	0.34	8.61	Y	G	0.009931	-0.15	302782184	334996553
PGN1091	*hypothetical protein PG_1302*										
PG1304	0.600049	21.32	19	0.17	5.33	Y	Y	0.052192	1.01	8335892	3282121.8
PGN1093	*hypothetical protein PG_1304*										
PG1305	0.000417	762.32	642	0.25	10.46	R	G	0.006544	-0.29	719204440	879828428
PGN1094	*gcvP glycine cleavage system P protein*										
PG1306	0.751373	2.13	3	-0.49	2.36	Y	Y	0.052108	-2.10	66413	1530352.53
PGN1095	*metallo-beta-lactamase family protein*										
PG1307	0.013571	2.13	15.5	-2.86	4.14	Y	G	0.000602	-6.24	66413	4985773.13
PGN1096	*gidB glucose-inhibited division protein B*										
PG1308	0.262727	30.92	42	-0.44	6.19	Y	G	0.003984	-0.30	12577367	15487200.1
PGN1097	*hypothetical protein PG_1308*										
PG1310	0.784897	2.13	5.5	-1.37	2.93	Y	G	0.001895	-5.33	66413	2682120.18
PGN1098	*exsB protein*										
PG1311	0.784897	2.13	5.5	-1.37	2.93	Y	G	0.007874	-3.79	66413	956828
PGN1099	*conserved hypothetical protein*										
PG1313	0.000000	286.8	462	-0.69	9.55	G	Y	0.047737	-0.09	363300507	385514802
PGN1103	*dipeptidase-related protein*										
PG1314	0.751373	2.13	3	-0.49	2.36	Y	Y	0.052485	-1.47	66413	839419.5
PGN1104	*aroC chorismate synthase*										
PG1315	0.003657	87.43	100.5	-0.2	7.55	G	G	0.004349	-0.71	58987496.5	96993805
PGN1105	*slyD peptidyl-prolyl cis-trans isomerase SlyD FKBP-type*										

TIGR	PGN	Description	PgSgFn / Pg Spectral Count					Regulation		PgSgFn / Pg Protein Intensity			
			q-value	PgSgFn	Pg	log_2Ratio	log_2Sum			*q*-value	log_2Ratio	PgSgFn	Pg
PG1317	PGN1107	hypothetical protein PG_1317	0.225676	2.13	6	-1.49	3.02	Y	Y	0.083272	-0.16	66413	143841.5
PG1318	PGN1108	RNA polymerase sigma-70 factor ECF subfamily	0.779998	2.13	2.5	-0.23	2.21	Y	Y	0.052108	-2.01	66413	1760533
PG1321	PGN1111	fhs formate--tetrahydrofolate ligase	0.000000	830.56	387.5	1.1	10.25	R	R	0.001053	0.35	283146008	221867547
PG1323	PGN1112	PhoH family protein	0.000000	249.49	142.5	0.81	8.61	R	Y	0.015038	0.35	149744341	117348989
PG1325	PGN1114	hypothetical protein PG_1325	0.111805	10.66	12	-0.17	4.5	Y	Y	0.102207	-1.83	675197	1010994.5
PG1326	PGN1115	hemagglutinin putative	0.358444	6.4	12.5	-0.97	4.24	Y	Y	0.069876	-3.77	7060092	12212055.3
PG1327	PGN1116	aminotransferase putative	0.295572	458.46	410.5	0.16	9.76	Y	Y	0.019334	-0.35	345527990	435999988
PG1328	PGN1117	CoA ligase family protein	0.000015	364.64	470.5	-0.37	9.71	G	Y	0.115558	-0.05	244013478	252557097
PG1330	PGN1119	mscL large conductance mechanosensitive channel protein	0.000000	2.13	49	-4.52	5.68	Y	G	0.000137	-9.41	66413	44792325.5
PG1332	PGN1122	pntB NAD(P) transhydrogenase beta subunit	0.268678	59.71	42	0.51	6.67	Y	Y	0.031171	0.62	50661605.5	31964946.5
PG1333	PGN1123	hypothetical protein PG_1333	0.608229	2.13	8.5	-2	3.41	Y	G	0.000107	-5.19	66413	2403697.9
PG1334	PGN1124	band 7/Mec-2 family protein	0.659176	183.38	165	0.15	8.44	Y	G	0.000844	-0.15	84409401	93349815
PG1335	PGN1125	membrane protein putative	0.630464	29.85	22.5	0.41	5.71	Y	Y	0.052108	-0.18	28893674	32888584
PG1340	PGN1128	L-lactate permease	0.274290	2.13	11	-2.37	3.71	Y	G	0.000986	-7.15	66413	9377922.63
PG1341	PGN1129	hypothetical protein PG_1341	0.000000	349.71	146.5	1.26	8.95	R	R	0.003984	0.39	610086858	467010261
PG1342	PGN1130	murB UDP-N-acetylenolpyruvoylglucosamine reductase	0.667335	2.13	8	-1.91	3.34	Y	G	0.000240	-5.96	66413	4084969.3
PG1343	PGN1131	lipB lipoate-protein ligase B	0.102154	2.13	13	-2.61	3.92	Y	Y	0.014353	-4.63	66413	1822692.25
PG1345	PGN1134	glycosyl transferase group 1 family protein	0.014629	2.13	16	-2.91	4.18	Y	G	0.002909	-6.43	66413	5781385.75
PG1346	PGN1135	glycosyl transferase group 1 family protein	0.600049	2.13	4	-0.91	2.62	Y	Y	0.052108	-1.69	66413	1127975.5
PG1347	PGN1136	conserved hypothetical protein	0.009829	7.46	23	-1.62	4.93	G	G	0.009241	-4.33	4496819	16558067
PG1348	PGN1137	conserved hypothetical protein TIGR00147	0.050334	2.13	14	-2.72	4.01	Y	G	0.003885	-8.02	66413	17538637.4
PG1351	PGN1138	hypothetical protein PG_1351	0.785443	2.13	5	-1.23	2.83	Y	G	0.002953	-5.05	66413	2213652.2
PG1352	PGN1139	hypothetical protein PG_1352	0.667335	19.19	22	-0.2	5.36	Y	Y	0.013019	-0.84	8660602.5	15658701.7
PG1353	PGN1140	pyrE orotate phosphoribosyltransferase	0.000000	275.08	148.5	0.89	8.73	R	R	0.000484	1.08	220405827	104151658

TIGR	PGN	Description	PgSgFn / Pg Spectral Count						PgSgFn / Pg Protein Intensity				
			q-value	PgSgFn	Pg	log_2Ratio	log_2Sum	Regulation	Regulation	q-value	log_2Ratio	PgSgFn	Pg
PG1354	PGN1141	hydrolase carbon-nitrogen family	0.040076	2.13	14.5	-2.77	4.06	Y	Y	0.021356	-6.65	66413	8070274.23
PG1355	PGN1142	acyltransferase putative	0.773021	2.13	6.5	-1.61	3.11	Y	G	0.002484	-4.42	66413	1427814.3
PG1356	PGN1143	hypothetical protein PG_1356	0.699805	101.29	113.5	-0.16	7.75	Y	Y	0.090202	-0.13	62792796.5	68109291.7
PG1360	PGN1148	purD phosphoribosylamine--glycine ligase	0.659176	123.68	134	-0.12	8.01	Y	Y	0.062627	-0.18	110758065	125086175
PG1361	PGN1149	dipeptidyl aminopeptidase IV putative	0.284765	233.5	264.5	-0.18	8.96	Y	Y	0.026295	-0.25	152036286	180880040
PG1362	PGN1150	conserved hypothetical protein	0.000512	22.39	46.5	-1.05	6.11	G	G	0.009620	-1.03	9265715.5	19214108.1
PG1364	PGN1151	dxr 1-deoxy-D-xylulose 5-phosphate reductoisomerase	0.548692	30.92	21.5	0.52	5.71	Y	Y	0.023889	-0.94	7684901.5	15503143.6
PG1365	PGN1152	16S rRNA processing protein RimM putative	0.751373	2.13	3	-0.49	2.36	Y	Y	0.052108	-1.71	66413	1167431
PG1366	PGN1153	murA UDP-N-acetylglucosamine 1-carboxyvinyltransferase	0.169098	141.8	164	-0.21	8.26	Y	G	0.000803	-0.65	32635361	51082156.6
PG1367	PGN1154	hypothetical protein PG_1367	0.028897	7.46	21	-1.49	4.83	Y	G	0.009727	-3.96	2829952.5	10156123.6
PG1368	PGN1155	pgi glucose-6-phosphate isomerase	0.003827	52.24	85.5	-0.71	7.11	G	G	0.004329	-0.61	18710557	28638528.9
PG1369	PGN1156	gpsA glycerol-3-phosphate dehydrogenase (NAD(P)+)	0.000000	2.13	28.5	-3.74	4.94	Y	G	0.002493	-7.03	66413	8728978.78
PG1370	PGN1157	lysS lysyl-tRNA synthetase	0.446932	108.75	128.5	-0.24	7.89	Y	Y	0.161524	0.04	75827745	73752341.8
PG1371	PGN1158	phosphorylase family protein	0.000191	43.71	80.5	-0.88	6.96	G	Y	0.028201	0.50	57764571	39922682.6
PG1372	PGN1159	hypothetical protein PG_1372	0.751373	53.31	46	0.21	6.63	Y	Y	0.146675	-0.07	14490860	15146007.4
PG1066	PGN1162	ctfA butyrate-acetoacetate CoA-transferase subunit A	0.250218	66.1	73.5	-0.15	7.13	Y	G	0.006088	-0.29	106386753	130060537
PG1067	PGN1163	conserved hypothetical protein	0.480701	53.31	40.5	0.4	6.55	Y	Y	0.067266	-0.04	42117021	43239949.5
PG1068	PGN1164	conserved hypothetical protein	0.059089	243.09	240	0.02	8.92	Y	G	0.001290	-0.22	166349357	193539906
PG1069	PGN1165	alcohol dehydrogenase zinc-containing putative	0.545730	341.18	321.5	0.09	9.37	Y	R	0.001266	0.61	386731407	253182320
PG1070	PGN1166	kamA L-lysine 23-aminomutase	0.213253	106.62	134.5	-0.34	7.91	Y	Y	0.026780	-0.22	90756405.5	105772351
PG1072	PGN1168	MutS family protein	0.203808	2.13	11.5	-2.43	3.77	Y	G	0.007211	-5.01	66413	2221930.85
PG1073	PGN1169	kamD D-lysine 56-aminomutase alpha subunit	0.192280	56.51	45	0.33	6.67	Y	Y	0.022832	0.82	53020325.5	29228979.6
PG1074	PGN1170	kamE D-lysine 56-aminomutase beta subunit	0.290677	58.64	75.5	-0.36	7.07	Y	Y	0.113888	-0.04	29335040	30164746.2
PG1075	PGN1171	coenzyme A transferase beta subunit	0.010250	36.25	61	-0.75	6.6	Y	Y	0.014416	-1.08	10880808	21077514.2

TIGR	PgSgFn / Pg Spectral Count					Regulation		PgSgFn / Pg Protein Intensity			
PGN	q-value	PgSgFn	Pg	log_2Ratio	log_2Sum			q-value	log_2Ratio	PgSgFn	Pg
PG1076	0.779998	732.47	746.5	-0.03	10.53	Y	G	0.005860	-0.50	613715767	868910725
PGN1172	acdA acyl-CoA dehydrogenase short-chain specific										
PG1077	0.000000	788.98	597.5	0.4	10.44	R	R	0.003984	0.33	984545854	785671469
PGN1173	etfB-2 electron transfer flavoprotein beta subunit										
PG1078	0.000000	1973.52	1603	0.3	11.8	R	Y	0.026150	0.18	959478515	842193984
PGN1174	etfA-2 electron transfer flavoprotein alpha subunit										
PG1079	0.176563	31.99	48.5	-0.6	6.33	Y	Y	0.012037	-0.41	28485202.5	37641287.4
PGN1175	enoyl-CoA hydratase/isomerase family protein										
PG1080	0.290104	149.27	141.5	0.08	8.18	Y	Y	0.155528	-0.03	70265061.5	72724313.7
PGN1176	3-hydroxyacyl-CoA dehydrogenase family protein										
PG1081	0.000000	198.31	314	-0.66	9	G	G	0.002376	-0.56	247954562	366385874
PGN1178	ackA acetate kinase										
PG1082	0.025944	574.68	503.5	0.19	10.07	Y	R	0.003925	0.35	701101500	549727427
PGN1179	pta phosphotransacetylase										
PG1084	0.000000	834.83	1022.5	-0.29	10.86	G	Y	0.026630	0.09	696961665	654126966
PGN1181	thioredoxin family protein										
PG1085	0.000055	179.12	125	0.52	8.25	R	Y	0.115558	0.06	515691103	495001953
PGN1182	hypothetical protein PG_1085										
PG1089	0.000000	247.36	135	0.87	8.58	R	Y	0.032184	0.21	158878309	136647609
PGN1186	rprY DNA-binding response regulator RprY										
PG1091	0.000491	2.13	20.5	-3.27	4.5	Y	G	0.000009	-6.15	66413	4679378.83
PGN1187	DHH subfamily 1 protein										
PG1093	0.692989	34.12	29.5	0.21	5.99	Y	Y	0.029655	0.38	37000849	28101221.9
PGN1188	hypothetical protein PG_1093										
PG1094	0.550186	102.35	117	-0.19	7.78	Y	Y	0.052192	0.15	105695235	95424078.3
PGN1189	pgm phosphomannomutase										
PG1095	0.024693	2.13	15	-2.82	4.1	Y	G	0.002953	-6.22	66413	4988817.63
PGN1190	RNA methyltransferase TrmA family										
PG1097	0.014718	2.13	16	-2.91	4.18	Y	G	0.000137	-6.02	66413	4265644.75
PGN1194	Mur ligase domain protein/alanine racemase										
PG1098	0.225676	2.13	6	-1.49	3.02	Y	Y	0.052192	-1.49	66413	867405
PGN1195	hypothetical protein PG_1098										
PG1100	0.751373	2.13	3	-0.49	2.36	Y	Y	0.052108	-2.43	66413	2395930.5
PGN1197	hypothetical protein PG_1100										
PG1101	0.751373	2.13	3	-0.49	2.36	Y	Y	0.096757	-0.00	66413	118326
PGN1198	sodium:solute symporter family protein										
PG1497	0.600049	2.13	4	-0.91	2.62	Y	Y	0.076556	-0.25	66413	162822
PGN1199	DNA-binding protein histone-like family										
PG1103	0.000000	2.13	29	-3.77	4.96	Y	G	0.000044	-6.25	66413	4984157.75
PGN1200	ATPase AAA family										
PG1105	0.000479	2.13	20.5	-3.27	4.5	Y	G	0.001531	-6.35	66413	5423368.3
PGN1202	rpoN RNA polymerase sigma-54 factor										
PG1106	0.000336	2.13	21	-3.3	4.53	Y	G	0.000674	-6.62	66413	6494269.75
PGN1203	murF UDP-N-acetylmuramoylalanyl-D-glutamyl-26-diaminopimelate--D-alanyl-D-alanyl ligase										
PG1114	0.728237	21.32	18	0.24	5.3	Y	Y	0.120505	-0.11	19503493.5	21903176
PGN1204	panD aspartate-1-decarboxylase										
PG1115	0.751373	82.1	81	0.02	7.35	Y	Y	0.024051	-0.39	45951884.5	59972323.3
PGN1205	ffh signal recognition particle protein										

TIGR	PgSgFn / Pg Spectral Count					Regulation		PgSgFn / Pg Protein Intensity			
PGN	*q*-value	PgSgFn	Pg	log_2Ratio	log_2Sum			*q*-value	log_2Ratio	PgSgFn	Pg
PG1116	0.010468	226.03	175.5	0.37	8.65	Y	Y	0.059025	0.07	234967495	223816059
PGN1206	*folD methylenetetrahydrofolate dehydrogenase/methenyltetrahydrofolate cyclohydrolase*										
PG1118	0.440970	105.55	119	-0.17	7.81	Y	Y	0.050595	-0.30	56284204	70224153.2
PGN1208	*clpB clpB protein*										
PG1119	0.679564	71.43	79.5	-0.15	7.24	Y	Y	0.103945	-0.11	36751946	39331855.1
PGN1209	*flavodoxin putative*										
PG1121	0.659176	89.56	103	-0.2	7.59	Y	Y	0.155528	0.05	83350792	79919269.4
PGN1218	*asnS asparaginyl-tRNA synthetase*										
PG1123	0.000000	88.49	161	-0.86	7.96	G	G	0.009931	-0.41	54793411	72603315
PGN1220	*purB adenylosuccinate lyase*										
PG1124	0.283063	9.6	15.5	-0.69	4.65	Y	Y	0.026630	-3.91	5318754.5	13502525.9
PGN1221	*ATP:cob(I)alamin adenosyltransferase putative*										
PG1126	0.045570	33.05	16.5	1	5.63	Y	R	0.004704	0.33	10794856.5	8565584.88
PGN1223	*uraA uracil permease*										
PG1127	0.076002	2.13	13.5	-2.66	3.97	Y	G	0.000137	-6.35	66413	5345478.48
PGN1224	*transcriptional regulator AsnC Family*										
PG1129	0.001330	129.01	185	-0.52	8.29	G	Y	0.100194	-0.09	67433023.5	72035407.6
PGN1226	*nrd ribonucleotide reductase*										
PG1130	0.608229	2.13	8.5	-2	3.41	Y	G	0.000327	-5.42	66413	2825666.68
PGN1227	*TPR domain protein*										
PG1132	0.295346	159.93	136	0.23	8.21	Y	G	0.009001	-0.42	89095806	118504092
PGN1229	*valS valyl-tRNA synthetase*										
PG1133	0.768073	19.19	24	-0.32	5.43	Y	G	0.000462	-1.08	8846350	18701410.1
PGN1230	*hypothetical protein PG_1133*										
PG1134	0.000000	107.69	186.5	-0.79	8.2	G	Y	0.158885	0.14	105484992	100245873
PGN1232	*trxB thioredoxin reductase*										
PG1135	0.751373	13.86	18	-0.38	4.99	Y	Y	0.015333	-0.81	4819048.5	8619352.18
PGN1233	*bacterial sugar transferase*										
PG1136	0.475857	2.13	9	-2.08	3.48	Y	G	0.003580	-4.97	66413	2097187.5
PGN1234	*conserved hypothetical protein*										
PG1138	0.000141	2.13	22	-3.37	4.59	Y	G	0.000137	-6.31	66413	5233125.1
PGN1236	*porR pigmentation and extracellular proteinase regulator*										
PG1139	0.620488	26.65	30	-0.17	5.82	Y	Y	0.137374	0.07	19109323.5	16411510.2
PGN1238	*hypothetical protein PG_1139*										
PG1140	0.608229	2.13	8.5	-2	3.41	Y	G	0.000238	-5.63	66413	3260432.65
PGN1239	*glycosyl transferase group 2 family protein*										
PG1141	0.756785	19.19	22	-0.2	5.36	Y	Y	0.028659	-0.68	6198220.5	9457935.3
PGN1240	*glycosyl transferase group 1 family protein*										
PG1143	0.001408	8.53	26	-1.61	5.11	G	Y	0.014837	-4.23	3899241	14799113.8
PGN1243	*sugar dehydrogenase UDP-glucose/GDP-mannose dehydrogenase family*										
PG1145	0.535064	23.46	33.5	-0.51	5.83	Y	G	0.007304	-0.69	10874237	17671176.7
PGN1245	*long-chain-fatty-acid--CoA ligase putative*										
PG1149	0.659176	23.46	27	-0.2	5.66	Y	Y	0.065448	0.69	21924902.5	10778140.1
PGN1251	*glycosyl transferase group 1 family protein*										
PG1151	0.012172	55.44	85.5	-0.62	7.14	Y	Y	0.015803	-0.21	33411549	38669237.1
PGN1252	*alcohol dehydrogenase iron-containing*										
PG1155	0.751373	2.13	3	-0.49	2.36	Y	Y	0.052108	-2.23	66413	2401366.5
PGN1255	*ADP-heptose--LPS heptosyltransferase putative*										

TIGR / *PGN*	PgSgFn / Pg Spectral Count *q*-value	PgSgFn	Pg	log_2Ratio	log_2Sum	Regulation		PgSgFn / Pg Protein Intensity *q*-value	log_2Ratio	PgSgFn	Pg
PG1156	0.274290	2.13	11	-2.37	3.71	Y	G	0.000271	-5.62	66413	3235642.5
PGN1256	*S4 domain protein*										
PG1162	0.543202	2.13	9	-2.08	3.48	Y	Y	0.025232	-3.18	66413	732623
PGN1261	*ATP:cob(I)alamin adenosyltransferase putative*										
PG1163	0.115710	2.13	12.5	-2.55	3.87	Y	Y	0.014416	-4.46	66413	1619780
PGN1262	*cbiA cobyrinic acid ac-diamide synthase*										
PG1164	0.000026	43.71	87	-0.99	7.03	G	Y	0.010329	-0.93	17895463.5	32968922.2
PGN1263	*hypothetical protein PG_1164*										
PG1170	0.751373	2.13	3	-0.49	2.36	Y	Y	0.052108	-2.37	66413	2912041
PGN1267	*SerB family protein*										
PG1171	0.000000	45.85	92	-1	7.11	G	Y	0.010571	-1.46	16693903.5	38942831.3
PGN1268	*oxidoreductase putative*										
PG1172	0.000001	105.55	53	0.99	7.31	R	R	0.001675	1.36	66856963.5	26374132.3
PGN1269	*iron-sulfur cluster binding protein putative*										
PG1173	0.647578	25.59	20	0.36	5.51	Y	G	0.009241	-0.38	14378200.5	18741508.7
PGN1270	*YkgG family protein*										
PG1174	0.000037	2.13	23.5	-3.46	4.68	Y	G	0.000390	-6.54	66413	6114505.58
PGN1271	*thioesterase family protein*										
PG2188	0.188231	111.95	141	-0.33	7.98	Y	Y	0.161524	-0.08	69076606.5	71504450.5
PGN1272	*lysA diaminopimelate decarboxylase*										
PG1058	0.139125	6.4	16	-1.32	4.49	Y	G	0.004568	-4.40	1938503.5	9821825.13
PGN1296	*OmpA family protein*										
PG1056	0.357095	7.46	14.5	-0.96	4.46	Y	Y	0.028575	-3.26	2227780.5	5618563.5
PGN1298	*conserved hypothetical protein*										
PG1052	0.400806	2.13	5	-1.23	2.83	Y	Y	0.052108	-2.06	66413	1448884.5
PGN1301	*transcriptional regulator putative*										
PG1050	0.012070	2.13	16	-2.91	4.18	Y	G	0.000390	-5.86	66413	3831161.6
PGN1303	*hypothetical protein PG_1050*										
PG1049	0.102059	26.65	44.5	-0.74	6.15	Y	G	0.002063	-1.00	13636023	26904687.7
PGN1304	*conserved hypothetical protein*										
PG1044	0.398768	2.13	10	-2.23	3.6	Y	G	0.002617	-5.59	66413	3213807.85
PGN1308	*iron dependent repressor putative*										
PG1043	0.751373	2.13	3	-0.49	2.36	Y	Y	0.052842	-1.49	66413	663401
PGN1309	*feoB-1 ferrous iron transport protein B*										
PG1039	0.278503	29.85	44.5	-0.58	6.22	Y	G	0.000584	-1.06	10049993.5	21015456.1
PGN1313	*integral membrane protein*										
PG1038	0.751373	2.13	3	-0.49	2.36	Y	Y	0.052108	-1.95	66413	1247991.83
PGN1314	*ATP-dependent DNA helicase UvrD/PcrA/Rep Family*										
PG1037	0.014629	397.69	340.5	0.22	9.53	Y	Y	0.018412	0.16	351891467	315887549
PGN1315	*hypothetical protein PG_1037*										
PG1036	0.017413	10.66	23.5	-1.14	5.09	Y	G	0.007304	-4.72	628299	8276260.65
PGN1316	*uvrA-1 excinuclease ABC A subunit*										
PG1035	0.773318	20.26	23	-0.18	5.43	Y	Y	0.014221	-0.70	8621698	13566718.8
PGN1317	*hypothetical protein PG_1035*										
PG1034	0.286742	8.53	9.5	-0.16	4.17	Y	Y	0.130581	-2.72	2811217.5	3709027.53
PGN1318	*ABC transporter ATP-binding protein*										
PG1030	0.580738	30.92	40.5	-0.39	6.16	Y	Y	0.022094	-0.43	23253968.5	30875104.1
PGN1321	*hypothetical protein PG_1030*										

TIGR	PGN		PgSgFn / Pg Spectral Count					Regulation		PgSgFn / Pg Protein Intensity			
			q-value	PgSgFn	Pg	log_2Ratio	log_2Sum			*q*-value	log_2Ratio	PgSgFn	Pg
PG1028	PGN1323	*TPR domain protein*	0.014718	509.64	580	-0.19	10.09	Y	Y	0.056585	-0.07	300942842	316617805
PG0685	PGN1329	*ABC transporter ATP-binding protein*	0.000486	2.13	20.5	-3.27	4.5	Y	G	0.000592	-6.78	66413	7228712.28
PG0628	PGN1330	*ABC transporter ATP-binding protein*	0.000000	12.79	43.5	-1.77	5.81	G	Y	0.048991	-3.60	5237538	10840225.5
PG1022	PGN1334	*hypothetical protein PG_1022*	0.013053	11.73	24.5	-1.06	5.18	Y	Y	0.188417	-2.86	8483454.5	8238190.23
PG1020	PGN1335	*hypothetical protein PG_1020*	0.000000	2.13	28	-3.72	4.91	Y	G	0.005239	-7.16	66413	9738203.35
PG1019	PGN1336	*lipoprotein putative*	0.779998	18.13	20	-0.14	5.25	Y	G	0.003984	-0.82	5560293	9811986.05
PG1017	PGN1338	*ppdK pyruvate phosphate dikinase*	0.000000	727.14	1116.5	-0.62	10.85	G	G	0.005415	-0.46	615602388	846158749
PG1014	PGN1340	*TPR domain protein*	0.039368	2.13	14.5	-2.77	4.06	Y	G	0.003852	-6.26	66413	5157638.3
PG1013	PGN1341	*acetyl-CoA hydrolase/transferase family protein*	0.001687	482.98	563	-0.22	10.03	G	R	0.007440	0.06	482577974	461839748
PG1012	PGN1342	*tRNA-i(6)A37 modification enzyme MiaB*	0.173614	2.13	12	-2.49	3.82	Y	Y	0.015386	-6.65	66413	7514169.13
PG1010	PGN1343	*ABC transporter ATP-binding protein*	0.309110	30.92	44.5	-0.53	6.24	Y	G	0.000327	-0.66	20784669	32777918.9
PG1009	PGN1344	*hypothetical protein PG_1009*	0.206110	8.53	7	0.29	3.96	Y	G	0.005266	-3.51	703430	3165665.18
PG1008	PGN1345	*hypothetical protein PG_1008*	0.400806	2.13	5	-1.23	2.83	Y	Y	0.061725	-0.74	66413	243923.5
PG1007	PGN1346	*transcriptional regulator GntR family*	0.543202	2.13	9	-2.08	3.48	Y	G	0.000670	-6.01	66413	4244878.18
PG1006	PGN1347	*hypothetical protein PG_1006*	0.766795	86.36	94.5	-0.13	7.5	Y	Y	0.037727	0.46	104036836	73490598.7
PG1004	PGN1349	*prolyl oligopeptidase family protein*	0.006908	542.69	486.5	0.16	10.01	R	Y	0.045407	0.15	696743544	625278135
PG1003	PGN1350	*conserved hypothetical protein*	0.784897	2.13	5.5	-1.37	2.93	Y	G	0.001008	-4.27	66413	1274131.6
PG1215	PGN1354	*lipoprotein protein putative*	0.600536	19.19	20.5	-0.1	5.31	Y	Y	0.065448	-0.33	10713810.5	13229042.5
PG1217	PGN1356	*hypothetical protein PG_1217*	0.000017	20.26	53.5	-1.4	6.2	G	G	0.001266	-1.42	6366583	16717992.7
PG1219	PGN1358	*hypothetical protein PG_1219*	0.629971	66.1	53.5	0.31	6.9	Y	R	0.007304	0.79	90122798.5	52681804.5
PG1220	PGN1359	*erythronate-4-phosphate dehydrogenase putative*	0.018560	2.13	15.5	-2.86	4.14	Y	G	0.003602	-6.78	66413	7408642.03
PG1221	PGN1360	*oxidoreductase short chain dehydrogenase/reductase family*	0.604563	2.13	4	-0.91	2.62	Y	Y	0.052147	-1.68	66413	863011.5
PG1225	PGN1363	*ABC transporter ATP-binding protein*	0.000371	2.13	20.5	-3.27	4.5	Y	G	0.004189	-4.92	66413	2047821.38
PG1226	PGN1364	*peptidyl-prolyl cis-trans isomerase cyclophilin-type*	0.035356	2.13	14.5	-2.77	4.06	Y	G	0.003580	-7.39	66413	11242766.3

TIGR / PGN	PgSgFn / Pg Spectral Count q-value	PgSgFn	Pg	log_2Ratio	log_2Sum	Regulation (Spectral Count)	Regulation (Protein Intensity)	PgSgFn / Pg Protein Intensity q-value	log_2Ratio	PgSgFn	Pg
PG1230	0.000051	139.67	206	-0.56	8.43	G	G	0.003764	-0.68	64189206	102278946
PGN1366	*hypothetical protein PG_1230*										
PG1232	0.000000	10073.35	7559.5	0.41	14.11	R	R	0.008613	0.11	1.0952e+10	1.0158e+10
PGN1367	*gdh glutamate dehydrogenase NAD-specific*										
PG1235	0.061342	233.5	285	-0.29	9.02	Y	Y	0.024215	-0.19	209992420	240104441
PGN1370	*epimerase/reductase putative*										
PG1236	0.635799	69.3	57.5	0.27	6.99	Y	Y	0.020429	0.36	27222741	21276529.2
PGN1372	*hypothetical protein PG_1236*										
PG1237	0.007047	2.13	17	-3	4.26	Y	G	0.001392	-8.29	66413	20802399.9
PGN1373	*transcriptional regulator LuxR family*										
PG1239	0.000000	180.19	326	-0.86	8.98	G	Y	0.123473	0.13	174127855	154282117
PGN1375	*fabG 3-oxoacyl-(acyl-carrier-protein) reductase*										
PG1240	0.273961	21.32	34	-0.67	5.79	Y	Y	0.024343	-0.31	11867370.5	14748672.1
PGN1376	*transcriptional regulator tetR family*										
PG1241	0.000000	9.6	41.5	-2.11	5.68	G	Y	0.011057	-3.82	2266589.5	8270883.58
PGN1377	*lepA GTP-binding protein Lepa*										
PG1242	0.134175	2.13	12.5	-2.55	3.87	Y	Y	0.030329	-7.02	66413	12114954.7
PGN1378	*dnaB replicative DNA helicase*										
PG1246	0.002530	296.4	229.5	0.37	9.04	R	Y	0.124354	0.07	163776703	152958394
PGN1381	*alaS alanyl-tRNA synthetase*										
PG1247	0.758093	2.13	6.5	-1.61	3.11	Y	G	0.003885	-5.43	66413	2897898.5
PGN1382	*aroB 3-dehydroquinate synthase*										
PG1248	0.102520	8.53	17	-0.99	4.67	Y	G	0.003925	-5.09	253895.5	5575176.88
PGN1383	*conserved hypothetical protein*										
PG1249	0.608229	2.13	8.5	-2	3.41	Y	G	0.002417	-6.23	66413	4988978.8
PGN1384	*1-acyl-sn-glycerol-3-phosphate acetyltransferase putative*										
PG1253	0.001591	2.13	19	-3.16	4.4	Y	G	0.000446	-7.13	66413	9254131.63
PGN1388	*ligA DNA ligase NAD-dependent*										
PG1256	0.000152	19.19	47.5	-1.31	6.06	G	G	0.000476	-1.51	10560192.5	29952570
PGN1391	*ribonuclease Rne/Rng family*										
PG1258	0.275068	56.51	43.5	0.38	6.64	Y	Y	0.109736	0.26	72082116.5	61572450.5
PGN1393	*hup-2 DNA-binding protein HU*										
PG1259	0.784897	2.13	5.5	-1.37	2.93	Y	G	0.001293	-3.35	66413	674308.5
PGN1395	*nrdG anaerobic ribonucleoside-triphosphate reductase activating protein*										
PG1260	0.161227	306	317.5	-0.05	9.28	Y	G	0.006461	-0.46	197363410	271899860
PGN1396	*anaerobic ribonucleoside-triphosphate reductase putative*										
PG1269	0.000045	346.51	256.5	0.43	9.24	R	R	0.008738	0.19	283711901	249493767
PGN1401	*pruA delta-1-pyrroline-5-carboxylate dehydrogenase*										
PG1270	0.001065	2.13	19.5	-3.19	4.43	Y	G	0.000295	-7.97	66413	16539277.9
PGN1402	*conserved hypothetical protein*										
PG1271	0.000042	154.6	223	-0.53	8.56	G	G	0.000630	-0.60	141169939	214105302
PGN1403	*acetylornithine aminotransferase putative*										
PG0568	0.007665	25.59	48.5	-0.92	6.21	G	R	0.000998	2.01	41770900.5	10356608
PGN1405	*efp-1 translation elongation factor P*										
PG0562	0.784897	2.13	5.5	-1.37	2.93	Y	G	0.002392	-4.78	66413	1831556.08
PGN1408	*potassium uptake protein TrkA putative*										
PG0561	0.579385	347.58	357	-0.04	9.46	Y	G	0.007920	-0.50	226721953	321016073
PGN1409	*peptidase M20/M25/M40 family*										

TIGR	PGN	Description	PgSgFn / Pg Spectral Count *q*-value	PgSgFn	Pg	log_2Ratio	log_2Sum	Regulation (Spectral Count)	Regulation (Protein Intensity)	PgSgFn / Pg Protein Intensity *q*-value	log_2Ratio	PgSgFn	Pg
PG0559	PGN1411	chlorohydrolase family protein	0.010680	8.53	22	-1.37	4.93	Y	G	0.005673	-4.91	2132827	14965411.7
PG0558	PGN1412	purine nucleoside phosphorylase I inosine and guanosine-specific	0.000000	173.79	440	-1.34	9.26	G	G	0.002693	-0.54	135437828	196070652
PG0553	PGN1416	extracellular protease putative	0.456632	285.74	287	-0.01	9.16	Y	Y	0.052192	-0.20	204340669	232411885
PG0548	PGN1418	pyruvate ferredoxin/flavodoxin oxidoreductase family protein	0.000000	990.49	762.5	0.38	10.78	R	Y	0.077211	0.11	644782193	591512020
PG0547	PGN1419	conserved hypothetical protein	0.256970	115.15	91	0.34	7.69	Y	R	0.006226	0.25	74920007.5	63028366.3
PG0541	PGN1429	hypothetical protein PG_0541	0.779998	2.13	6	-1.49	3.02	Y	G	0.000323	-6.13	66413	4603960.55
PG0539	PGN1431	efflux transporter MFP component RND family	0.000123	2.13	22	-3.37	4.59	Y	G	0.000674	-7.24	66413	9956127.78
PG0538	PGN1432	outer membrane efflux protein	0.012108	8.53	22.5	-1.4	4.96	Y	Y	0.029520	-3.76	3583101.5	8646775.08
PG0537	PGN1434	pepD-2 aminoacyl-histidine dipeptidase	0.000022	1225.05	1045	0.23	11.15	R	Y	0.188417	0.00	969255145	967602451
PG0535	PGN1436	conserved hypothetical protein	0.457936	50.11	38.5	0.38	6.47	Y	R	0.000941	0.72	42310828	25716380
PG0534	PGN1437	hypothetical protein PG_0534	0.000000	17.06	57	-1.74	6.21	G	Y	0.024472	-4.46	11046705	28439829.4
PG0532	PGN1440	conserved domain protein	0.600049	2.13	4	-0.91	2.62	Y	Y	0.053939	-1.15	66413	540795.5
PG0531	PGN1441	nadE glutamine-dependent NAD+ synthetase	0.259167	85.3	107.5	-0.33	7.59	Y	Y	0.186035	0.00	80810482.5	80443230.2
PG0530	PGN1443	carB carbamoyl-phosphate synthase large subunit	0.492919	154.6	139	0.15	8.2	Y	Y	0.018197	-0.35	94544242.5	120693110
PG0529	PGN1444	carA carbamoyl-phosphate synthase small subunit	0.004661	25.59	48.5	-0.92	6.21	G	G	0.002890	-1.34	9929761	25259380.9
PG0528	PGN1445	amidophosphoribosyltransferase putative	0.779998	67.17	67.5	-0.01	7.07	Y	Y	0.022094	-0.74	21418079	36347930.4
PG0526	PGN1446	membrane protein putative	0.008604	8.53	24	-1.49	5.02	G	Y	0.135130	-3.35	9430454	12334337.6
PG0525	PGN1447	pyrG CTP synthase	0.706763	49.04	57.5	-0.23	6.74	Y	G	0.006371	-0.81	28996692.5	50451920.8
PG0523	PGN1449	guaB inosine-5'-monophosphate dehydrogenase	0.751373	169.52	184.5	-0.12	8.47	Y	Y	0.025368	-0.55	76933219.5	109250594
PG0521	PGN1451	groES chaperonin 10 kDa	0.153288	73.57	71.5	0.04	7.18	Y	Y	0.166018	-0.31	88773434	79619171.6
PG0520	PGN1452	groEL chaperonin 60 kDa	0.000979	1587.55	1720	-0.12	11.69	G	Y	0.122361	-0.10	734779497	778944771
PG0517	PGN1455	hypothetical protein PG_0517	0.309161	2.13	10	-2.23	3.6	Y	G	0.000295	-5.59	66413	3158489.5
PG0516	PGN1456	conserved hypothetical protein	0.182832	2.13	11.5	-2.43	3.77	Y	G	0.008194	-5.06	66413	2328561.83
PG0515	PGN1457	conserved hypothetical protein	0.219720	18.13	30	-0.73	5.59	Y	Y	0.019812	-0.67	9362830.5	14694655.8

TIGR	PgSgFn / Pg Spectral Count							PgSgFn / Pg Protein Intensity			
PGN	q-value	PgSgFn	Pg	log_2Ratio	log_2Sum	Regulation		q-value	log_2Ratio	PgSgFn	Pg
PG0514	0.000018	248.42	330	-0.41	9.18	G	G	0.007211	-0.47	139260079	191792057
PGN1458	secA preprotein translocase SecA subunit										
PG0513	0.000201	2.13	21.5	-3.34	4.56	Y	G	0.001046	-7.05	66413	8761978.88
PGN1459	conserved hypothetical protein TIGR00255										
PG0512	0.284896	8.53	10	-0.23	4.21	Y	Y	0.128525	-2.31	6285985	4192217.73
PGN1460	gmk guanylate kinase										
PG0506	0.001131	3879.86	3595.5	0.11	12.87	R	R	0.005274	0.30	2.6713e+09	2.1608e+09
PGN1466	prtRII arginine-specific cysteine proteinase										
PG0504	0.779998	2.13	2.5	-0.23	2.21	Y	Y	0.055401	-1.10	66413	390168.025
PGN1468	lipA lipoate synthase										
PG0503	0.156782	99.16	129	-0.38	7.83	Y	G	0.001022	-0.99	45611464	90319581.9
PGN1469	dpp dipeptidyl aminopeptidase IV										
PG0500	0.786190	34.12	35	-0.04	6.11	Y	Y	0.013203	0.55	12444619.5	8638450.55
PGN1472	tgt queuine tRNA-ribosyltransferase										
PG0498	0.667335	2.13	8	-1.91	3.34	Y	G	0.004014	-5.59	66413	3243033.6
PGN1474	luxS autoinducer-2 production protein LuxS										
PG0495	0.001057	299.6	239	0.33	9.07	R	Y	0.039451	0.26	285894626	238536122
PGN1476	hypothetical protein PG_0495										
PG0491	0.000001	995.82	883.5	0.17	10.88	R	Y	0.061725	-0.06	999229579	1.0426e+09
PGN1479	conserved hypothetical protein										
PG0490	0.102044	6.4	17	-1.41	4.55	Y	Y	0.042801	-4.08	7151271.5	15254061.4
PGN1480	membrane protein putative										
PG0488	0.045023	10.66	21	-0.98	4.98	Y	Y	0.016816	-3.85	3246191.5	10230794.5
PGN1482	ruvB Holliday junction DNA helicase RuvB										
PG0486	0.659176	2.13	8	-1.91	3.34	Y	G	0.006558	-5.51	66413	3130300.95
PGN1484	ogt methylated-DNA--protein-cysteine S-methyltransferase										
PG0485	0.604563	85.3	101	-0.24	7.54	Y	Y	0.028829	-0.25	38683622.5	45922536.5
PGN1485	yajC preprotein translocase YajC subunit										
PG0484	0.274290	2.13	10.5	-2.3	3.66	Y	Y	0.019992	-6.22	66413	5892658.3
PGN1486	hypothetical protein PG_0484										
PG0482	0.784897	38.38	38.5	0	6.26	Y	Y	0.033883	-0.55	37989557.5	53054156.4
PGN1488	hypothetical protein PG_0482										
PG0481	0.000000	384.89	581	-0.59	9.92	G	Y	0.049354	0.09	466538344	437303734
PGN1489	kbl 2-amino-3-ketobutyrate CoA ligase										
PG0479	0.773021	2.13	6.5	-1.61	3.11	Y	G	0.001865	-4.59	66413	1595571.58
PGN1491	hypothetical protein PG_0479										
PG0477	0.344563	108.75	100	0.12	7.71	Y	Y	0.111797	-0.18	43383274.5	48543513.2
PGN1492	panC pantoate--beta-alanine ligase										
PG0476	0.056342	20.26	35	-0.79	5.79	Y	Y	0.171340	-0.04	15971980.5	15363446.1
PGN1493	yngK-2 yngK protein										
PG0474	0.779998	2.13	6	-1.49	3.02	Y	Y	0.034691	-5.74	66413	5437337.5
PGN1495	low-specificity L-threonine aldolase										
PG0471	0.751373	2.13	3	-0.49	2.36	Y	Y	0.052108	-1.66	66413	1091154.5
PGN1498	hypothetical protein PG_0471										
PG0468	0.126368	17.06	30.5	-0.84	5.57	Y	Y	0.010571	-0.82	9336391.5	16588815.9
PGN1501	manA mannose-6-phosphate isomerase class I										
PG0466	0.000979	2.13	19.5	-3.19	4.43	Y	G	0.004528	-7.80	66413	15080789.3
PGN1502	hypothetical protein PG_0466										

TIGR	PgSgFn / Pg Spectral Count						PgSgFn / Pg Protein Intensity				
PGN	*q*-value	PgSgFn	Pg	log_2Ratio	log_2Sum	Regulation		*q*-value	log_2Ratio	PgSgFn	Pg
PG0465	0.201724	22.39	34.5	-0.62	5.83	Y	G	0.004701	-0.81	4930645.5	8687355.9
PGN1503	*fur ferric uptake transcriptional regulator*										
PG0464	0.008977	234.56	176	0.41	8.68	R	Y	0.127626	-0.05	145076936	151399784
PGN1504	*purA adenylosuccinate synthetase*										
PG0463	0.458416	26.65	32	-0.26	5.87	Y	Y	0.105603	-0.47	5294566.5	6660247.58
PGN1505	*folC folylpolyglutamate synthase*										
PG0462	0.000000	21.32	73.5	-1.79	6.57	G	G	0.000107	-2.38	9187182	47663749.5
PGN1506	*transporter putative*										
PG0452	0.062104	491.51	473	0.06	9.91	Y	Y	0.029655	0.18	568926495	500831456
PGN1510	*conserved hypothetical protein*										
PG0451	0.751373	2.13	3	-0.49	2.36	Y	Y	0.052108	-1.77	66413	977163.6
PGN1511	*CBS domain protein*										
PG0449	0.000000	268.68	138	0.96	8.67	R	R	0.005100	0.77	275338374	162894846
PGN1513	*TPR domain protein*										
PG0448	0.002517	99.16	135.5	-0.45	7.87	G	Y	0.042501	-0.26	64967812	77042535.8
PGN1514	*hypothetical protein PG_0448*										
PG0447	0.243573	6.4	15.5	-1.28	4.45	Y	G	0.004675	-4.05	1737542	8419077.3
PGN1515	*conserved hypothetical protein*										
PG0445	0.001704	122.61	175.5	-0.52	8.22	G	Y	0.010555	-0.38	91592883.5	119574802
PGN1517	*pepT peptidase T*										
PG0444	0.047330	9.6	19	-0.98	4.84	Y	Y	0.053629	-4.12	8353827	18151707.1
PGN1518	*oligopeptide transporter OPT family*										
PG0443	0.182332	99.16	120	-0.28	7.78	Y	G	0.006115	-0.30	101836336	125140645
PGN1519	*hemagglutinin-related protein*										
PG0441	0.398768	2.13	10	-2.23	3.6	Y	G	0.004388	-5.28	66413	2628415.53
PGN1520	*hypothetical protein PG_0441*										
PG0437	0.774795	27.72	31.5	-0.18	5.89	Y	G	0.002890	-0.48	19139437.5	26681187
PGN1523	*polysaccharide export protein BexD/CtrA/VexA family*										
PG0436	0.639633	85.3	93	-0.12	7.48	Y	Y	0.020525	-0.49	21087446.5	28925421.7
PGN1524	*capsular polysaccharide transport protein putative*										
PG0435	0.600049	2.13	8.5	-2	3.41	Y	G	0.000159	-6.06	66413	4386078.3
PGN1525	*capsular polysaccharide biosythesis protein putative*										
PG0434	0.784897	2.13	5.5	-1.37	2.93	Y	Y	0.017487	-5.05	66413	2521465.3
PGN1526	*hypothetical protein PG_0434*										
PG0433	0.006232	7.46	23	-1.62	4.93	G	Y	0.013917	-3.79	1833778	6320235.6
PGN1527	*tetrapyrrole methylase family protein*										
PG0432	0.000408	7.46	27	-1.86	5.11	G	Y	0.173433	-3.15	12250860	10976273.5
PGN1528	*NOL1/NOP2/sun family protein*										
PG0430	0.000000	619.46	460.5	0.43	10.08	R	Y	0.052108	-0.10	438824770	472866611
PGN1529	*oxidoreductase putative*										
PG0429	0.000000	1259.17	1057.5	0.25	11.18	R	R	0.004388	0.38	841964862	646793198
PGN1530	*pyruvate synthase*										
PG0540	0.516140	19.19	28	-0.55	5.56	Y	G	0.000584	-2.07	3683802	15439750.5
PGN1540	*AcrB/AcrD/AcrF family protein*										
PG0423	0.272530	8.53	14.5	-0.77	4.53	Y	Y	0.044266	-4.05	7972425.5	19198037.9
PGN1544	*hypothetical protein PG_0423*										
PG0421	0.014598	8.53	22	-1.37	4.93	Y	Y	0.023219	-4.08	4703888.5	12332220.2
PGN1547	*hypothetical protein PG_0421*										

TIGR	PgSgFn / Pg Spectral Count						PgSgFn / Pg Protein Intensity				
PGN	*q*-value	PgSgFn	Pg	log_2Ratio	log_2Sum	Regulation		*q*-value	log_2Ratio	PgSgFn	Pg
PG0418	0.334790	33.05	47	-0.51	6.32	Y	G	0.000446	-0.80	25633971	44463742.5
PGN1549	*clpP ATP-dependent Clp protease proteolytic subunit*										
PG0417	0.024177	18.13	36.5	-1.01	5.77	Y	Y	0.027084	-0.65	15452316	25093692.7
PGN1550	*clpX ATP-dependent Clp protease ATP-binding subunit ClpX*										
PG0416	0.441634	58.64	53.5	0.13	6.81	Y	Y	0.026780	-0.43	13393794	17772678.5
PGN1551	*recQ-1 ATP-dependent DNA helicase RecQ*										
PG0415	0.430790	73.57	80	-0.12	7.26	Y	G	0.000206	-0.25	39511242.5	47005262.9
PGN1552	*peptidyl-prolyl cis-trans isomerase PPIC-type*										
PG0414	0.021655	2.13	15	-2.82	4.1	Y	Y	0.014936	-5.22	66413	2775469.95
PGN1553	*hypothetical protein PG_0414*										
PG0413	0.035356	9.6	2.5	1.94	3.6	Y	Y	0.172311	0.84	1217443.5	1406502
PGN1554	*hypothetical protein PG_0413*										
PG0412	0.042457	8.53	19.5	-1.19	4.81	Y	Y	0.020429	-4.02	3944306.5	10810044.9
PGN1555	*mutL DNA mismatch repair protein MutL*										
PG0411	0.492890	77.83	90.5	-0.22	7.4	Y	Y	0.188417	-0.13	22293332.5	22004748.3
PGN1556	*hemagglutinin putative*										
PG0409	0.170868	58.64	62.5	-0.09	6.92	Y	Y	0.051674	0.19	32076362	28300101
PGN1557	*hypothetical protein PG_0409*										
PG0401	0.346258	117.28	136.5	-0.22	7.99	Y	Y	0.050907	-0.20	93489082	107388420
PGN1564	*KH/HDIG domain protein*										
PG0400	0.410625	10.66	11	-0.05	4.44	Y	Y	0.084073	-2.69	2440644.5	3941891.13
PGN1565	*conserved hypothetical protein*										
PG0398	0.000262	2.13	21	-3.3	4.53	Y	Y	0.014886	-5.92	66413	4522939.08
PGN1567	*recF recF protein*										
PG0396	0.492890	9.6	8.5	0.18	4.18	Y	Y	0.041109	-2.26	755195.5	1608718.65
PGN1569	*transcriptional regulator Crp/Fnr family*										
PG0395	0.447261	601.33	573	0.07	10.2	Y	Y	0.119527	-0.04	415731064	426181260
PGN1570	*rpoC DNA-directed RNA polymerase beta' subunit*										
PG0394	0.061251	504.31	562.5	-0.16	10.06	Y	Y	0.088585	-0.05	377120841	390109093
PGN1571	*rpoB DNA-directed RNA polymerase beta subunit*										
PG0393	0.018215	842.29	939	-0.16	10.8	Y	R	0.003742	0.43	1.8494e+09	1.3761e+09
PGN1572	*rplL ribosomal protein L7/L12*										
PG0392	0.000000	1113.1	642.5	0.79	10.78	R	Y	0.033358	0.41	464861931	344565272
PGN1573	*rplJ ribosomal protein L10*										
PG0391	0.000000	502.17	162	1.63	9.38	R	Y	0.011751	0.72	186561733	111808114
PGN1574	*rplA ribosomal protein L1*										
PG0390	0.000000	206.84	105.5	0.97	8.29	R	Y	0.043953	0.25	577374420	478623837
PGN1575	*rplK ribosomal protein L11*										
PG0389	0.397614	94.89	115	-0.28	7.71	Y	G	0.006764	-0.91	22916872	43407048.5
PGN1576	*nusG transcription antitermination protein NusG*										
PG0387	0.000000	2992.79	1743	0.78	12.21	R	Y	0.026972	0.14	3.6929e+09	3.3677e+09
PGN1578	*tuf translation elongation factor Tu*										
PG0386	0.283422	334.78	296.5	0.18	9.3	Y	Y	0.022948	0.10	185030617	173190186
PGN1579	*site-specific recombinase phage integrase family/ribosomal subunit interface protein*										
PG0384	0.328062	9.6	10.5	-0.13	4.33	Y	Y	0.052147	-3.03	2674934	5180859.9
PGN1581	*MutS2 family protein*										
PG0383	0.383939	2.13	10	-2.23	3.6	Y	G	0.000492	-5.99	66413	4199100.05
PGN1582	*membrane-associated zinc metalloprotease putative*										

TIGR	PgSgFn / Pg Spectral Count							PgSgFn / Pg Protein Intensity			
PGN	q-value	PgSgFn	Pg	log_2Ratio	log_2Sum	Regulation		q-value	log_2Ratio	PgSgFn	Pg
PG0380	0.000141	2.13	22	-3.37	4.59	Y	G	0.008458	-6.95	66413	8685424.15
PGN1585	uvrB excinuclease ABC B subunit										
PG0378	0.000000	635.45	361	0.82	9.96	R	R	0.009124	0.28	438435471	361794829
PGN1587	tsf translation elongation factor Ts										
PG0377	0.457936	307.06	302	0.02	9.25	Y	Y	0.165042	-0.04	242595388	247756522
PGN1588	rpsB ribosomal protein S2										
PG0376	0.000000	251.62	142.5	0.82	8.62	R	R	0.000561	0.91	91043955.5	48500330
PGN1589	rpsI ribosomal protein S9										
PG0375	0.010705	170.59	123.5	0.47	8.2	Y	R	0.006461	0.41	121584978	91914945.3
PGN1590	rplM ribosomal protein L13										
PG0369	0.732133	25.59	20.5	0.32	5.53	Y	G	0.007305	-0.87	3867696	7101794.2
PGN1593	coaD phosphopantetheine adenylyltransferase										
PG0368	0.000015	2.13	24.5	-3.52	4.73	Y	G	0.006049	-7.18	66413	9967298.75
PGN1594	DNA topoisomerase IV B subunit putative										
PG0366	0.751373	2.13	7	-1.72	3.19	Y	G	0.001445	-5.28	66413	2571241.9
PGN1595	hypothetical protein PG_0366										
PG0365	0.021047	2.13	15.5	-2.86	4.14	Y	G	0.000585	-5.04	66413	2167827.63
PGN1596	3'-5' exonuclease domain protein										
PG0364	0.039231	2.13	14.5	-2.77	4.06	Y	G	0.000584	-6.13	66413	4624607.25
PGN1597	conserved hypothetical protein										
PG0362	0.031975	9.6	19.5	-1.02	4.86	Y	Y	0.114945	-3.43	6624937.5	9362823.43
PGN1599	hypothetical protein PG_0362										
PG0361	0.115168	8.53	18.5	-1.12	4.76	Y	G	0.006461	-3.88	1625634	7276935.98
PGN1600	conserved domain protein										
PG0360	0.652130	54.38	48.5	0.17	6.68	Y	Y	0.088585	0.11	37781630	34932838.7
PGN1601	lemA protein										
PG0359	0.779998	2.13	6	-1.49	3.02	Y	G	0.000462	-5.77	66413	3601897.23
PGN1602	flavin reductase domain protein										
PG0358	0.024177	2.13	15	-2.82	4.1	Y	G	0.005288	-7.02	66413	8844550.78
PGN1603	pyrI aspartate carbamoyltransferase regulatory subunit										
PG0357	0.714916	49.04	43.5	0.17	6.53	Y	Y	0.058540	0.18	42319736.5	37211296
PGN1604	pyrB aspartate carbamoyltransferase catalytic subunit										
PG0356	0.000000	12.79	49	-1.94	5.95	G	Y	0.016418	-4.59	5326173	22748181.9
PGN1605	conserved hypothetical protein										
PG0350	0.770282	181.25	190.5	-0.07	8.54	Y	Y	0.049785	0.32	224751036	183721873
PGN1611	internalin-related protein										
PG0348	0.685422	2.13	3.5	-0.72	2.49	Y	Y	0.052108	-2.33	66413	2721258.5
PGN1613	recG ATP-dependent DNA helicase RecG										
PG0347	0.659176	41.58	51	-0.29	6.53	Y	Y	0.031695	-0.33	23222175	28658677.3
PGN1614	galE UDP-glucose 4-epimerase										
PG0346	0.199425	8.53	13.5	-0.66	4.46	Y	Y	0.089345	-3.19	3867773	6336013.15
PGN1615	GTP-binding protein										
PG0344	0.148470	12.79	17	-0.41	4.9	Y	Y	0.027355	-3.70	4239952.5	10424632.6
PGN1617	purple acid phosphatase										
PG0343	0.000000	1221.85	1373	-0.17	11.34	G	G	0.005129	-0.48	462480587	645066828
PGN1618	megL methionine gamma-lyase										
PG0339	0.486452	9.6	12	-0.32	4.43	Y	Y	0.028829	-2.16	432901	1147921.35
PGN1622	hypothetical protein PG_0339										

TIGR	PgSgFn / Pg Spectral Count						PgSgFn / Pg Protein Intensity				
PGN	*q*-value	PgSgFn	Pg	log_2Ratio	log_2Sum	Regulation		*q*-value	log_2Ratio	PgSgFn	Pg
PG0338	0.000000	2.13	45.5	-4.42	5.57	Y	G	0.000005	-7.39	66413	11045658.2
PGN1623	*hypothetical protein PG_0338*										
PG0336	0.000000	23.46	46	-0.97	6.12	G	Y	0.096757	-3.35	6855146	10427369.1
PGN1625	*hypothetical protein PG_0336*										
PG0334	0.389823	39.45	54.5	-0.47	6.55	Y	G	0.004947	-0.51	9482461.5	13517124.3
PGN1628	*glycosyl transferase group 2 family protein*										
PG0332	0.631759	109.82	114.5	-0.06	7.81	Y	Y	0.027198	-0.36	69931104.5	90058764.5
PGN1630	*rho transcription termination factor Rho*										
PG0330	0.000000	130.08	59.5	1.13	7.57	R	Y	0.103175	-0.04	137526882	141151058
PGN1631	*DNA-binding protein histone-like family*										
PG0329	0.000000	381.7	547.5	-0.52	9.86	G	Y	0.011984	-0.46	413149770	568563164
PGN1633	*formiminotransferase-cyclodeaminase-related protein*										
PG0328	0.046146	63.97	86	-0.43	7.23	Y	Y	0.017665	-0.45	49637981.5	66604803.4
PGN1634	*hutI imidazolonepropionase*										
PG0327	0.383939	2.13	10	-2.23	3.6	Y	G	0.008538	-6.37	66413	5814700.5
PGN1635	*hypothetical protein PG_0327*										
PG0326	0.000000	20.26	59.5	-1.55	6.32	G	Y	0.016418	-4.51	6818313	23690279.7
PGN1636	*hypothetical protein PG_0326*										
PG0325	0.000000	384.89	253	0.61	9.32	R	G	0.002035	-0.17	165112101	186239847
PGN1637	*conserved hypothetical protein*										
PG0324	0.523440	190.85	190.5	0	8.57	Y	G	0.009482	-0.24	145106967	171863314
PGN1638	*hutH histidine ammonia-lyase*										
PG0323	0.000000	100.22	46	1.12	7.19	R	Y	0.172311	-0.01	29140455.5	29589309.5
PGN1639	*conserved hypothetical protein*										
PG0321	0.779998	27.72	27.5	0.01	5.79	Y	Y	0.022094	0.84	29738956	16388090
PGN1641	*LAO/AO transport system ATPase*										
PG0319	0.000018	77.83	39	1	6.87	R	R	0.009001	1.17	83923687.5	36726884.3
PGN1643	*hypothetical protein PG_0319*										
PG0317	0.000387	14.93	29.5	-0.98	5.47	G	Y	0.025864	-4.24	5267472.5	14959937.3
PGN1645	*peptidase M49 family*										
PG0316	0.102044	92.76	104.5	-0.17	7.62	Y	Y	0.058124	0.11	40752777	37826893
PGN1646	*serS seryl-tRNA synthetase*										
PG0315	0.000001	17.06	3	2.51	4.33	Y	Y	0.079379	1.58	8366899.5	2668978.5
PGN1647	*rpmA ribosomal protein L27*										
PG0314	0.000182	47.98	24.5	0.97	6.18	R	R	0.003764	0.51	39782166.5	27968493.8
PGN1648	*rplU ribosomal protein L21*										
PG0311	0.240620	8.53	13	-0.61	4.43	Y	Y	0.052192	-3.03	2003483.5	3875958.78
PGN1651	*glycosyl transferase group 2 family protein*										
PG0310	0.348525	43.71	48	-0.14	6.52	Y	Y	0.163917	0.06	27216100.5	26419807
PGN1652	*nitroreductase family protein*										
PG0306	0.022165	90.63	122.5	-0.43	7.74	Y	G	0.000636	-0.67	74876314.5	119355832
PGN1656	*electron transport complex RnfABCDGE type G subunit*										
PG0305	0.758460	15.99	12	0.41	4.81	Y	Y	0.120505	-0.13	8330996	8951268.88
PGN1657	*electron transport complex RnfABCDGE type D subunit*										
PG0304	0.000381	489.38	478.5	0.03	9.92	R	Y	0.135130	0.06	401823549	385420079
PGN1658	*electron transport complex RnfABCDGE type C subunit*										
PG0303	0.002443	147.13	204	-0.47	8.46	G	Y	0.017665	-0.44	71730438	96616928.1
PGN1659	*electron transport complex RnfABCDGE type B subunit*										

TIGR / PGN	PgSgFn / Pg Spectral Count q-value	PgSgFn	Pg	log_2Ratio	log_2Sum	Regulation		PgSgFn / Pg Protein Intensity q-value	log_2Ratio	PgSgFn	Pg
PG0300	0.779998	2.13	2.5	-0.23	2.21	Y	Y	0.052192	-1.64	66413	817565.325
PGN1661	TPR domain protein										
PG0296	0.786190	309.19	298.5	0.05	9.25	Y	Y	0.142053	0.03	215021452	209885507
PGN1666	phosphoribosylformylglycinamidine synthase putative										
PG0295	0.751373	2.13	3	-0.49	2.36	Y	Y	0.053384	-1.35	66413	548506.65
PGN1667	dprA DNA processing protein DprA putative										
PG0294	0.751373	2.13	3	-0.49	2.36	Y	Y	0.052842	-1.46	66413	633436
PGN1668	glycosyl transferase group 2 family protein										
PG0293	0.000000	656.77	296.5	1.15	9.9	R	R	0.007580	0.48	858691770	614688055
PGN1670	secretion activator protein putative										
PG0291	0.097851	10.66	15	-0.49	4.68	Y	Y	0.093332	-3.59	7066864	11542441.2
PGN1673	hypothetical protein PG_0291										
PG0290	0.000506	19.19	45.5	-1.25	6.02	G	G	0.006115	-1.26	10874006	26096336.5
PGN1674	hypothetical protein PG_0290										
PG0289	0.751373	78.9	70.5	0.16	7.22	Y	Y	0.090791	0.13	42059691	37927948.2
PGN1675	hypothetical protein PG_0289										
PG0288	0.014675	25.59	46	-0.85	6.16	Y	Y	0.028659	-0.51	17049916	24402470.6
PGN1676	lipoprotein putative										
PG0287	0.784897	2.13	5.5	-1.37	2.93	Y	Y	0.014959	-4.91	66413	2224810.85
PGN1677	hypothetical protein PG_0287										
PG0286	0.010781	2.13	16.5	-2.95	4.22	Y	G	0.000492	-7.54	66413	12274092.7
PGN1678	hypothetical protein PG_0286										
PG0279	0.403113	122.61	129	-0.07	7.98	Y	Y	0.113888	-0.11	60383769	65807520.9
PGN1685	maeB NADP-dependent malic enzyme										
PG1747	0.030271	9.6	22	-1.2	4.98	Y	Y	0.024343	-3.96	5283792.5	14025138.4
PGN1688	ribose 5-phosphate isomerase B putative										
PG1748	0.243789	377.43	355	0.09	9.52	Y	Y	0.062627	-0.14	365058399	406090044
PGN1689	tkt transketolase										
PG1750	0.475857	2.13	9.5	-2.16	3.54	Y	G	0.006226	-5.11	66413	2372001.28
PGN1690	alpha-13/4-fucosidase putative										
PG1753	0.560362	2.13	8.5	-2	3.41	Y	G	0.004827	-5.62	66413	3328013.18
PGN1693	selD selenide water dikinase										
PG1754	0.720393	133.27	142	-0.09	8.1	Y	Y	0.038496	0.46	132725247	94459296.8
PGN1694	conserved domain protein										
PG1755	0.000000	722.88	525	0.46	10.29	R	R	0.007897	0.19	1.644e+09	1.4377e+09
PGN1695	fbaB fructose-bisphosphate aldolase class I										
PG1757	0.659176	51.18	50	0.03	6.66	Y	Y	0.090791	-0.14	29819663	32488824.8
PGN1697	hypothetical protein PG_1757										
PG1758	0.000000	125.81	65	0.95	7.58	R	R	0.002518	0.80	106327078	61352407.7
PGN1698	rpsO ribosomal protein S15										
PG1759	0.124784	14.93	15	-0.01	4.9	Y	Y	0.153159	-2.57	8819851.5	6964669.25
PGN1699	adhesion protein putative										
PG1762	0.000000	753.8	419	0.85	10.2	R	Y	0.011011	0.31	832969074	672647831
PGN1702	secDF protein-export membrane protein SecD/protein-export membrane protein SecF										
PG1763	0.779998	22.39	24	-0.1	5.54	Y	G	0.001675	-0.46	7506585	10324153.7
PGN1703	rnc ribonuclease III										
PG1764	0.000000	2281.64	2135	0.1	12.11	R	Y	0.073682	-0.10	997537790	1.0685e+09
PGN1704	fabF 3-oxoacyl-(acyl-carrier-protein) synthase II										

TIGR	PGN	PgSgFn / Pg Spectral Count q-value	PgSgFn	Pg	log_2Ratio	log_2Sum	Regulation (Spectral Count)	Regulation (Protein Intensity)	PgSgFn / Pg Protein Intensity q-value	log_2Ratio	PgSgFn	Pg
PG1765		0.000000	1227.18	449	1.45	10.71	R	R	0.007304	0.45	2.5781e+09	1.8925e+09
PGN1705	*acpP acyl carrier protein*											
PG1766		0.779998	2.13	6	-1.49	3.02	Y	G	0.006652	-5.52	66413	3168139.15
PGN1706	*purN phosphoribosylglycinamide formyltransferase*											
PG1767		0.543202	2.13	9	-2.08	3.48	Y	G	0.000770	-5.29	66413	2578297.15
PGN1707	*lipoprotein putative*											
PG1771		0.307024	75.7	89.5	-0.24	7.37	Y	G	0.001455	-0.57	23676048.5	35066909.3
PGN1711	*pheS phenylalanyl-tRNA synthetase alpha subunit*											
PG1772		0.720026	2.13	7.5	-1.82	3.27	Y	G	0.004884	-4.47	66413	1497949.18
PGN1712	*nth endonuclease III*											
PG1774		0.001729	14.93	29	-0.96	5.46	G	Y	0.116286	-3.07	5539359	7804806.2
PGN1714	*mfd transcription-repair coupling factor*											
PG1775		0.309348	62.91	47.5	0.41	6.79	Y	Y	0.054253	-0.35	23778711.5	30249325.5
PGN1715	*grpE grpE protein*											
PG1776		0.148377	21.32	35.5	-0.74	5.83	Y	Y	0.148734	0.11	5976622	5653923.25
PGN1716	*dnaJ dnaJ protein*											
PG1778		0.784897	2.13	5.5	-1.37	2.93	Y	G	0.004394	-4.89	66413	1998394.28
PGN1718	*conserved hypothetical protein*											
PG1779		0.115446	7.46	18	-1.27	4.67	Y	Y	0.021356	-4.00	5021358	14094156
PGN1719	*conserved hypothetical protein*											
PG1780		0.009141	50.11	75	-0.58	6.97	G	Y	0.056585	-0.70	34044385.5	49261362.3
PGN1721	*bioF-3 8-amino-7-oxononanoate synthase*											
PG1781		0.075029	29.85	49.5	-0.73	6.31	Y	G	0.001742	-1.04	10588055	21613114.2
PGN1722	*udk uridine kinase*											
PG1782		0.773021	2.13	6.5	-1.61	3.11	Y	G	0.000137	-4.41	66413	1396592.2
PGN1723	*hypothetical protein PG_1782*											
PG1783		0.327141	2.13	10.5	-2.3	3.66	Y	G	0.004124	-6.14	66413	4759324.83
PGN1724	*glycosyl transferase group 2 family protein*											
PG1841		0.600049	2.13	4	-0.91	2.62	Y	Y	0.052108	-1.75	66413	1228055
PGN1730	*conserved hypothetical protein*											
PG1837		0.000000	2785.95	2980	-0.1	12.49	G	R	0.009495	0.24	3.7254e+09	3.1681e+09
PGN1733	*hemagglutinin protein HagA*											
PG1835		0.730633	93.82	96	-0.03	7.57	Y	G	0.009010	-0.51	53666057	75689609
PGN1735	*lipoprotein putative*											
PG1834		0.751373	73.57	82	-0.16	7.28	Y	Y	0.105603	0.08	36875983	34708688.2
PGN1736	*glycogen synthase-related protein*											
PG1829		0.751373	101.29	106.5	-0.07	7.7	Y	G	0.002394	-0.34	50168145.5	63400497.5
PGN1738	*long-chain-fatty-acid--CoA ligase putative*											
PG1824		0.000000	1396.71	1782	-0.35	11.63	G	Y	0.161524	-0.01	812316134	819306250
PGN1743	*eno enolase*											
PG1823		0.278503	11.73	12.5	-0.09	4.6	Y	Y	0.067909	-3.62	7096590.5	14065854.1
PGN1744	*hypothetical protein PG_1823*											
PG1816		0.770282	147.13	144	0.03	8.19	Y	Y	0.191557	-0.01	86281992.5	85989385.2
PGN1749	*NAD(P)H dehydrogenase quinone family putative*											
PG1815		0.074495	2.13	13.5	-2.66	3.97	Y	G	0.001693	-6.34	66413	5394059.2
PGN1750	*kdsB 3-deoxy-D-manno-octulosonate cytidylyltransferase*											
PG1814		0.630045	2.13	8	-1.91	3.34	Y	G	0.001293	-5.19	66413	2414134.43
PGN1751	*dnaG DNA primase*											

TIGR	PGN	Description	PgSgFn / Pg Spectral Count						PgSgFn / Pg Protein Intensity				
			q-value	PgSgFn	Pg	log_2Ratio	log_2Sum	Regulation	Regulation	*q*-value	log_2Ratio	PgSgFn	Pg
PG1813	*PGN1752*	*ferredoxin 4Fe-4S*	0.240620	77.83	60	0.38	7.11	Y	R	0.008974	0.57	172748248	116256837
PG1812	*PGN1753*	*2-oxoglutarate oxidoreductase alpha subunit*	0.000000	2008.7	1668	0.27	11.84	R	Y	0.146675	-0.06	1.3809e+09	1.4301e+09
PG1810	*PGN1755*	*2-oxoglutarate oxidoreductase beta subunit*	0.000000	953.17	689.5	0.47	10.68	R	R	0.006414	0.28	529339848	436197483
PG1809	*PGN1756*	*2-oxoglutarate oxidoreductase gamma subunit*	0.014718	331.58	267.5	0.31	9.23	Y	Y	0.121255	-0.13	269860925	293145522
PG1808	*PGN1757*	*spoT guanosine-3'5'-bis(diphosphate) 3'-pyrophosphohydrolase*	0.004976	28.79	52.5	-0.87	6.35	G	Y	0.018791	-0.72	12836718	20359396.4
PG1806	*PGN1759*	*atpI v-type ATPase subunit I*	0.678703	59.71	60	-0.01	6.9	Y	Y	0.071676	-0.13	43735822.5	48316136.6
PG1805	*PGN1760*	*atpD v-type ATPase subunit D*	0.542387	46.91	35	0.42	6.36	Y	Y	0.022021	0.71	26366774	16032264.2
PG1804	*PGN1761*	*atpB v-type ATPase subunit B*	0.000000	425.41	554	-0.38	9.94	G	Y	0.045407	-0.14	234979920	257968578
PG1803	*PGN1762*	*atpA v-type ATPase subunit A*	0.358444	291.07	311	-0.1	9.23	Y	Y	0.028659	-0.26	214214809	257611159
PG1802	*PGN1763*	*hypothetical protein PG_1802*	0.495376	102.35	106	-0.05	7.7	Y	Y	0.171340	-0.03	49977891	50740209.2
PG1801	*PGN1764*	*v-type ATPase subunit E putative*	0.000924	311.33	275.5	0.18	9.2	R	Y	0.023375	-0.38	149135893	196047434
PG1798	*PGN1767*	*immunoreactive 46 kDa antigen PG99*	0.166597	180.19	211	-0.23	8.61	Y	G	0.005813	-0.89	61840661	115037879
PG1797	*PGN1768*	*DNA-binding response regulator/sensor histidine kinase*	0.000926	30.92	60.5	-0.97	6.51	G	G	0.006115	-0.83	14669193	25482607.7
PG1794	*PGN1771*	*polA DNA polymerase type I*	0.659176	19.19	26.5	-0.47	5.51	Y	G	0.000584	-0.69	8018163	12942828.8
PG1793	*PGN1772*	*glgB 14-alpha-glucan branching enzyme*	0.000000	20.26	59	-1.54	6.31	G	G	0.000803	-1.41	6841442.5	17907830.1
PG1792	*PGN1773*	*sodium/hydrogen antiporter*	0.751373	2.13	3	-0.49	2.36	Y	Y	0.052108	-1.90	66413	1164064
PG1789	*PGN1776*	*dcp-2 peptidyl-dipeptidase Dcp*	0.715624	67.17	63.5	0.08	7.03	Y	Y	0.016911	-0.72	16798585	26444031.3
PG1788	*PGN1777*	*cysteine peptidase putative*	0.275068	798.58	798	0	10.64	Y	Y	0.019992	0.03	870126881	851490387
PG1847	*PGN1780*	*endoribonuclease L-PSP putative*	0.000000	58.64	241.5	-2.04	8.23	G	G	0.002895	-1.73	24592510.5	78673188.5
PG1848	*PGN1781*	*RNA methyltransferase TrmH family*	0.163490	13.86	15	-0.11	4.85	Y	Y	0.059025	-3.38	4460240.5	9261481.38
PG1849	*PGN1782*	*recN DNA repair protein RecN*	0.007139	2.13	17	-3	4.26	Y	Y	0.024472	-6.76	66413	9153522.35
PG1850	*PGN1783*	*hypothetical protein PG_1850*	0.275068	2.13	11	-2.37	3.71	Y	G	0.001290	-5.77	66413	3615023.65
PG1851	*PGN1784*	*coaBC phosphopantothenoylcysteine decarboxylase/phosphopantothenate--cysteine ligase*	0.779998	44.78	43.5	0.04	6.46	Y	R	0.003861	0.48	23148490.5	16665640.8
PG1852	*PGN1785*	*exonuclease*	0.411177	70.37	76.5	-0.12	7.2	Y	Y	0.052108	0.35	29509355	23671097.2

TIGR	PGN	Description	PgSgFn / Pg Spectral Count: *q*-value	PgSgFn	Pg	log_2Ratio	log_2Sum	Regulation (Spectral Count)	Regulation (Protein Intensity)	PgSgFn / Pg Protein Intensity: *q*-value	log_2Ratio	PgSgFn	Pg
PG1853	*PGN1786*	*dnaN DNA polymerase III beta subunit*	0.000000	147.13	231.5	-0.65	8.56	G	G	0.001242	-0.80	64235326.5	111532876
PG1855	*PGN1788*	*carboxyl-terminal protease*	0.225691	20.26	33.5	-0.73	5.75	Y	Y	0.011777	-0.90	7634542	14203032.2
PG1856	*PGN1789*	*cytidine/deoxycytidylate deaminase family protein*	0.261642	2.13	11	-2.37	3.71	Y	G	0.005100	-5.94	66413	4185963
PG1858	*PGN1791*	*flavodoxin*	0.000000	4041.92	2878	0.49	12.76	R	Y	0.017985	0.24	1.8247e+09	1.5514e+09
PG1860	*PGN1793*	*conserved hypothetical protein*	0.786353	17.06	18	-0.08	5.13	Y	Y	0.171340	-0.03	2894601.5	2934973.6
PG1864	*PGN1796*	*leucine-rich protein*	0.146581	7.46	16	-1.1	4.55	Y	Y	0.022948	-3.41	1559096.5	4537047.78
PG1870	*PGN1798*	*methlytransferase UbiE/COQ5 family*	0.667335	2.13	8	-1.91	3.34	Y	G	0.000584	-5.76	66413	3579377
PG1872	*PGN1800*	*hutU urocanate hydratase*	0.000398	435	511.5	-0.23	9.89	G	G	0.001020	-0.49	288156424	404923931
PG1875	*PGN1802*	*hemolysin*	0.773021	2.13	6.5	-1.61	3.11	Y	G	0.000969	-4.60	66413	1596024.03
PG1876	*PGN1803*	*conserved hypothetical protein*	0.779998	2.13	6	-1.49	3.02	Y	G	0.001022	-5.50	66413	2988067.43
PG1877	*PGN1804*	*nhaA Na+/H+ antiporter*	0.604563	2.13	4	-0.91	2.62	Y	Y	0.052108	-2.33	66413	2108871.5
PG1878	*PGN1805*	*cysS cysteinyl-tRNA synthetase*	0.747683	94.89	98.5	-0.05	7.6	Y	Y	0.037877	0.20	46604670	40358889.5
PG1879	*PGN1806*	*conserved hypothetical protein*	0.120212	59.71	71.5	-0.26	7.04	Y	Y	0.014221	-0.67	27994037	45051657.8
PG1881	*PGN1808*	*hypothetical protein PG_1881*	0.667335	27.72	34	-0.29	5.95	Y	Y	0.179184	0.00	25265562.5	25533264.5
PG1884	*PGN1811*	*alpha-L-fucosidase precursor putative*	0.001586	24.52	52	-1.08	6.26	G	Y	0.011553	-1.01	12023521.5	24406954.9
PG1885	*PGN1812*	*ppk polyphosphate kinase*	0.197964	45.85	46.5	-0.02	6.53	Y	Y	0.055138	-0.57	18849786.5	27003585.2
PG1886	*PGN1813*	*hflX GTP-binding protein HflX*	0.786857	49.04	45.5	0.11	6.56	Y	R	0.003429	1.51	34212386.5	12070895.2
PG1887	*PGN1814*	*rhodanese-like domain protein*	0.457936	2.13	9.5	-2.16	3.54	Y	Y	0.028575	-4.80	66413	2477130.2
PG1888	*PGN1815*	*conserved hypothetical protein*	0.039231	2.13	14.5	-2.77	4.06	Y	G	0.000035	-6.05	66413	4362194.68
PG1889	*PGN1816*	*hypothetical protein PG_1889*	0.064048	2.13	13.5	-2.66	3.97	Y	Y	0.011359	-6.73	66413	7626854.9
PG1896	*PGN1827*	*metK S-adenosylmethionine synthase*	0.161227	125.81	100	0.33	7.82	Y	Y	0.014536	-0.58	48306032.5	72808450.3
PG1899	*PGN1830*	*TonB-dependent receptor putative*	0.295572	114.08	140.5	-0.3	7.99	Y	Y	0.137374	-0.15	94449457.5	102378567
PG1900	*PGN1831*	*conserved hypothetical protein TIGR00157*	0.345160	2.13	10	-2.23	3.6	Y	G	0.009482	-5.38	66413	2946555.1
PG1901	*PGN1832*	*frr ribosome recycling factor*	0.659176	82.1	70.5	0.22	7.25	Y	Y	0.157620	-0.03	137298116	142151773

TIGR	PGN	Description	PgSgFn / Pg Spectral Count q-value	PgSgFn	Pg	log_2Ratio	log_2Sum	Regulation (Spectral Count)	Regulation (Protein Intensity)	PgSgFn / Pg Protein Intensity q-value	log_2Ratio	PgSgFn	Pg
PG1902	PGN1833	*pyrH uridylate kinase*	0.000000	7.46	46	-2.62	5.74	G	Y	0.030979	-4.26	6914617	17167752.3
PG1903	PGN1834	*conserved hypothetical protein*	0.003541	19.19	42.5	-1.15	5.95	G	G	0.004902	-1.60	5316916	15608168.8
PG1908	PGN1837	*hypothetical protein PG_1908*	0.751373	2.13	3	-0.49	2.36	Y	Y	0.052108	-1.91	66413	1177771.5
PG1910	PGN1840	*rplQ ribosomal protein L17*	0.000007	154.6	90	0.78	7.93	R	R	0.000107	1.39	221254844	84194010.6
PG1911	PGN1841	*rpoA DNA-directed RNA polymerase alpha subunit*	0.293852	364.64	339.5	0.1	9.46	Y	R	0.001445	0.39	294643151	225134563
PG1912	PGN1842	*rpsD ribosomal protein S4*	0.000000	249.49	154	0.7	8.66	R	Y	0.014416	0.53	232454843	163023096
PG1913	PGN1843	*rpsK ribosomal protein S11*	0.051394	10.66	7	0.61	4.14	Y	G	0.008125	-4.23	2665066.5	10292093.8
PG1914	PGN1844	*rpsM ribosomal protein S13*	0.015347	63.97	37.5	0.77	6.66	Y	Y	0.161524	0.05	109327342	106060569
PG1916	PGN1846	*infA translation initiation factor IF-1*	0.348965	34.12	48	-0.49	6.36	Y	Y	0.019812	-0.73	5127043.5	8724750.08
PG1917	PGN1847	*map methionine aminopeptidase type I*	0.682945	36.25	35	0.05	6.15	Y	Y	0.048151	0.57	19383361	12930251.1
PG1918	PGN1848	*secY preprotein translocase SecY subunit*	0.786857	59.71	56	0.09	6.85	Y	Y	0.033883	-0.60	44385258	63999035.9
PG1919	PGN1849	*rplO ribosomal protein L15*	0.018536	99.16	66	0.59	7.37	Y	Y	0.064220	0.06	69006032.5	66159447.8
PG1920	PGN1850	*rpmD ribosomal protein L30*	0.000101	82.1	41.5	0.98	6.95	R	Y	0.070470	-0.20	83676045	98005710.2
PG1921	PGN1851	*rpsE ribosomal protein S5*	0.000004	74.63	35	1.09	6.78	R	R	0.003984	1.66	104546723	32617332.9
PG1922	PGN1852	*rplR ribosomal protein L18*	0.000000	180.19	81.5	1.14	8.03	R	R	0.000206	1.14	213545194	96702035.3
PG1923	PGN1853	*rplF ribosomal protein L6*	0.010615	208.97	155.5	0.43	8.51	Y	Y	0.097582	0.08	273099256	259426326
PG1924	PGN1854	*rpsH ribosomal protein S8*	0.720393	52.24	46.5	0.17	6.63	Y	Y	0.011407	-0.25	64416207.5	76927560.6
PG1925	PGN1855	*rpsN ribosomal protein S14*	0.751373	2.13	7	-1.72	3.19	Y	Y	0.018743	-7.10	66413	10660794.7
PG1926	PGN1856	*rplE ribosomal protein L5*	0.213146	271.88	296.5	-0.13	9.15	Y	Y	0.159862	-0.01	248507008	250360644
PG1927	PGN1857	*rplX ribosomal protein L24*	0.014708	76.77	53	0.53	7.02	Y	Y	0.161524	-0.01	98532529	105876906
PG1928	PGN1858	*rplN ribosomal protein L14*	0.128819	51.18	36	0.51	6.45	Y	Y	0.014353	1.41	28809569	10346899.1
PG1929	PGN1859	*rpsQ ribosomal protein S17*	0.250218	6.4	10.5	-0.71	4.08	Y	Y	0.067266	-1.76	30672646.5	7278525.4
PG1930	PGN1860	*rpmC ribosomal protein L29*	0.003969	12.79	5.5	1.22	4.19	R	Y	0.054253	0.20	12237550	1197596.08
PG1931	PGN1861	*rplP ribosomal protein L16*	0.000007	82.1	38	1.11	6.91	R	R	0.000505	1.30	136799112	55603720.1

TIGR	PgSgFn / Pg Spectral Count						PgSgFn / Pg Protein Intensity				
PGN	*q*-value	PgSgFn	Pg	log_2Ratio	log_2Sum	Regulation		*q*-value	log_2Ratio	PgSgFn	Pg
PG1932	0.000000	148.2	76.5	0.95	7.81	R	Y	0.010369	0.88	156758483	83626514.5
PGN1862	*rpsC ribosomal protein S3*										
PG1933	0.000000	134.34	56.5	1.25	7.58	R	Y	0.010105	1.02	84195719	40810463.2
PGN1863	*rplV ribosomal protein L22*										
PG1934	0.000000	93.82	33	1.51	6.99	R	R	0.004947	0.92	51522519.5	27912794
PGN1864	*rpsS ribosomal protein S19*										
PG1935	0.000005	65.04	28	1.22	6.54	R	R	0.004995	1.58	53306402	17715259.7
PGN1865	*rplB ribosomal protein L2*										
PG1936	0.007139	44.78	21.5	1.06	6.05	R	R	0.004415	1.07	24324769.5	11481420.3
PGN1866	*rplW ribosomal protein L23*										
PG1937	0.000000	316.66	203.5	0.64	9.02	R	R	0.008013	0.62	135610320	88373729.5
PGN1867	*rplD ribosomal protein L4*										
PG1938	0.000000	103.42	54	0.94	7.3	R	Y	0.040771	0.51	87274806	63248332.6
PGN1868	*rplC ribosomal protein L3*										
PG1939	0.000195	113.02	131.5	-0.22	7.93	G	Y	0.115558	0.13	109702358	100245678
PGN1869	*rpsJ ribosomal protein S10*										
PG1940	0.000000	1090.71	681.5	0.68	10.79	R	R	0.003109	0.47	842542931	608363170
PGN1870	*fusA translation elongation factor G*										
PG1941	0.000135	135.41	82.5	0.71	7.77	R	R	0.008458	0.64	220558288	141832374
PGN1871	*rpsG ribosomal protein S7*										
PG1944	0.779998	2.13	2.5	-0.23	2.21	Y	Y	0.052147	-1.73	66413	920849.15
PGN1874	*aroA 3-phosphoshikimate 1-carboxyvinyltransferase*										
PG1947	0.000000	6.4	38	-2.57	5.47	G	G	0.001876	-4.98	979782.5	10418504.3
PGN1877	*TPR domain protein*										
PG1948	0.000040	392.36	291	0.43	9.42	R	Y	0.052108	-0.16	199665401	224124566
PGN1878	*lipoprotein putative*										
PG1949	0.019015	552.29	467	0.24	9.99	Y	Y	0.027198	-0.08	358229903	379840362
PGN1880	*mdh malate dehydrogenase*										
PG1950	0.037442	2.13	14.5	-2.77	4.06	Y	G	0.004947	-7.05	66413	9023474.08
PGN1882	*membrane protein putative*										
PG1951	0.420278	214.3	188	0.19	8.65	Y	Y	0.146675	0.03	114038310	111395139
PGN1883	*glnS glutaminyl-tRNA synthetase*										
PG1952	0.148470	9.6	14	-0.54	4.56	Y	Y	0.082564	-3.55	5915048.5	9591000.98
PGN1884	*DedA family protein*										
PG1953	0.161227	25.59	40	-0.64	6.04	Y	G	0.000797	-0.71	26414185.5	43176323.6
PGN1885	*YitT family protein*										
PG1956	0.000117	56.51	100.5	-0.83	7.29	G	G	0.005006	-0.91	27448042.5	51382402.5
PGN1888	*abfT-2 4-hydroxybutyrate CoA-transferase*										
PG1959	0.238962	17.06	8.5	1.01	4.68	Y	Y	0.023533	2.03	20175360.5	4376314.68
PGN1890	*rpmG ribosomal protein L33*										
PG1960	0.014629	26.65	11.5	1.21	5.25	Y	Y	0.037877	0.40	40318142.5	29893885.5
PGN1891	*rpmB ribosomal protein L28*										
PG1961	0.324918	2.13	10.5	-2.3	3.66	Y	G	0.003742	-7.07	66413	9057518.85
PGN1892	*hypothetical protein PG_1961*										
PG1963	0.720026	2.13	7.5	-1.82	3.27	Y	G	0.007305	-5.46	66413	3040756.23
PGN1895	*Sua5/YciO/YrdC/YwlC family protein*										
PG1964	0.779998	2.13	6	-1.49	3.02	Y	G	0.002288	-6.58	66413	6359505.65
PGN1896	*bacterial sugar transferase*										

TIGR	PGN	Description	PgSgFn / Pg Spectral Count q-value	PgSgFn	Pg	log_2Ratio	log_2Sum	Regulation (Spectral Count)	Regulation (Protein Intensity)	PgSgFn / Pg Protein Intensity q-value	log_2Ratio	PgSgFn	Pg
PG1972	PGN1904	hemagglutinin protein HagB	0.000001	400.89	293.5	0.45	9.44	R	Y	0.139769	0.03	424423477	412068199
PG1974	PGN1905	hypothetical protein PG_1974	0.659176	2.13	7.5	-1.82	3.27	Y	Y	0.021119	-2.68	66413	485098.575
PG1975	PGN1906	hemagglutinin protein HagC	0.751373	2.13	3	-0.49	2.36	Y	Y	0.052108	-3.25	66413	7462572
PG1060	PGN1914	carboxyl-terminal protease	0.716626	109.82	116.5	-0.09	7.82	Y	Y	0.190624	-0.01	70723816	70890239
PG1175	PGN1916	ABC transporter ATP-binding protein putative	0.024778	170.59	128.5	0.41	8.22	Y	Y	0.190624	-0.01	124321636	124490549
PG1176	PGN1917	ABC transporter ATP-binding protein putative	0.001494	215.37	157	0.46	8.54	R	Y	0.094130	0.10	200608155	186104202
PG1178	PGN1918	hypothetical protein PG_1178	0.420012	165.26	192.5	-0.22	8.48	Y	G	0.000462	-0.53	74676286	108195275
PG1179	PGN1919	conserved hypothetical protein	0.274290	8.53	9.5	-0.16	4.17	Y	Y	0.040104	-3.73	4089757.5	9044701.85
PG1180	PGN1920	membrane protein putative	0.753108	163.13	148.5	0.14	8.28	Y	Y	0.011777	-0.18	86251626.5	97705880.5
PG1181	PGN1921	transcriptional regulator tetR family	0.773021	69.3	76	-0.13	7.18	Y	Y	0.013019	-0.34	61814700.5	78589343.1
PG1983	PGN1928	CRISPR-associated protein TM1791 family	0.685422	2.13	3.5	-0.72	2.49	Y	Y	0.052108	-1.86	66413	1427832.5
PG1984	PGN1929	hypothetical protein PG_1984	0.336653	6.4	13	-1.02	4.28	Y	G	0.004191	-4.68	2451414.5	13467201.3
PG1985	PGN1930	CRISPR-associated protein TM1792 family	0.003938	51.18	28.5	0.84	6.32	R	Y	0.052147	0.15	34229330	30800901.3
PG1986	PGN1931	CRISPR-associated protein TM1793 family	0.064843	30.92	51.5	-0.74	6.36	Y	Y	0.069213	-0.34	19938793.5	25589576.2
PG1987	PGN1932	CRISPR-associated protein TM1811 family	0.679564	61.84	57	0.12	6.89	Y	Y	0.020429	-0.54	12174883.5	17978212.2
PG1988	PGN1933	hypothetical protein PG_1988	0.660293	2.13	8	-1.91	3.34	Y	G	0.000003	-6.09	66413	4470390.1
PG1992	PGN1937	gidA glucose-inhibited division protein A	0.274290	2.13	10.5	-2.3	3.66	Y	G	0.005850	-5.12	66413	2374294.13
PG1993	PGN1938	uvrC excinuclease ABC C subunit	0.457936	6.4	11	-0.78	4.12	Y	Y	0.076556	-3.02	2679480.5	4510906
PG1994	PGN1939	dtd D-tyrosyl-tRNA(Tyr) deacylase	0.489278	15.99	9.5	0.75	4.67	Y	Y	0.055401	-0.49	7779399	11401018.7
PG1996	PGN1941	deoC deoxyribose-phosphate aldolase	0.779998	74.63	74.5	0	7.22	Y	G	0.009969	-0.45	47991143	65548763.4
PG1998	PGN1943	polyprenyl synthetase	0.000015	20.26	53.5	-1.4	6.2	G	Y	0.010656	-1.32	10231274.5	24238640.8
PG2001	PGN1946	lepB signal peptidase I	0.543202	56.51	45.5	0.31	6.67	Y	Y	0.150663	0.01	23858934	23621385.3
PG2002	PGN1947	dapB dihydrodipicolinate reductase	0.000002	2.13	26.5	-3.64	4.84	Y	G	0.000338	-7.73	66413	13979544.2
PG2003	PGN1948	dgt deoxyguanosinetriphosphate triphosphohydrolase	0.037030	7.46	20.5	-1.46	4.81	Y	Y	0.046400	-3.45	4112177.5	8642341.35

TIGR	PgSgFn / Pg Spectral Count					Regulation		PgSgFn / Pg Protein Intensity			
PGN	*q*-value	PgSgFn	Pg	log_2Ratio	log_2Sum			*q*-value	log_2Ratio	PgSgFn	Pg
PG2008	0.002096	13.86	29	-1.07	5.42	G	G	0.007643	-4.21	2395208	11226727.7
PGN1953	*TonB-dependent receptor putative*										
PG2010	0.120637	170.59	196	-0.2	8.52	Y	Y	0.014416	-0.70	93241069	153436269
PGN1955	*phosphomannomutase putative*										
PG2014	0.006294	15.99	13.5	0.24	4.88	R	Y	0.053384	-3.41	3398159.5	6710452.83
PGN1960	*cas1 CRISPR-associated protein Cas1*										
PG2015	0.692989	2.13	3.5	-0.72	2.49	Y	Y	0.053629	-1.32	66413	528416.95
PGN1961	*cas4 CRISPR-associated protein Cas4*										
PG2020	0.015659	40.52	62	-0.61	6.68	Y	G	0.009634	-0.62	29570619	44725268.5
PGN1966	*CRISPR-associated protein TM1814 family*										
PG2021	0.751373	2.13	7	-1.72	3.19	Y	Y	0.012891	-3.20	66413	658919.45
PGN1967	*conserved hypothetical protein*										
PG2022	0.001214	2.13	19	-3.16	4.4	Y	G	0.004394	-7.52	66413	12382281.7
PGN1968	*hypothetical protein PG_2022*										
PG2023	0.008866	21.32	43.5	-1.03	6.02	G	Y	0.063017	-0.31	13119018	16010004
PGN1969	*fmt methionyl-tRNA formyltransferase*										
PG2026	0.066043	11.73	15	-0.35	4.74	Y	Y	0.083272	-3.71	7317207	12183251
PGN1973	*phosphoglycerate mutase family protein*										
PG2029	0.000000	638.65	479	0.41	10.13	R	Y	0.017132	-0.20	563989676	646264294
PGN1976	*hypothetical protein PG_2029*										
PG2031	0.543202	2.13	9	-2.08	3.48	Y	G	0.000705	-6.36	66413	5401870.53
PGN1978	*hypothetical protein PG_2031*										
PG2033	0.487462	58.64	68.5	-0.22	6.99	Y	G	0.006530	-0.38	26484639	34469447.4
PGN1980	*gltD glutamate synthase small subunit*										
PG2034	0.018560	33.05	57.5	-0.8	6.5	Y	Y	0.011984	-0.61	19134022.5	29387311.2
PGN1981	*oxidoreductase FAD-binding putative*										
PG2041	0.000750	2.13	20	-3.23	4.47	Y	G	0.001602	-7.29	66413	10357109.1
PGN1987	*hypothetical protein PG_2041*										
PG2042	0.102154	2.13	13	-2.61	3.92	Y	G	0.004884	-7.10	66413	9286634.63
PGN1988	*thioredoxin family protein*										
PG2043	0.006122	8.53	23	-1.43	4.98	G	Y	0.070470	-3.56	5132375.5	9899330.5
PGN1989	*conserved hypothetical protein TIGR00486*										
PG2044	0.130076	113.02	97	0.22	7.71	Y	Y	0.159862	0.04	91163476	85590563.3
PGN1990	*conserved hypothetical protein*										
PG2046	0.779998	2.13	2.5	-0.23	2.21	Y	Y	0.052192	-1.55	66413	935077
PGN1991	*conserved hypothetical protein*										
PG2048	0.000037	2.13	23.5	-3.46	4.68	Y	G	0.001242	-6.22	66413	4943238.33
PGN1993	*hypothetical protein PG_2048*										
PG2049	0.522042	7.46	10	-0.42	4.13	Y	Y	0.060576	-3.01	2417562	6791311.73
PGN1994	*hypothetical protein PG_2049*										
PG2050	0.000000	592.8	340.5	0.8	9.87	R	Y	0.062174	0.17	454854962	406919387
PGN1995	*hypothetical protein PG_2050*										
PG2052	0.751373	53.31	58.5	-0.13	6.8	Y	G	0.000137	-0.74	21452900	35943225
PGN1996	*dapA dihydrodipicolinate synthase*										
PG2053	0.751373	2.13	7	-1.72	3.19	Y	G	0.003242	-6.76	66413	7282581.5
PGN1997	*bioD dethiobiotin synthase*										
PG2054	0.175055	104.49	81.5	0.36	7.54	Y	R	0.005100	0.51	139510382	98101427
PGN1998	*lipoprotein PG3*										

TIGR	PgSgFn / Pg Spectral Count							PgSgFn / Pg Protein Intensity			
PGN	*q*-value	PgSgFn	Pg	log_2Ratio	log_2Sum	Regulation		*q*-value	log_2Ratio	PgSgFn	Pg
PG2055	0.000021	505.37	579.5	-0.2	10.08	G	Y	0.099350	0.10	247119707	232628439
PGN1999	*dihydroorotate dehydrogenase family protein*										
PG0054	0.007772	7.46	23	-1.62	4.93	G	G	0.007267	-4.53	3651532.5	14866260.6
PGN2003	*recJ single-stranded-DNA-specific exonuclease RecJ*										
PG0056	0.000000	56.51	155.5	-1.46	7.73	G	G	0.009093	-0.70	35232930	57855964.6
PGN2005	*hypothetical protein PG_0056*										
PG0057	0.102520	46.91	30	0.64	6.27	Y	G	0.003925	-0.93	10654428.5	20009402.7
PGN2006	*pncB nicotinate phosphoribosyltransferase*										
PG0058	0.214806	8.53	11.5	-0.43	4.32	Y	Y	0.130581	-1.99	3853200	2621307.7
PGN2007	*nadD nicotinate (nicotinamide) nucleotide adenylyltransferase*										
PG0059	0.074014	2.13	13.5	-2.66	3.97	Y	G	0.002733	-7.14	66413	9449248.95
PGN2008	*hypothetical protein PG_0059*										
PG0061	0.000003	2.13	26	-3.61	4.81	Y	G	0.002069	-5.98	66413	4208187
PGN2010	*yngK-1 yngK protein*										
PG0063	0.019015	55.44	82	-0.56	7.1	Y	G	0.001018	-0.55	29714679.5	43333857.2
PGN2012	*outer membrane efflux protein*										
PG0064	0.753725	73.57	82.5	-0.17	7.29	Y	Y	0.025864	-0.37	38719086.5	50591089
PGN2013	*heavy metal efflux pump CzcA family*										
PG0069	0.000330	33.05	66.5	-1.01	6.64	G	G	0.004225	-1.64	6702207	21133011.5
PGN2017	*conserved hypothetical protein*										
PG0070	0.293852	113.02	139	-0.3	7.98	Y	Y	0.189687	-0.08	123457042	124340870
PGN2018	*lpxA acyl-(acyl-carrier-protein)-UDP-N-acetylglucosamine acyltransferase*										
PG0071	0.070322	232.43	191	0.28	8.73	Y	Y	0.029655	0.17	158957831	141358729
PGN2019	*UDP-3-O-acyl-GlcNAc deacetylase/beta-hydroxyacyl-[acyl carrier protein] dehydratase FabZ*										
PG0072	0.123913	9.6	15.5	-0.69	4.65	Y	Y	0.140843	-2.96	13095121.5	9569772.68
PGN2020	*lpxD UDP-3-O-[3-hydroxymyristoyl] glucosamine N-acyltransferase*										
PG0073	0.000003	36.25	81	-1.16	6.87	G	G	0.008013	-1.60	18036049.5	56275583.9
PGN2021	*orotidine 5'-monophosphate decarboxylase*										
PG0074	0.039231	2.13	14.5	-2.77	4.06	Y	G	0.005850	-6.63	66413	6798445.45
PGN2022	*prfA peptide chain release factor 1*										
PG0075	0.773021	38.38	33	0.22	6.16	Y	Y	0.071070	-0.07	18875362	19857998
PGN2023	*phosphoribosylformylglycinamidine cyclo-ligase putative*										
PG0076	0.339867	25.59	38	-0.57	5.99	Y	G	0.003925	-1.25	8922888	20550311.2
PGN2024	*N-acetylmuramoyl-L-alanine amidase family 4*										
PG0078	0.423387	2.13	9.5	-2.16	3.54	Y	G	0.000770	-4.65	66413	1654452.73
PGN2025	*conserved hypothetical protein*										
PG0079	0.720026	24.52	30.5	-0.31	5.78	Y	Y	0.013019	-0.51	13733489	19785107.4
PGN2026	*abortive infection protein putative*										
PG0081	0.327141	2.13	10.5	-2.3	3.66	Y	G	0.000008	-5.40	66413	2771212.03
PGN2028	*hypothetical protein PG_0081*										
PG0082	0.345160	2.13	10	-2.23	3.6	Y	G	0.007499	-6.56	66413	6541651.33
PGN2029	*hypothetical protein PG_0082*										
PG0083	0.600049	2.13	8.5	-2	3.41	Y	G	0.005822	-5.90	66413	4094680.98
PGN2030	*hypothetical protein PG_0083*										
PG0084	0.001057	12.79	30	-1.23	5.42	G	G	0.007090	-4.57	4801173.5	20313189.6
PGN2031	*sda L-serine dehydratase iron-sulfur-dependent single chain form*										
PG0086	0.786859	78.9	78	0.02	7.29	Y	Y	0.063017	0.27	65733379.5	53068337.6
PGN2033	*ATP-dependent RNA helicase DEAD/DEAH box family*										

TIGR	PgSgFn / Pg Spectral Count							PgSgFn / Pg Protein Intensity			
PGN	*q*-value	PgSgFn	Pg	log_2Ratio	log_2Sum	Regulation		*q*-value	log_2Ratio	PgSgFn	Pg
PG0087	0.604563	22.39	15	0.58	5.22	Y	Y	0.026006	0.38	11789638.5	9189558.08
PGN2034	*SIS domain protein*										
PG0088	0.634798	2.13	8	-1.91	3.34	Y	Y	0.017839	-4.91	66413	2295229.18
PGN2035	*peptidase M16 family*										
PG0090	0.000000	735.67	561	0.39	10.34	R	Y	0.086344	0.12	1.0448e+09	959449222
PGN2037	*Dps family protein*										
PG0091	0.779998	2.13	6	-1.49	3.02	Y	Y	0.014353	-6.14	66413	5198757
PGN2038	*transporter putative*										
PG0092	0.608229	2.13	8.5	-2	3.41	Y	G	0.003861	-4.38	66413	1398459.65
PGN2039	*transporter putative*										
PG0093	0.033250	10.66	19.5	-0.87	4.91	Y	Y	0.055854	-4.10	9427385	17704887.1
PGN2040	*HlyD family secretion protein*										
PG0094	0.000644	60.77	97	-0.67	7.3	G	Y	0.014959	-0.77	30281790	49870406.1
PGN2041	*outer membrane efflux protein putative*										
PG0095	0.461600	29.85	40.5	-0.44	6.14	Y	Y	0.149560	-0.55	11203537	12812205.7
PGN2042	*mutS DNA mismatch repair protein MutS*										
PG0097	0.000000	27.72	156	-2.49	7.52	G	G	0.004061	-1.65	7774283.5	24665816.7
PGN2043	*conserved hypothetical protein TIGR01033*										
PG0099	0.408616	77.83	96.5	-0.31	7.45	Y	Y	0.171340	-0.01	63339718	61121513.1
PGN2045	*pheT phenylalanyl-tRNA synthetase beta subunit*										
PG2072	0.383939	2.13	10	-2.23	3.6	Y	G	0.007304	-5.60	66413	3349148.5
PGN2050	*UvrD/REP helicase domain protein*										
PG2070	0.000115	2.13	22	-3.37	4.59	Y	G	0.000503	-6.72	66413	6942008.15
PGN2052	*hypothetical protein PG_2070*										
PG2069	0.000000	2.13	30.5	-3.84	5.03	Y	G	0.004394	-7.46	66413	11891090.5
PGN2053	*oxidoreductase short chain dehydrogenase/reductase family*										
PG2068	0.774795	49.04	55	-0.17	6.7	Y	G	0.001091	-0.66	43647173.5	68648890.9
PGN2054	*tagD glycerol-3-phosphate cytidylyltransferase*										
PG2067	0.009704	50.11	70.5	-0.49	6.91	G	G	0.004884	-0.71	17859577	29396163.2
PGN2055	*pdxA pyridoxal phosphate biosynthetic protein PdxA*										
PG2066	0.000026	28.79	65	-1.17	6.55	G	G	0.001876	-1.16	13488378.5	29822596.8
PGN2056	*lipoprotein putative*										
PG2065	0.779998	2.13	6	-1.49	3.02	Y	G	0.000107	-5.82	66413	3716387.5
PGN2057	*conserved hypothetical protein TIGR00048*										
PG2062	0.012889	30.92	56	-0.86	6.44	Y	G	0.005934	-0.91	21928101.5	40467281.9
PGN2060	*hisS histidyl-tRNA synthetase*										
PG2061	0.175055	2.13	12	-2.49	3.82	Y	G	0.000584	-6.44	66413	5731359.98
PGN2061	*folA dihydrofolate reductase*										
PG2060	0.770282	27.72	23	0.27	5.66	Y	Y	0.168269	0.11	18920534.5	18087549.3
PGN2062	*thyA thymidylate synthase*										
PG2197	0.039231	2.13	14.5	-2.77	4.06	Y	G	0.006427	-7.87	66413	16091032.2
PGN2064	*conserved hypothetical protein*										
PG2024	0.000000	1923.4	1976.5	-0.04	11.93	G	R	0.007107	0.27	2.6133e+09	2.1735e+09
PGN2065	*hagE hemagglutinin protein HagE*										
PG2199	0.430466	2.13	9.5	-2.16	3.54	Y	G	0.009306	-5.38	66413	2938118.5
PGN2066	*ABC transporter ATP-binding protein putative*										
PG2200	0.779998	20.26	19.5	0.06	5.31	Y	Y	0.135130	-0.02	10354916	8570897.38
PGN2067	*TPR domain protein*										

TIGR	PGN	Description	PgSgFn / Pg Spectral Count q-value	PgSgFn	Pg	log$_2$Ratio	log$_2$Sum	Regulation (Spectral Count)	Regulation (Protein Intensity)	PgSgFn / Pg Protein Intensity q-value	log$_2$Ratio	PgSgFn	Pg
PG2201	PGN2068	def polypeptide deformylase	0.035356	23.46	12	0.97	5.15	Y	Y	0.030329	1.28	12611192.5	4739298.63
PG2204	PGN2070	hypothetical protein PG_2204	0.420278	188.72	215.5	-0.19	8.66	Y	Y	0.075986	-0.12	115552638	125323369
PG2205	PGN2071	panE 2-dehydropantoate 2-reductase	0.751373	31.99	36.5	-0.19	6.1	Y	G	0.001367	-0.72	15202092	25053538.4
PG2206	PGN2072	ABC transporter ATP-binding protein	0.011536	44.78	68	-0.6	6.82	Y	Y	0.022204	-0.55	29517930	42508754.2
PG2207	PGN2073	conserved domain protein	0.203332	95.96	123.5	-0.36	7.78	Y	G	0.009241	-0.36	84556584	108314532
PG2210	PGN2075	uvrA-2 excinuclease ABC A subunit	0.130489	6.4	15.5	-1.28	4.45	Y	Y	0.172311	-2.66	4050837.5	4489217.18
PG2213	PGN2077	nitrite reductase-related protein	0.423387	2.13	9.5	-2.16	3.54	Y	G	0.001053	-4.78	66413	1819694.78
PG2215	PGN2079	manC mannose-1-phosphate guanylyltransferase	0.397614	90.63	96	-0.08	7.54	Y	Y	0.027084	0.26	71157682.5	59843078
PG2216	PGN2080	hypothetical protein PG_2216	0.692989	57.57	49.5	0.22	6.74	Y	Y	0.051355	-0.76	31536331.5	51281969.1
PG2217	PGN2081	dxs deoxyxylulose-5-phosphate synthase	0.003541	19.19	41.5	-1.11	5.92	G	G	0.001647	-1.32	8728342	21772864.6
PG2218	PGN2082	trkA potassium uptake protein TrkA	0.779998	24.52	28.5	-0.22	5.73	Y	G	0.004902	-0.84	8473792.5	15218141.9
PG2221	PGN2085	MiaB-like tRNA modifying enzyme	0.147331	9.6	9.5	0.02	4.26	Y	Y	0.191557	-2.34	3028169.5	2986522.98
PG2222	PGN2086	acyltransferase HtrB/MsbB family	0.400806	2.13	5	-1.23	2.83	Y	Y	0.052108	-2.26	66413	1898850
PG2227	PGN2091	hypothetical protein PG_2227	0.720026	2.13	7.5	-1.82	3.27	Y	G	0.000176	-6.40	66413	5557448.95
PG0183		lipoprotein putative	0.786190	24.52	26.5	-0.11	5.67	Y	G	0.000238	-0.99	11799765	23427742.2
PG0184		ISPg1 transposase	0.021100	111.95	77	0.54	7.56	Y	Y	0.135130	-0.11	52890190.5	56312697.8
PG0460		ISPg1 transposase	0.000000	2.13	32	-3.91	5.09	Y	G	0.001876	-7.89	66413	15815432.5
PG0718		conserved hypothetical protein	0.784897	2.13	5.5	-1.37	2.93	Y	Y	0.010832	-7.12	66413	9991080.5
PG0770		hypothetical protein PG_0770	0.600049	2.13	4	-0.91	2.62	Y	Y	0.052108	-2.56	66413	3787101.5
PG0810		hypothetical protein PG_0810	0.600049	2.13	4	-0.91	2.62	Y	Y	0.052108	-2.06	66413	1877864.5
PG0826		transcriptional regulator AraC family	0.725943	2.13	7	-1.72	3.19	Y	G	0.000828	-6.58	66413	6312089.78
PG0833		conserved hypothetical protein	0.685422	2.13	3.5	-0.72	2.49	Y	Y	0.053020	-1.30	66413	663248
PG0851		conserved hypothetical protein	0.600049	2.13	4	-0.91	2.62	Y	Y	0.052108	-2.56	66413	3782649.5
PG0854		hypothetical protein PG_0854	0.604563	2.13	4	-0.91	2.62	Y	Y	0.052108	-1.90	66413	1166788.05

TIGR / *PGN*	PgSgFn / Pg Spectral Count							PgSgFn / Pg Protein Intensity			
	q-value	PgSgFn	Pg	log_2Ratio	log_2Sum	Regulation		*q*-value	log_2Ratio	PgSgFn	Pg
PG1025	0.543202	2.13	9	-2.08	3.48	Y	G	0.000995	-4.71	66413	1731751.78
	conserved domain protein										
PG1530	0.604563	2.13	4	-0.91	2.62	Y	Y	0.056249	-1.01	66413	349166
	conserved domain protein										
PG1630	0.000000	437.14	391	0.16	9.69	R	Y	0.050907	1.46	232235660	57156757.1
	hypothetical protein PG_1630										
PG1863	0.240620	2.13	11	-2.37	3.71	Y	G	0.001728	-5.70	66413	3442877.63
	hypothetical protein PG_1863										

TIGR	PGN	Description	Sec Pg in Pg	Sec Pg in PgSgFn	Sec Pg in Pg Mean Sc	Sec Pg in Pg Mean Intensity	Sec Pg in PgSgFn Mean Sc	Sec Pg in PgSgFn Mean Intensity
PG0026	PGN0022	hypothetical protein PG_0026	●		7	1258696		
PG0027	PGN0023	hypothetical protein PG_0027	●	●	19	4895226	5	1784808
PG0034	PGN0033	trx thioredoxin		●			4	1024073
PG0042	PGN0038	glyA serine hydroxymethyltransferase	●		4	800313		
PG2173	PGN0122	omp28 outer membrane lipoprotein Omp28	●	●	4	914606	4	735199
PG2167	PGN0128	immunoreactive 53 kDa antigen PG123	●	●	25	9615711	5	915560
PG2168	PGN0129	hypothetical protein PG_2168	●		9	2963937		
PG2094	PGN0145	conserved domain protein		●			3	327127
PG2124	PGN0173	gapA glyceraldehyde 3-phosphate dehydrogenase type I	●	●	15	2972887	6	2038238
P13793IFMA_PORGI	PGN0180	major fimbrillin A, Porphyromonas gingivalis 33277	●	●	233	128837846	130	73554754
PG2134	PGN0183	lipoprotein putative	●		3	1048602		
PG2157	PGN0202	glutamine cyclotransferase-related protein	●	●	4	974772	3	778146
PG0121	PGN0235	hup-1 DNA-binding protein HU	●		5	1016575		
PG0159	PGN0271	pepO endopeptidase PepO	●		5	933854		
PG0179	PGN0288	hypothetical protein PG_0179						
PG0180	PGN0289	lipoprotein putative	●		4	1285096		
PG0181	PGN0290	immunoreactive 32 kDa antigen PG49	●	●	8	1489527	4	1064556
PG0182	PGN0291	von Willebrand factor type A domain protein	●		5	1075448		
PG0185	PGN0293	ragA ragA protein	●	●	30	9080269	18	3023025
PG0192	PGN0300	ompH-1 cationic outer membrane protein OmpH						
PG0193	PGN0301	ompH-2 cationic outer membrane protein OmpH	●		5	402857		
PG0196	PGN0303	peptidase M16 family	●	●	16	3854188	3	729732
PG0217	PGN0322	hypothetical protein PG_0217	●		5	838286		
PG0232	PGN0335	zinc carboxypeptidase putative	●	●	18	3528401	6	1813666

TIGR	PGN	Description	Sec Pg in Pg	Sec Pg in PgSgFn	Sec Pg in Pg Mean Sc	Sec Pg in Pg Mean Intensity	Sec Pg in PgSgFn Mean Sc	Sec Pg in PgSgFn Mean Intensity
PG1729	*PGN0388*	*thiol peroxidase*	●		6	814128		
PG1726	*PGN0391*	*PDZ domain protein*	●		10	2267288		
PG1676	*PGN0434*	*pckA phosphoenolpyruvate carboxykinase (ATP)*	●	●	11	2219213	15	3544761
PG1626	*PGN0477*	*hypothetical protein PG_1626*	●		5	388616		
PG1604	*PGN0509*	*immunoreactive 84 kDa antigen PG93*	●		3	632877		
PG1553	*PGN0556*	*CobN/magnesium chelatase family protein*	●		8	955553		
PG1552	*PGN0557*	*hmuR TonB-dependent receptor HmuR*	●		10	1294932		
PG1551	*PGN0558*	*hmuY hmuY protein*	●	●	28	14238772	19	6737550
PG1545	*PGN0564*	*sodB superoxide dismutase Fe-Mn*	●		8	3715108		
PG1286	*PGN0604*	*ftn ferritin*	●	●	12	8110101	4	969569
PG1278	*PGN0612*	*serC phosphoserine aminotransferase*	●	●	19	5504974	47	13725683
PG0602	*PGN0645*	*hypothetical protein PG_0602*	●		3	775403		
PG0611	*PGN0654*	*lipoprotein putative*		●			4	722396
PG0613	*PGN0656*	*hypothetical protein PG_0613*	●		6	1484985		
PG0614	*PGN0657*	*hypothetical protein PG_0614*	●		12	2146675		
PG0616	*PGN0659*	*thioredoxin putative*	●	●	35	10246182	22	5568320
PG0623	*PGN0665*	*tpiA triosephosphate isomerase*	●	●	5	985726	13	3498261
PG0654	*PGN0693*	*hypothetical protein PG_0654*	●	●	8	2426924	4	1267833
PG0668	*PGN0704*	*TonB-dependent receptor*	●		4	657170		
PG0669	*PGN0705*	*fetB heme-binding protein FetB*	●	●	10	3469796	11	3118385
PG0687	*PGN0723*	*sucD succinate-semialdehyde dehydrogenase*	●	●	21	3715701	32	9053154
PG0689	*PGN0724*	*4hbD NAD-dependent 4-hydroxybutyrate dehydrogenase*	●		6	1260288		
PG0690	*PGN0725*	*abfT-1 4-hydroxybutyrate CoA-transferase*	●		4	552813		
PG0692	*PGN0727*	*abfD 4-hydroxybutyryl-CoA dehydratase*	●		15	3789281		

TIGR / *PGN*	Sec Pg in Pg	Sec Pg in PgSgFn	Sec Pg in Pg: Mean Sc	Sec Pg in Pg: Mean Intensity	Sec Pg in PgSgFn: Mean Sc	Sec Pg in PgSgFn: Mean Intensity
PG0694	●	●	17	4407306	5	1273947
PGN0728	*immunoreactive 42 kDa antigen PG33*					
PG0695	●	●	15	4312280	8	1930239
PGN0729	*immunoreactive 43 kDa antigen PG32*					
PG0707	●		8	1058462		
PGN0741	*TonB-dependent receptor putative*					
PG0724	●	●	4	859389	10	1935290
PGN0756	*prolyl oligopeptidase family protein*					
PG0766	●		4	612088		
PGN0792	*pnpA polyribonucleotide nucleotidyltransferase*					
PG0769	●	●	7	2514191	6	1984767
PGN0795	*fibronectin type III domain protein*					
PG0782	●	●	8	2135357	4	931078
PGN0806	*MotA/TolQ/ExbB proton channel family protein*					
PG0802	●	●	4	557449	4	682986
PGN0826	*pdhD alpha keto acid dehydrogenase complex E3 component lipoamide dehydrogenase*					
PG1374	●	●	12	2777827	5	869264
PGN0852	*immunoreactive 47 kDa antigen PG97*					
PG1385	●	●	9	2960748	3	606775
PGN0876	*TPR domain protein*					
PG1401	●		5	800063		
PGN0880	*beta-eliminating lyase*					
PG1421	●		5	1205541		
PGN0895	*ferredoxin 4Fe-4S*					
PG1424	●	●	41	23669897	22	8435568
PGN0898	*peptidylarginine deiminase*					
PG1427	●	●	6	3575179	4	2254971
PGN0900	*thiol protease/hemagglutinin PrtT precursor putative*					
PG1210	●	●	13	2506937	10	2184907
PGN0914	*peptidase M24 family*					
PG0950						
PGN1000	*gcvH glycine cleavage system H protein*					
PG0933	●		4	663176		
PGN1014	*translation elongation factor G putative*					
PG0903						
PGN1038	*conserved hypothetical protein*					
PG0890		●			4	968954
PGN1049	*alkaline phosphatase putative*					
PG1290	●	●	9	3654196	8	2929915
PGN1080	*ilvE branched-chain amino acid aminotransferase*					
PG1297	●		3	596100		
PGN1088	*rpsA ribosomal protein S1*					
PG1302	●		3	643743		
PGN1091	*hypothetical protein PG_1302*					
PG1305		●			10	2135945
PGN1094	*gcvP glycine cleavage system P protein*					
PG1313	●		5	856860		
PGN1103	*dipeptidase-related protein*					

TIGR	PGN	Description	Sec Pg in Pg	Sec Pg in PgSgFn	Sec Pg in Pg: Mean Sc	Sec Pg in Pg: Mean Intensity	Sec Pg in PgSgFn: Mean Sc	Sec Pg in PgSgFn: Mean Intensity
PG1327	PGN1116	*aminotransferase putative*	●	●	5	922699	4	1177884
PG1341	PGN1129	*hypothetical protein PG_1341*	●	●	6	4098009	3	1176068
PG1084	PGN1181	*thioredoxin family protein*	●	●	8	1221973	4	645667
PG1134	PGN1232	*trxB thioredoxin reductase*						
PG2188	PGN1272	*lysA diaminopimelate decarboxylase*		●			3	718495
PG1030	PGN1321	*hypothetical protein PG_1030*	●		3	364700		
PG1028	PGN1323	*TPR domain protein*	●		10	2892304		
PG1017	PGN1338	*ppdK pyruvate phosphate dikinase*	●		8	1033515		
PG1004	PGN1349	*prolyl oligopeptidase family protein*	●	●	13	2876468	10	2321517
PG1232	PGN1367	*gdh glutamate dehydrogenase NAD-specific*	●	●	37	17334014	175	104912972
PG1235	PGN1370	*epimerase/reductase putative*	●		4	682611		
PG1269	PGN1401	*pruA delta-1-pyrroline-5-carboxylate dehydrogenase*	●		4	443754		
PG0558	PGN1412	*purine nucleoside phosphorylase I inosine and guanosine-specific*	●	●	9	1992694	8	2362422
PG0553	PGN1416	*extracellular protease putative*	●	●	16	4635986	7	2261568
PG0537	PGN1434	*pepD-2 aminoacyl-histidine dipeptidase*	●	●	15	3395522	7	1660704
PG0506	PGN1466	*prtRII arginine-specific cysteine proteinase*	●	●	104	88812154	49	71045513
PG0495	PGN1476	*hypothetical protein PG_0495*	●	●	48	23735721	36	19838653
PG0491	PGN1479	*conserved hypothetical protein*	●		11	2590147		
PG0449	PGN1513	*TPR domain protein*	●		5	907467		
PG0429	PGN1530	*pyruvate synthase*	●		5	529390		
PG0411	PGN1556	*hemagglutinin putative*	●	●	12	3109808	10	3165978
PG0393	PGN1572	*rplL ribosomal protein L7/L12*	●	●	9	4817575	9	3244571
PG0390	PGN1575	*rplK ribosomal protein L11*	●		3	674660		
PG0387	PGN1578	*tuf translation elongation factor Tu*	●		14	4952070		

TIGR	*PGN*	Description	Sec Pg in Pg	Sec Pg in PgSgFn	Sec Pg in Pg Mean Sc	Sec Pg in Pg Mean Intensity	Sec Pg in PgSgFn Mean Sc	Sec Pg in PgSgFn Mean Intensity
PG0350	*PGN1611*	*internalin-related protein*	●	●	19	5946754	11	3366335
PG0343	*PGN1618*	*megL methionine gamma-lyase*	●	●	3	542694	4	985838
PG1755	*PGN1695*	*fbaB fructose-bisphosphate aldolase class I*	●	●	14	2246413	11	2417149
PG1762	*PGN1702*	*secDF protein-export membrane protein SecD/protein-export membrane protein SecF*	●		11	1731191		
PG1764	*PGN1704*	*fabF 3-oxoacyl-(acyl-carrier-protein) synthase II*	●		6	1057309		
PG1837	*PGN1733*	*hemagglutinin protein HagA*	●	●	53	56911019	34	44174273
PG1835	*PGN1735*	*lipoprotein putative*	●		3	292394		
PG1824	*PGN1743*	*eno enolase*	●		3	355781		
PG1823	*PGN1744*	*hypothetical protein PG_1823*						
PG1816	*PGN1749*	*NAD(P)H dehydrogenase quinone family putative*		●			4	674206
PG1812	*PGN1753*	*2-oxoglutarate oxidoreductase alpha subunit*	●		6	941448		
PG1798	*PGN1767*	*immunoreactive 46 kDa antigen PG99*	●		5	964806		
PG1788	*PGN1777*	*cysteine peptidase putative*	●		14	4114038		
PG1847	*PGN1780*	*endoribonuclease L-PSP putative*						
PG1858	*PGN1791*	*flavodoxin*	●	●	35	10096785	13	4589300
PG1872	*PGN1800*	*hutU urocanate hydratase*		●			10	2384560
PG1881	*PGN1808*	*hypothetical protein PG_1881*	●	●	13	1105780	7	1473415
PG1940	*PGN1870*	*fusA translation elongation factor G*	●		4	378205		
PG1948	*PGN1878*	*lipoprotein putative*	●		5	821945		
PG1949	*PGN1880*	*mdh malate dehydrogenase*	●		3	333410		
PG1996	*PGN1941*	*deoC deoxyribose-phosphate aldolase*						
PG0071	*PGN2019*	*UDP-3-O-acyl-GlcNAc deacetylase/beta-hydroxyacyl-[acyl carrier protein] dehydratase FabZ*	●	●	6	1088701	5	1346533
PG0073	*PGN2021*	*orotidine 5'-monophosphate decarboxylase*						
PG0090	*PGN2037*	*Dps family protein*	●	●	5	1166140	4	1074940

TIGR / PGN	Sec Pg in Pg	Sec Pg in PgSgFn	Sec Pg in Pg Mean Sc	Sec Pg in Pg Mean Intensity	Sec Pg in PgSgFn Mean Sc	Sec Pg in PgSgFn Mean Intensity
PG0094	●		4	960984		
PGN2041	*outer membrane efflux protein putative*					
PG2024	●	●	82	24114809	39	12240819
PGN2065	*hagE hemagglutinin protein HagE*					
PG2216	●		4	1260414		
PGN2080	*hypothetical protein PG_2216*					
PG0770						
	hypothetical protein PG_0770					
PG1630	●		6	526292		
	hypothetical protein PG_1630					

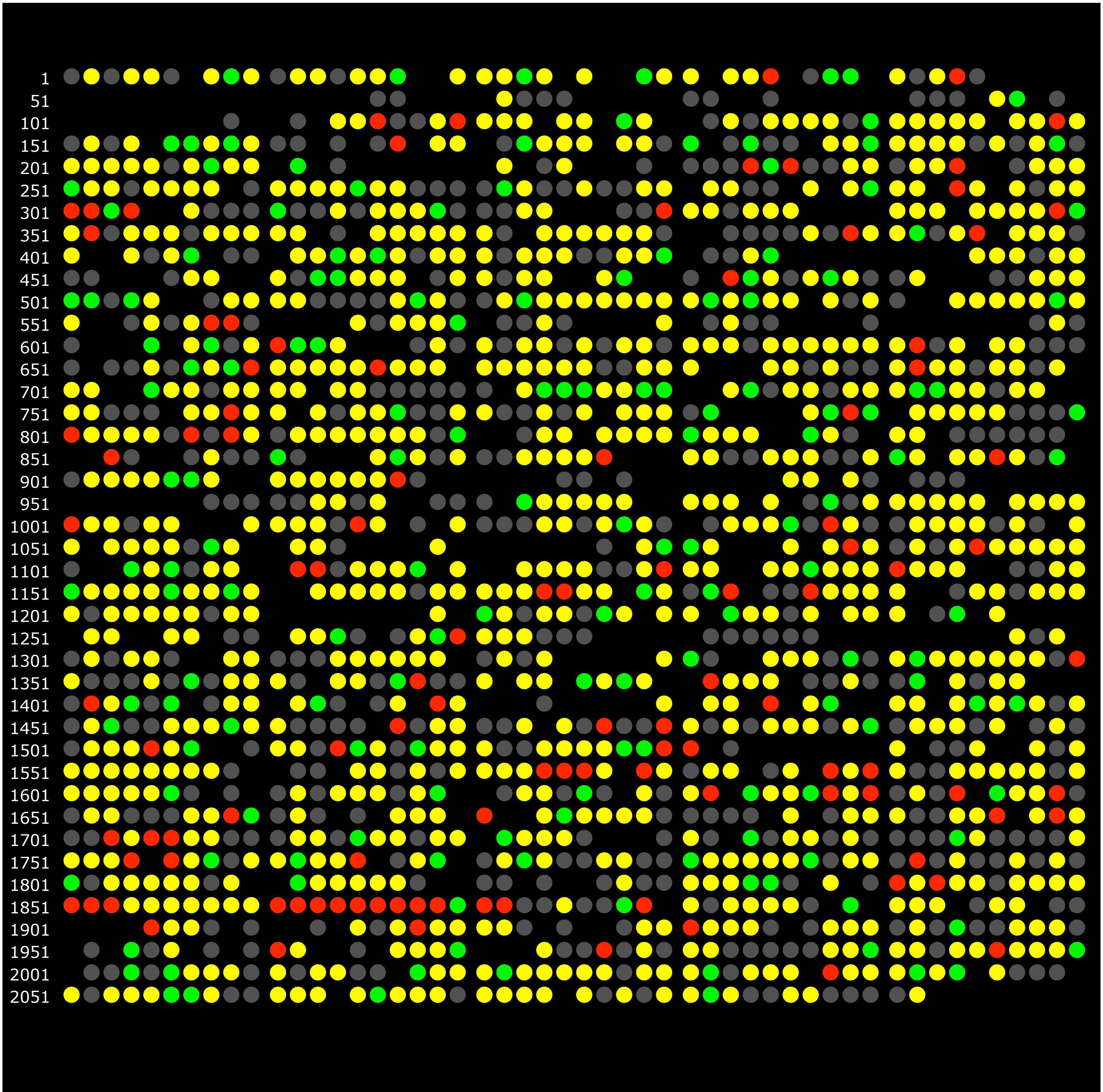

Figure SF1. PgSgFn / Pg : Spectral Count (PGN)

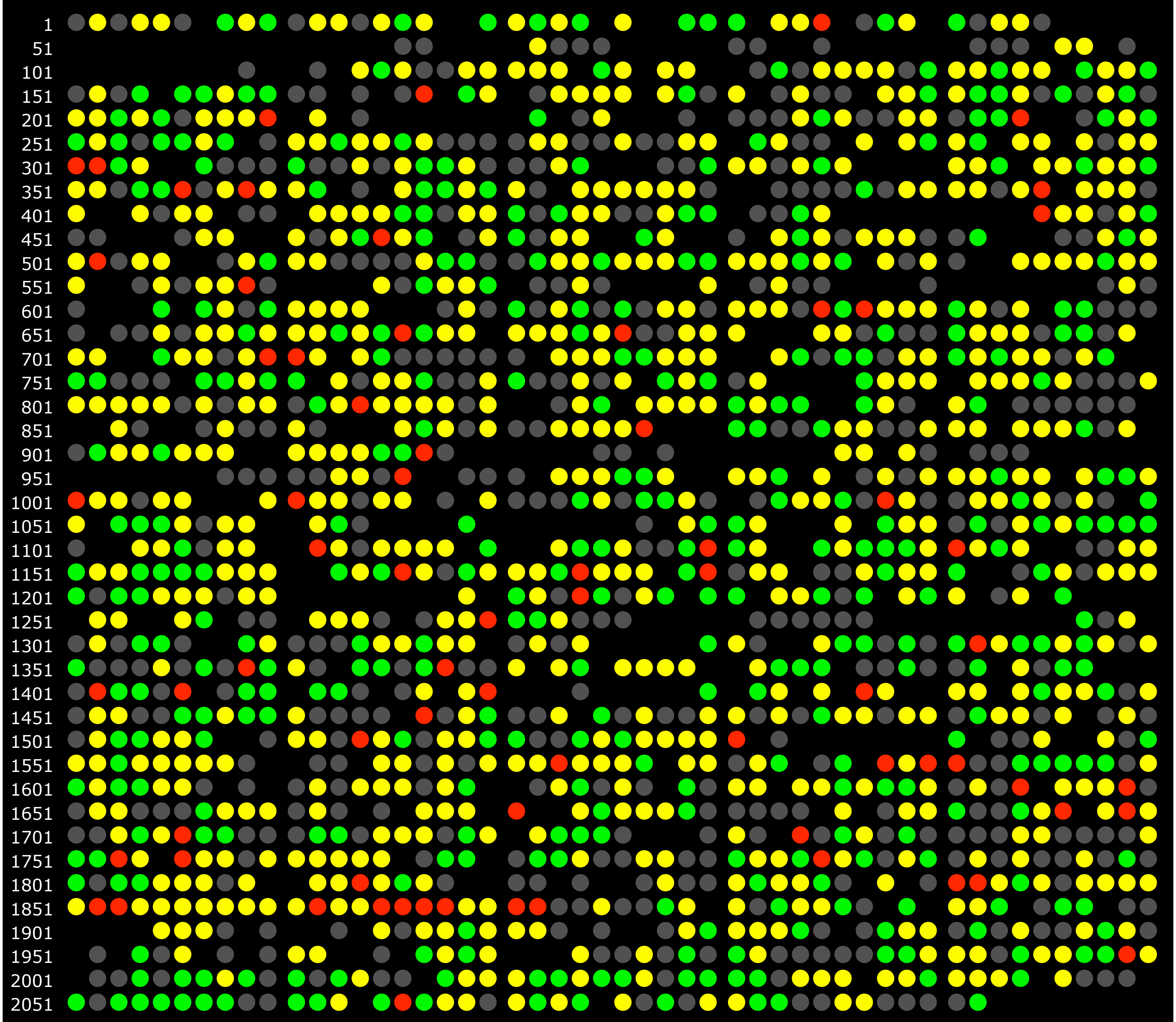

Figure SF2. PgSgFn / Pg : Protein Intensity (PGN)

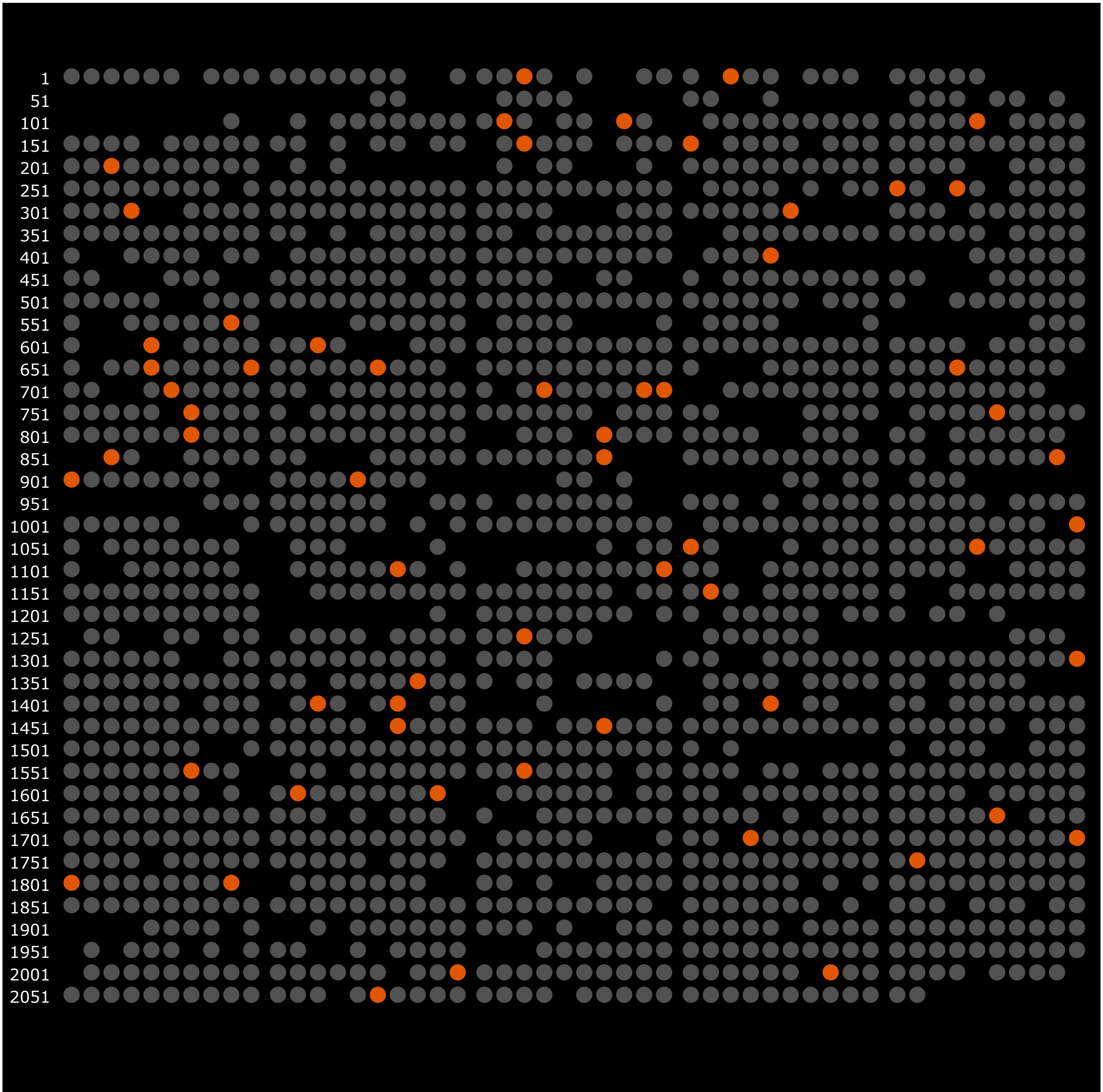

Figure SF3. sec-Pg in PgSgFn: Spectral Count (PGN)

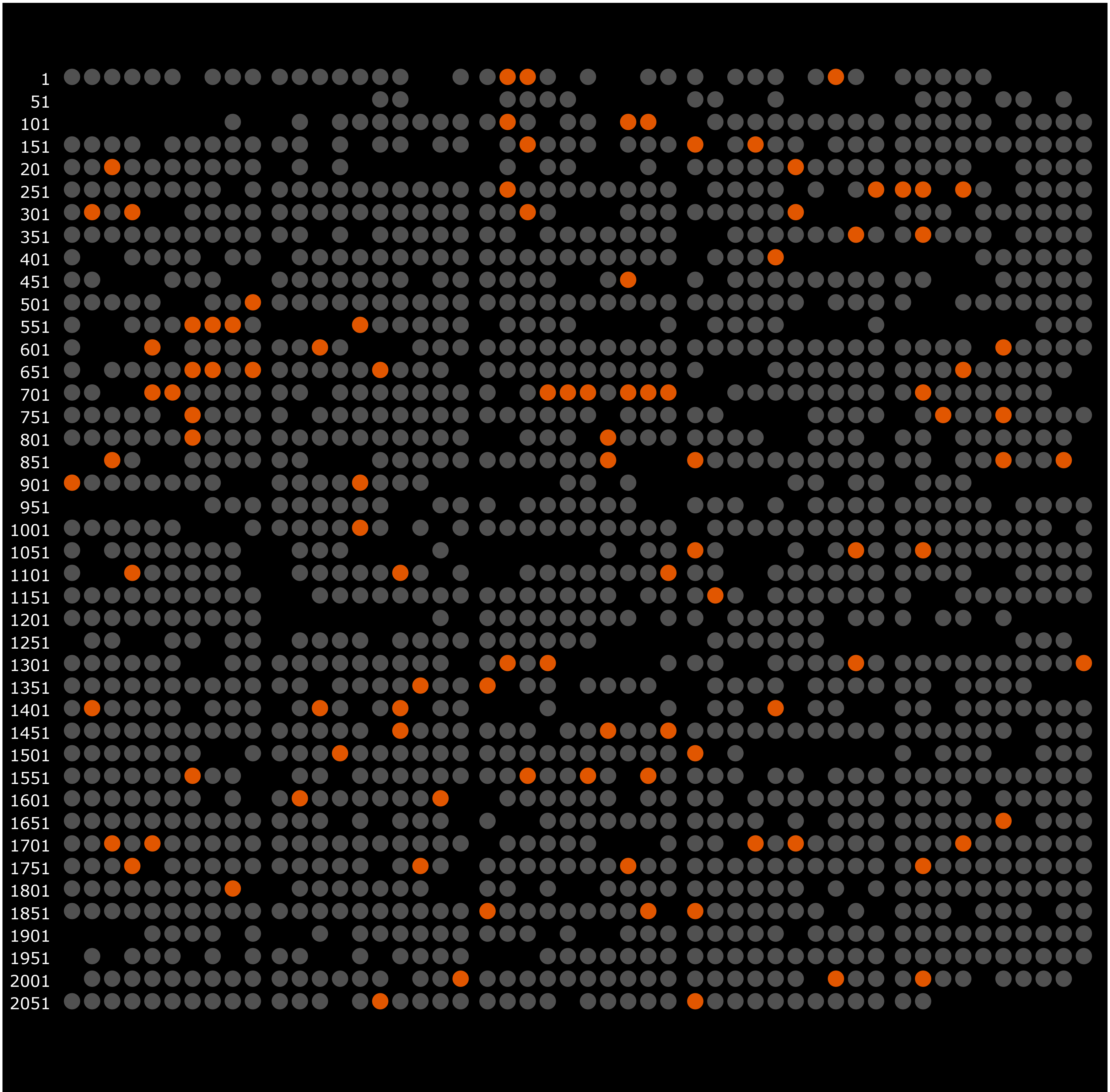

Figure SF4. sec-Pg in Pg: Spectral Count (PGN)

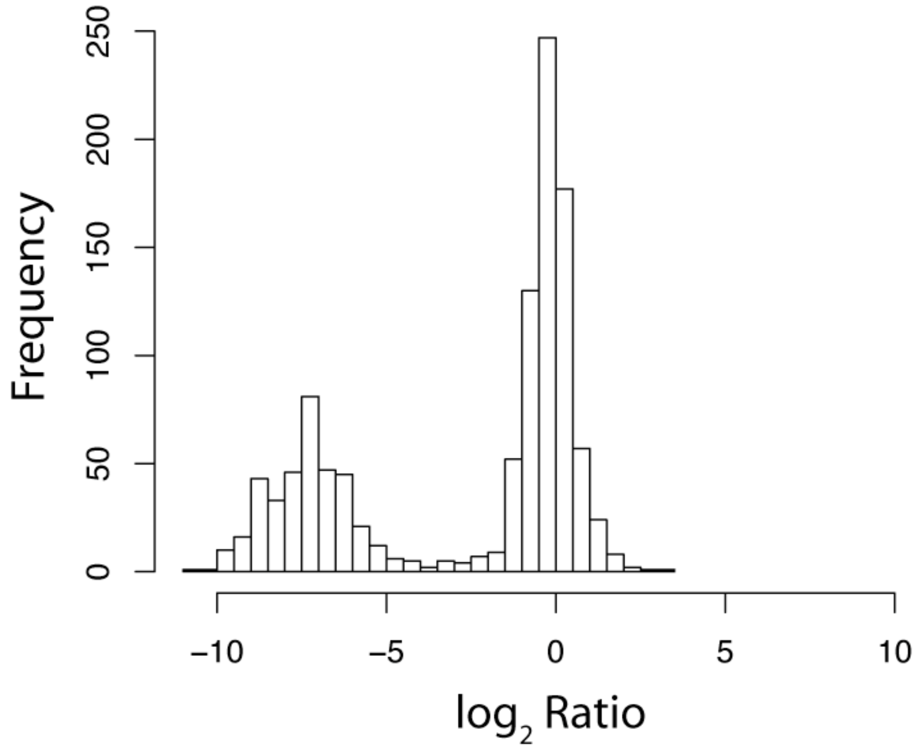

Figure SF5. PgSgFn / Pg by summed intensity prior to removal of data with inappropriate baselines.

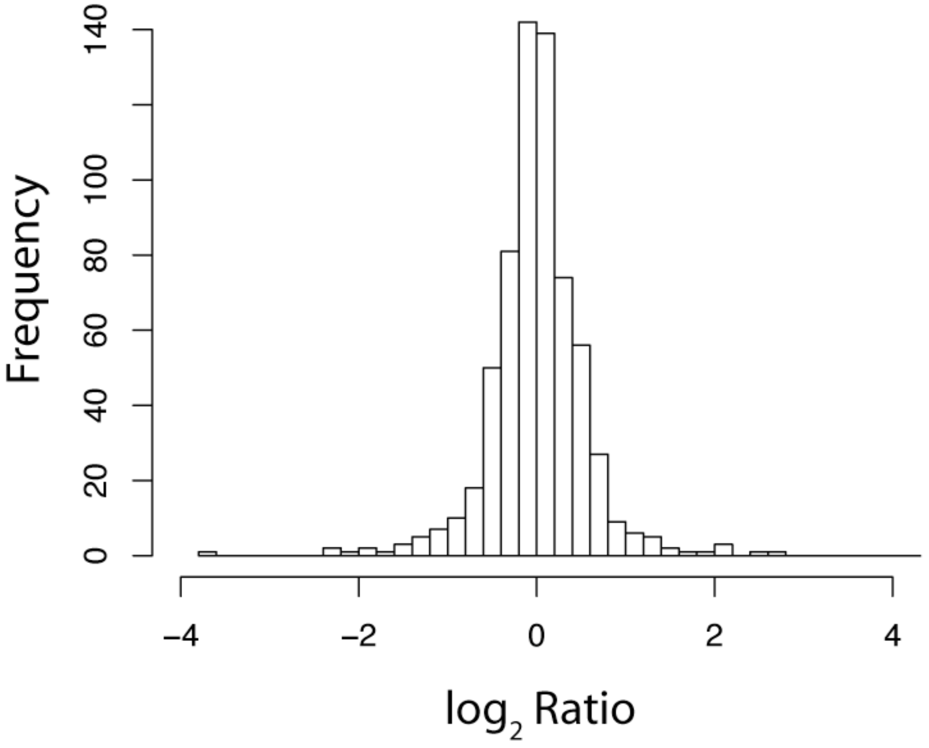

Figure SF6. PgSgFn / Pg by summed intensity after removal of data with inappropriate baselines.
